# Mechanistic Insights Revealed by the Crystal Structure of a Histidine Kinase with Signal Transducer and Sensor Domains

**DOI:** 10.1371/journal.pbio.1001493

**Published:** 2013-02-26

**Authors:** Chen Wang, Jiayan Sang, Jiawei Wang, Mingyan Su, Jennifer S. Downey, Qinggan Wu, Shida Wang, Yongfei Cai, Xiaozheng Xu, Jun Wu, Dilani B. Senadheera, Dennis G. Cvitkovitch, Lin Chen, Steven D. Goodman, Aidong Han

**Affiliations:** 1 State Key Laboratory for Cellular Stress Biology, School of Life Sciences, Xiamen University, Xiangan, Xiamen, China; 2 Department of Molecular and Computational Biology, University of Southern California, Los Angeles, California, United States of America; 3 Department of Biology and Technology, Tsinghua University, Beijing, China; 4 Division of Biomedical Science, Herman Ostrow School of Dentistry of University of Southern California, Los Angeles, California, United States of America; 5 Dental Research Institute, Faculty of Dentistry, University of Toronto, Toronto, Ontario, Canada; UMDNJ/Robert Wood Johnson Medical School/HHMI, United States of America

## Abstract

Two-component systems (TCSs) are important for the adaptation and survival of bacteria and fungi under stress conditions. A TCS is often composed of a membrane-bound sensor histidine kinase (SK) and a response regulator (RR), which are relayed through sequential phosphorylation steps. However, the mechanism for how an SK is switched on in response to environmental stimuli remains obscure. Here, we report the crystal structure of a complete cytoplasmic portion of an SK, VicK from *Streptococcus mutans*. The overall structure of VicK is a long-rod dimer that anchors four connected domains: HAMP, Per-ARNT-SIM (PAS), DHp, and catalytic and ATP binding domain (CA). The HAMP, a signal transducer, and the PAS domain, major sensor, adopt canonical folds with dyad symmetry. In contrast, the dimer of the DHp and CA domains is asymmetric because of different helical bends in the DHp domain and spatial positions of the CA domains. Moreover, a conserved proline, which is adjacent to the phosphoryl acceptor histidine, contributes to helical bending, which is essential for the autokinase and phosphatase activities. Together, the elegant architecture of VicK with a signal transducer and sensor domain suggests a model where DHp helical bending and a CA swing movement are likely coordinated for autokinase activation.

## Abstract 2

A crystal structure reveals an elegant mechanistic switch whereby helical bending and catalytic domain rotation allow self-activation of a histidine kinase during a bacterial stress response.

## Author Summary

Two-component signal transduction systems (TCSs) are promising targets for new antimicrobial research because they help bacteria and fungi adapt and survive. One of the main components of TCSs is a sensor histidine kinase (SK), which relays extracellular signals to intracellular pathways. Despite intensive research, a full-length structure of an SK has yet to be solved. In this study, we report the first crystal structure of the complete cytoplasmic region of VicK, an important SK in the tooth decay pathogen *S. mutans*. VicK is composed of several domains (HAMP, PAS, DHp, and catalytic and ATP binding domain [CA]) in addition to a short transmembrane domain. We find that the dimeric VicK protein has an elegant rod-shaped structure with the domains linearly connected like beads on a string. The structure suggests that VicK kinase activates itself by helical bending of the DHp domain and coordinated swinging around of the catalytic CA domain to engage with the target histidine. Structure-based mutagenesis experiments also helped us to identify key residues that are required for VicK's opposing phosphatase activity. Our studies of the multi-modular VicK protein suggest a sequential kinase activation model that may involve helical bending of the DHp domain and repositioning of the CA domains.

## Introduction

Protein phosphorylation is an essential signal carrier. Bacteria respond to transient living environments through transmembrane-integrated sensor histidine kinases (SKs), which act in concert with their intracellular cognate response regulators (RRs) to elicit necessary adaptive responses that are critical for their survival and virulence. The SKs and RRs have evolved into a two-component signal transduction system (TCS), whereby stimulation of the SK autophosphorylates at a conserved histidine residue to initiate a signaling cascade [1]. The phosphoryl group is transferred from the SKs to their cognate RRs, some of which lead to quickly reprogram bacteria by altering the transcriptional level of specific downstream target genes [2]. Because of the wide prevalence in bacteria and fungi, TCSs have been considered attractive targets for the development of potential therapeutics to control bacterial infections [3],[4].

Sensor domains are key modulators for SKs [5]–[7]. PAS domains (acronym for Per, ARNT, and SIM from *Drosophila*) are sensors in a majority of SKs, which respond to alterations in the redox potential, oxygen content, light, and small molecules in their environments [8],[9]. Because of their broad involvement in biological processes, the structure and function of the PAS domains in interactions with a variety of ligands have been extensively studied [10],[11]. The oligomeric dynamics of PAS domains in cooperation with local conformational changes can affect the stability of the entire SK, which is thought to be part of the mechanism of signal sensing and transduction [10],[12].

The enzymatic activities of SKs are modulated by HAMP domains, which are commonly found in histidine kinase, adenylyl cyclase, methyl-accepting chemotaxis, and phosphatase proteins [6]. Structural dynamics of the HAMP domain are believed to mediate transmembrane signal transductions [13]. The NMR structure of a HAMP domain from the putative transmembrane receptor Af1503 in *Archaeoglobus fulgidus* revealed an unusual knobs-to-knobs interhelical structure, which suggests a coordinated helical rotation model for the HAMP domain in signal transmission [14]. This model was further supported by a series of experimental structures of the HAMP mutants and detailed bioinformatics analyses [15],[16]. Several other laboratories reached a consensus conclusion using homologous HAMP domains from different TCSs that the dynamic properties of the HAMP domains are essential in mediating signal transduction [17]–[22].

The C-terminal catalytic and ATP-binding domain (CA) of the SKs, also called HATPase_c, in addition to the DHp domain (dimerization and histidine phosphorylation domain), phosphorylates a conserved histidine residue in the middle of DHp helices [2],[23]. The active site of an SK is assembled with the CA and DHp domains [23],[24]. The plasticity of the DHp domain and CA positioning is implicated in the on/off switch of the temperature sensor DesK kinase from *Bacillus subtilis* [25].

VicRK is a well-characterized TCS that is highly conserved and essential for survival and virulence in a wide range of firmicute bacteria, including *Streptococci*, *Bacilli*, and *Staphylococci* [26]. Because of its critical role in cell wall synthesis, VicRK is also referred to as WalRK [27]. The VicRK in *S. mutans*, which is an important pathogen in caries etiology, regulates acid production and tolerance conducive to dental caries, including proton expulsion (F_1_F_0_-ATPase) [28]–[31]. VicK belongs to the type IA family based on sequence conservation of the DHp and CA domains [32]. In addition, VicK has one HAMP domain and one PAS domain. However, the function of the PAS domain is not clear because no ligand has been identified [26]. Recently, a deletion experiment indicated that the HAMP and PAS domains are essential for VicK phosphatase activity [33].

Given the vital role of TCSs in bacterial adaptation and pathogenicity, decades of research have focused on the molecular mechanisms of TCS signaling cascades [34]–[36]. Although the structure and functions of individual domains are well known, the signal transduction mechanisms remain largely unknown. Toward this end, we determined a crystal structure for a streptococcal VicK that harbors HAMP transducer and PAS sensor domains. Our crystal structure of the nearly full-length VicK comprises an elegant construction of multiple domains and reveals novel insights into the molecular mechanisms of the VicK histidine kinase.

## Results

### Overall Structure

**Figure 1:**
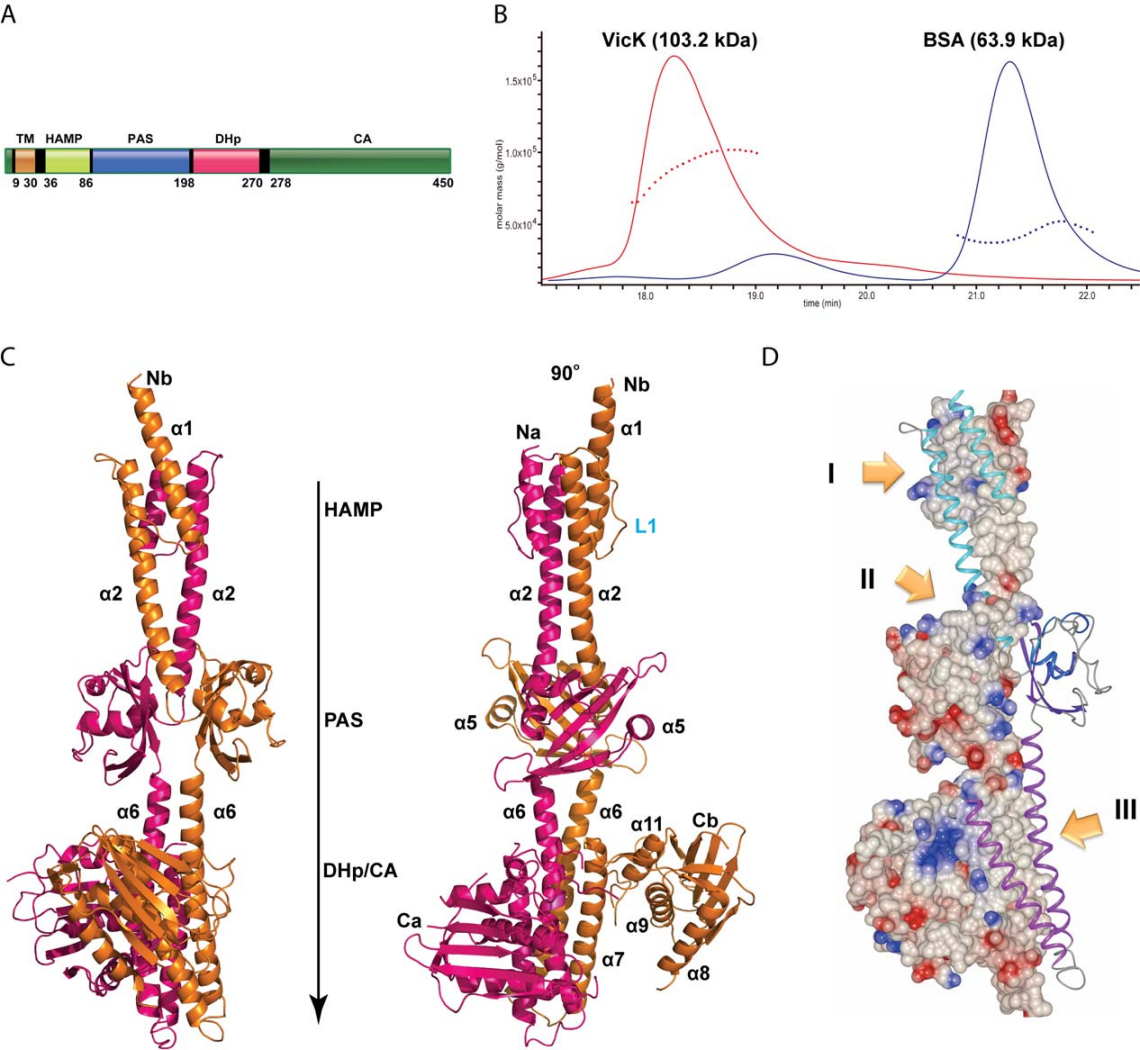
Overall structure of VicK. (A) Domain architecture of the full-length VicK. The numbers below are the breakpoints of functional domains, which are colored differently and labeled on top. (B) Molecular weight of VicK in solution measured by multiangle light scattering (MALS). BSA was used as a control colored in blue and VicK in red. The time range was taken from the HPLC. (C) Overall structure of VicK presented in ribbon. Two monomers are colored in magenta and gold. The corresponding domains are indicated. Helices α1–11 are labeled. Na and Nb indicate the N termini of the monomer *A* or *B* while Ca and Cb indicate their C termini. (D) Electrostatic potential surface of the VicK monomer. Red to blue colors represent negative to positive charged areas (−0.75 to +0.75, CCP4mg). VicK monomer *B* is presented in ribbon with CA domain deleted for better clarity. Contact areas (I–III) of the VicK dimeric interface are marked in yellow arrows.

The *S. mutans* VicK has one transmembrane domain (TM, aa 9–30) that anchors itself to the cytoplasmic membrane (Figure 1A). Following the TM domain, a HAMP signal transducer domain and PAS sensor domain are directly connected to the CA domain through a DHp domain. As several attempts to express full-length VicK (aa 1–450) resulted in insoluble protein, the entire intracellular region (TVicK, aa 31–450) was successfully purified and crystallized. TVicK had a K_m_ of 44.5 µM and a K_cat_ of 0.413 min^−1^ (Table S1). Its kinetic parameters were similar to the TM-truncated VicK homologue of *S. pneumoniae* [33]. Static light scattering revealed a single species of VicK at a molecular weight of 103.2 kDa, indicating that the holoenzyme exists as a stable dimer in solution (Figure 1B).

VicK crystals diffracted slightly better than 3 Å; however, because of strong anisotropy, the final structure was refined up to only 3.3 Å (Table S2). The overall structure of VicK comprises a dimer in the shape of a long slim rod (Figure 1C). The longest dimension of this molecule is nearly 150 Å (Cα distance). Each monomer contains a series of helices (α1–α11). One asymmetric unit contains two VicK dimers. Remarkably, the total buried surface area is 7590.8 Å^2^ upon VicK dimerization. The dimer interface consists of three tight hydrophobic contact patches (Figure 1D, I–III). In addition, the HAMP, PAS, and DHp domains are organized as dimers, which are connected by long, straight coiled-coils of helices α2 and α6 (Figure 1C). The C-terminal ends of the VicK dimer harbor two monomeric CA domains (Figure 1C). The N-terminal end of monomer *A* (*Na*, aa 31–37) and both C-terminal tails (*Ca* or *Cb*, aa 433–450) are disordered.

### Structure of the HAMP Domain

**Figure 2:**
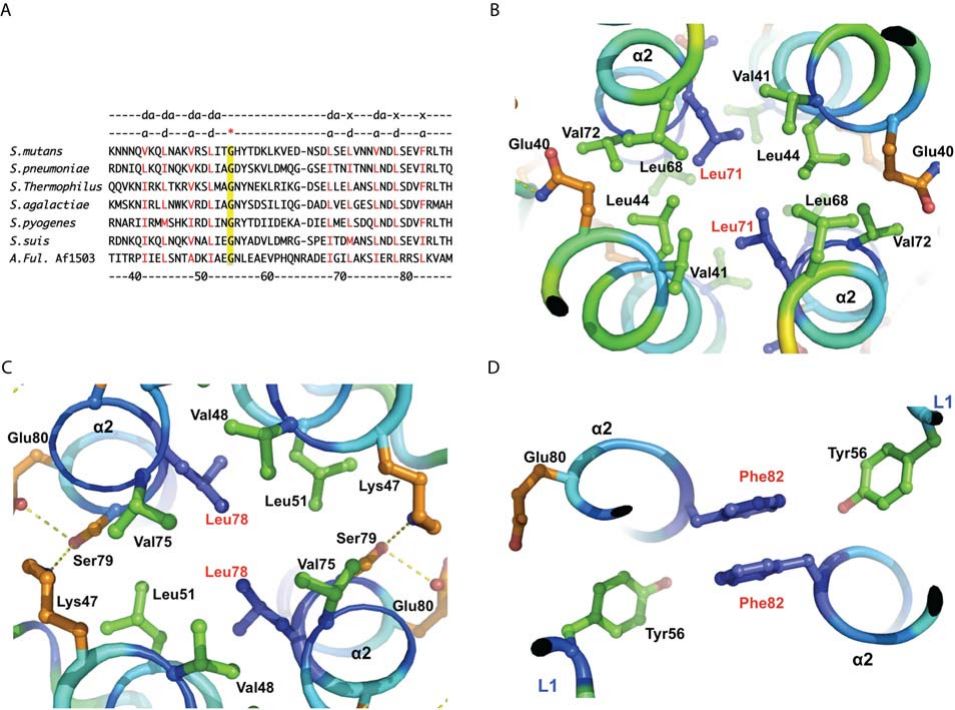
Structure of VicK HAMP domain. (A) Sequence alignment of HAMP domains from representative SKs of *Streptococci* together with *A. fulgidus* Af1503. The positions of amino acids below are labeled according to *S. mutans* VicK. The *a* and *d* positions defined according to the classical coiled-coil are indicated on the top and highlighted in red. The x and da positions are also labeled according to the unique knobs-to-knobs model defined by Hulko et al. [14]. The red star indicates a conserved Gly in all HAMP domains and highlighted in yellow. (B), (C), and (D) The hydrophobic knobs-to-knobs contacts in the central HAMP domain. The critical residues are shown as blue sticks and labeled in red.

The HAMP domain (aa 36–86) is located at the uppermost position within the N-terminal region of the VicK structure (Figure 1C). Helices α1 and α2 of each VicK monomer form a parallel four-helical coiled-coil that is connected with loops L1 (Figure S1A). *S. mutans* HAMP shares approximately 45% identity to other Streptococci; however, it shows little (∼5%) identity to *A. fulgidus* Af1503, although the critical residues at positions *a* and *d* are mostly conserved (Figure 2A).

The helical interactions within the HAMP domain can be grouped into three shells (Figure S1B). The outer shell is formed by hydrophilic and polar residues at positions *b*, *e*, and *g* of the coiled-coil in addition to two residues from loop L1 (Figure 2A). The residues at the *b* and *g* positions in helix α1 are rich in basic residues with long side chains, whereas the corresponding positions in α2 are rich in polar residues (Figure S1B, gold). The middle shell is formed with hydrophobic residues, including leucine, isoleucine, and valine (Figure S1B, green). These residues form the canonical knobs-into-holes packing of coiled-coils [37]. Central to the VicK HAMP bundle are the hydrophobic residues that are all in van der Waals contacts and display knobs-to-knobs or x-layer packing (Figures 2A and S1B, blue).

The HAMP outer shell is distinct with three bound rings visible on the electrostatic surface (Figure S1C). Within this bundled structure, there are three pairs of hydrophobic residues in the knobs-to-knobs packing solely from two α2 helices. Two Leu71 residues compose the inside core of the first ring (Figure 2B) and two Leu78 residues are inside the core of the second ring (Figure 2C). Strikingly, the Phe82 pair forms π-π stacking inside the third ring, which is further stabilized by two Tyr56 residues from two L1 loops (Figure 2D). Indeed, Tyr56 is completely conserved in *Streptococci*, whereas Phe82 can be replaced by leucine in Af1503 (Figure 2A). In other HAMPs, position 82 can be Ile, Val, or Leu, whereas position 56 is typically Leu, Phe, or Tyr [15]. These residues are also capable of making van der Waals contacts if placed into the VicK HAMP domain (unpublished data). Notably, Gly54 is absolutely conserved in these HAMP homologs (Figure 2A).

### The PAS Domain Dimer

**Figure 3:**
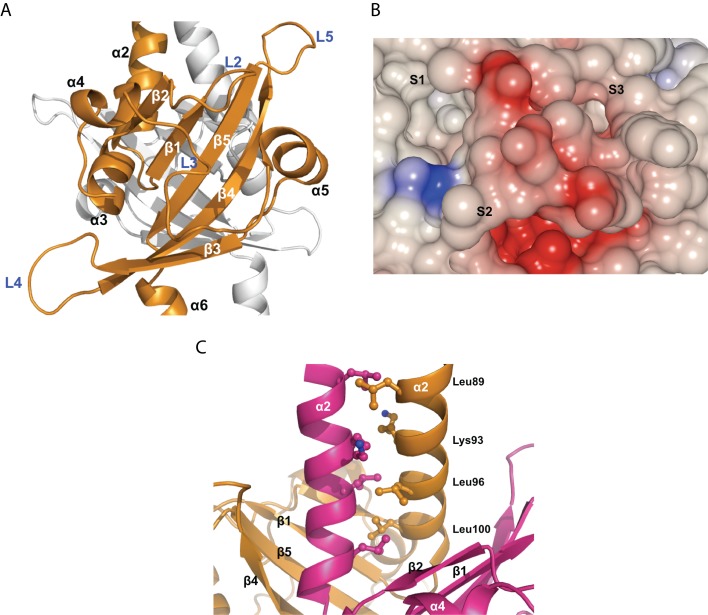
Structure of VicK PAS domain. (A) A canonical PAS monomer of VicK shown in gold ribbon. Another PAS monomer is fainted for better clarity. β-strands, helices, and loops are labeled according to the overall structure of the VicK molecule. (B) Electrostatic surface of the VicK PAS domain monomer. The color scheme is the same as Figure 1D. Three potential sockets (S1–3) for ligands to bind are labeled. (C) Leucine-zipper interface of the VicK PAS domain dimer with critical residues shown in sticks and labeled on the right.

The PAS domain (aa 87–198) in VicK, which is located downstream of the HAMP domain, adopts a canonical fold (Figure 3A). Both PAS domains can be well aligned (root mean square deviation [rmsd] of 1.2 Å) except for the large shift of loop L5 (Figure S2A). The five β-strands form a core of β-sheets, which are sandwiched by two α2 helices on one side and helices α3–5 on the other. The loop L3 and the connected helix α5 form a surface layer on the top of the β-sheet with two flexible loops, L4 and L5, on both edges. Three substantial binding pockets (S1–3) with a large cavity and inside tunnel are observed on this surface (Figure 3B). The tunnel is lined by mostly hydrophobic residues, including Leu108, Ile116, Leu127, Ile142, Phe178, and Leu195.

The VicK PAS domains form a unique dimer, which is mediated by a leucine-zipper (Figure 3C). This leucine-zipper further forms hydrophobic networks with the canonical PAS domain, which suggests that the leucine-zipper is an integral part of the VicK PAS domains (Figure S2B). The residues Leu89, Leu96, Leu100, and Lys93 are in key positions to make van der Waals contacts (Figure 3C). The substitution of Leu100 with arginine was previously shown to disrupt VicK autokinase activity in *S. pneumoniae* [38]. It is likely that the L100R mutation destabilizes the dimerization of the VicK PAS domains, which sheds light on the functional importance of PAS dimerization.

To look for clues of ligands for VicK PAS domains, we compared the VicK PAS domains with representative structures of all ligand-bound PAS domains according to a recent comprehensive review by Henry and Crosson [11]. Despite their limited sequence identities (6%–10%), all PAS domains can be structurally aligned to VicK with low rmsd of 2–2.5 Å (Figures S3 and S4). The relatively large shifts were observed in loops L3 to L5 and helix α5 of the PAS domains of NifL, FixL, DcuS, and PhoQ, which form pockets for a variety of ligands to bind. Therefore, the VicK PAS domain may bind some ligands differently from these PAS domains because it has a unique cavity and tunnel properties.

### The Overall Structure of the Histidine-Specific ATPase

The C-terminal end of VicK (aa 199–450) contains a histidine-specific ATPase, which is often divided into one DHp domain and one CA domain with a short linker (aa 270–278) (Figure 1A). The dimeric architecture of these domains looks like a butterfly, wherein the DHp domain (aa 199–269) is a four-helix bundle of helices α6 and α7 with a phosphoryl receptor histidine located in the middle (Figure S5A). Surrounding this helical core are two CA domains with a layer of four helices (α8 to α11) on a layer of β-sheets (β7 to β12), wherein α10 is a short helix (Figure S5B). The two loop regions, L7 (aa 303–308) and L11 (aa 391–395) in the CA domain, called the lid, could not be well defined because of a weak electron density and are labeled with light dashed lines. One linker (aa 270–274), which connects the DHp and CA domains, was also disordered (Figure S5A, the monomer in magenta).

Both CA domains (aa 278–450) of VicK adopt a classical histidine kinase fold and are well aligned with an rmsd of 1.3 Å despite the missing loop L11 (Figure S5B). The CA domain of VicK is similar to HK853 (rmsd of 1.7 Å) except for structural differences in loops L7 and L8 in addition to the loop L11 region (Figure S5C). We could not clearly define ATP binding because of the weak electron density at the current resolution.

### Helical Bending in the DHp Domain

**Figure 4:**
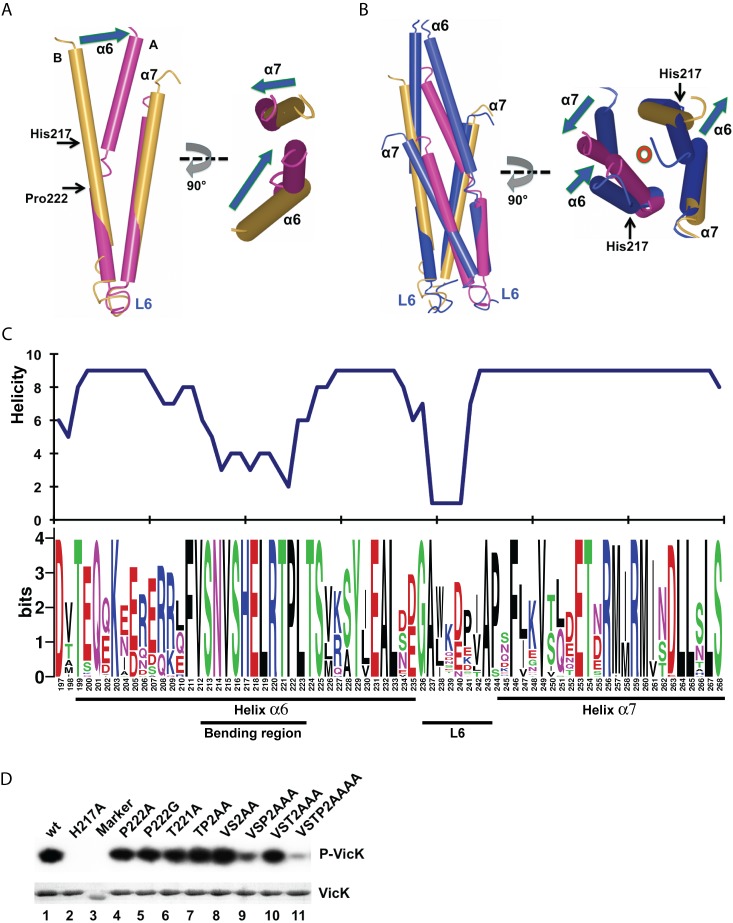
Helical bending property of the DHp domain. (A) Structural alignment of two VicK DHp chains; (B) The VicK DHp domain aligned with *T. maritima* HK853. The HK853 DHp domain is colored in blue. VicK His217 and Pro222 are indicated with arrows. Bottom parts of helices (aa219–255) of the VicK DHp domain were used for alignments. The right is a top view of these aligned structures shown on the left. The blue arrows indicate direction and relative distance of shifts. The red circle represents a central axis parallel to the DHp domain and going into the paper. (C) Conservation and helicity of the DHp domain. The bottom part is a conservation logo using over 40 non-redundant histidine kinases. The top part is the helicity of the DHp domain calculated by Phyre described in Materials and Methods. (D) Autokinase analysis using isotope γ^32^P-ATP. The wt VicK and its mutants were analyzed on 15% SDS-PAGE and stained with coomassie blue as shown in the bottom gel. The top gel is an autoradiograph of their autokinase activities of these proteins. A protein marker served as a background control (lane 3).

Unlike the HAMP domain, the DHp domain forms an anti-parallel four-helical coiled-coil. Interestingly, the two monomers of the DHp domain bear an asymmetric fold (Figure 4A). The two monomers are well aligned with an rmsd of 2.2 Å for the bottom part of the coiled-coil (aa 219–255), and the relatively large rmsd of the alignment is mostly caused by flexibility of loop L6. However, the upper parts of helices α6 and α7 are remarkably different, wherein helix α6 bends ∼25° at Pro222 toward the central DHp axis and α7 moves ∼11° away in the opposite direction to avoid a possible clash.

When aligning the VicK DHp domain with *T. maritima* HK853, which is a symmetric four-helix coiled-coil, we observed similar bending of monomer *A* (Figure 4B). The bending is coordinated with shifts of the upper helices, whereas the bottom helices remain stationary, and the upper part of helix α6 of monomer *A* moves toward the center axis, which possibly drives the other helices away.

### Mutations in the DHp Domain Affect Kinase Activity

Helix α6 of the DHp domain has an intrinsic plasticity for bending (Figure 4C). The bending region is absolutely conserved among over 50 VicK homologs in *Streptococci*, *Lactobacilli*, *Lactococcus*, and *Enterococci* (Figures 4C, the bottom and S6). Consistently, the DHp domain region (aa 211–225) was predicted to have low helical probability (Figure 4C, the top). Therefore, we hypothesized that the low helical propensity of the DHp domain may play a role in His217 phosphorylation. To test this, we mutated Pro222 to glycine and measured its autokinase activity (Table S1). The P222G mutant retained full activity when compared to wild-type (wt) VicK, as did the T221A mutant. We continued to mutate residues in the bending region, including Val212, Val215, Ser213, and Ser216 to alanine (VS2AA), which is statistically favorable in an α-helix [39]. All mutants, including the combined mutants VSP2AAA, VST2AAA, and VSTP2AAAA, bound *S. mutans* VicR similarly to wt VicK, which suggested that they likely retain the correct conformation (Figure S7).

The autokinase activity of the single and combined mutants described above was analyzed using γ^32^P-ATP (Figure 4D; Table S1). As the Thr221 and Pro222 mutants (T221A, P222A, P222G, and TP2AA) retained nearly full activity compared to wt VicK, the two combined mutations of VSP2AAA and VSTP2AAAA showed significantly reduced activity. Although we cannot rule out other effects from multiple sites of mutation, these data are consistent with the model that the low helical propensity of the DHp domain in addition to Pro222 are important for VicK autokinase activity.

### The Proline Is Essential for Phosphatase Activity

**Figure 5:**
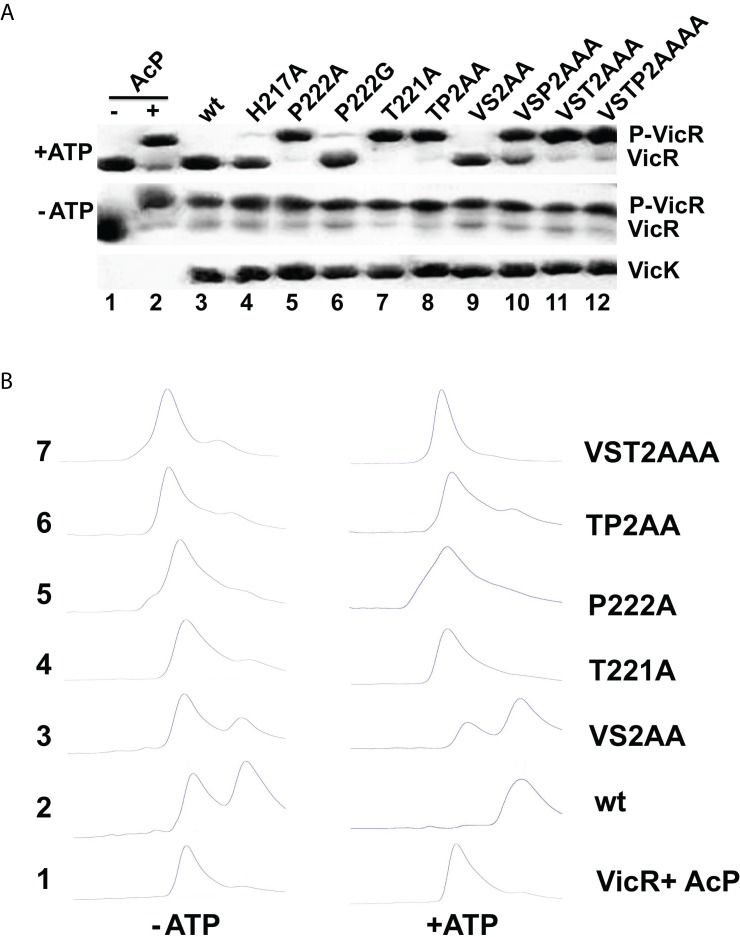
Proline and threonine are essential for VicK phosphatase activity. (A) VicR dephosphorylation examined in PMS. Phosphorylated VicR was treated with the same amount of VicK wt or its mutants as shown in the bottom gel. Native and phosphorylated VicR were separated in PMS as labeled on the right of the top two gels, where in one series of reactions additional 5 mM ATP was used (the top 1 gel). (B) VicK phosphatase activity analyzed by HPLC. AcP-treated VicR was incubated with VicK in the reaction buffer with 5 mM ATP (right) and without ATP (left). VicK phosphatase activity was analyzed by the phosphorylation state of VicR on HPLC.

As VicK is a multifunctional enzyme, we tested whether the helical bending region of the DHp domain is important for phosphatase activity. Here, we used Phos-tag gel mobility shift assay (PMS) to detect the phosphorylated *S. pneumoniae* VicR. As a control, over 90% of VicR was phosphorylated by acetyl-phosphate (AcP), which resulted in a mobility retardation shift (Figure 5A, lane 2). Further incubation with wt VicK completely removed the phosphate group from VicR (lane 3, top gel). It is interesting to note that in the absence of 5 mM ATP, little dephosphorylation of VicR was observed (lane 3, middle gel), which suggested that ATP is required for VicK phosphatase activity.

We tested the phosphatase activity of the DHp mutants described above. To our surprise, the single proline or threonine mutations (P222A and T221A) abolished VicK phosphatase activity (Figure 5A, lanes 5 and 7, top gel). In contrast, the P222G and VS2AA mutants, similar to wt VicK, dephosphorylated VicR within the time course of this assay (lanes 6 and 9). Consistently, the combined mutants VSP2AAA, VST2AAA, and VSTP2AAAA, which contain Pro222 and Thr221 substitutions, also showed little phosphatase activity (lanes 10–12). It is interesting to note that the VSP2AAA mutant had substantially more phosphatase activity than the single P222A mutant likely because VSP2AAA mutant lost partially kinase activity (Figure 4D).

We also analyzed some of these mutants using high performance liquid chromatography (HPLC). Phosphorylated *S. pneumoniae* VicR eluted at ∼7.6 min compared with the native VicR at ∼8.8 min (Figure 5B, run 1). After incubation with VicK in the presence of ATP, phosphorylated VicR completely shifted back to 8.8 min (Figure 5B, run 2). A clear conversion to unphosphorylated VicR was also observed when incubated with the VS2AA mutant (Figure 5B, run 3). All other VicK mutants, including T221A, P222A, TP2AA, and VST2AAA, failed to dephosphorylate VicR (Figure 5B, runs 4–7).

Together, our data suggest that Pro222 and its neighbor, Thr221, are the key residues in the bending region required for the phosphatase activity of VicK.

### Active State of the CA Domain

**Figure 6:**
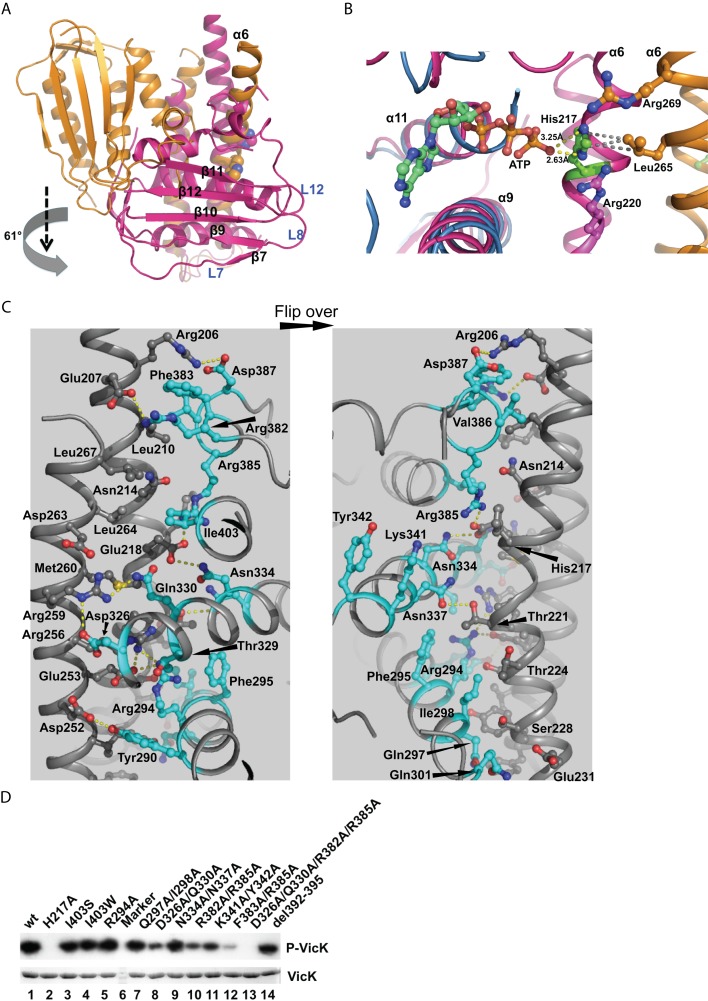
Analyses for autokinase activation. (A) Structural alignment of two VicK monomers in reference to the DHp domain. The alignment was carried out as described in Figure 4A. The vertical movement is indicated with a black arrow in parallel to the DHp domain while a grey arrow indicates a rotation. (B) The VicK CA domain (magenta) aligned to *B. subtilis* YycG (light blue) in the presence of ATP (green and gold sticks). His217 is represented by green sticks and several other residues are shown in magenta and gold sticks. The contacts between the distances of 2.5–3.5 Å are highlighted with yellow dashed lines and those between 3.5–4.5 Å with grey dashed lines. (C) Residues critical for the interaction between the DHp and active CA domains. The VicK Cα chain is colored in gray ribbon and residues from the CA domain critical for this interface in cyan sticks. The critical residues from the DHp domain are highlighted in grey sticks. The right panel is a flip-over view of the left. (D) Mutations of key residues in the active interface affected autokinase activity. The amount of wt VicK and mutants used in each reaction were analyzed by 15% SDS-PAGE and stained by coomassie blue as shown in the bottom gel. A protein marker served as a background control (lane 6). The gel containing phosphorylation forms of VicK was exposed to an X-ray film as shown in the top gel.

Although the overall conformation of each CA domain in VicK is the same, their positions relative to the DHp domain show dramatic differences, which generates an asymmetrical ATPase dimer (Figure 6A). The CA domain rotates ∼61° and further translates ∼20 Å down along an axis parallel to the DHp domain. When aligning the CA domain with the symmetric dimeric structure of *T. maritima* HK853, we found that monomer *A* takes completely different positions (Figure S8, magenta).

The large shift of the CA domain of monomer *A* moves its active site to the *in cis* phosphoryl acceptor His217. Recently, a crystal structure of the *B. subtilis* YycG CA domain bound to ATP was solved, which is 47% identical to the CA domain of *S. mutans* VicK [40]. These two structures of the CA domains aligned well with an rmsd of 1.47 Å of all backbone atoms, which is where the ATP from the structure of the YycG CA domain was modeled nicely in the active pocket of the VicK CA domain. To our surprise, we found that the γ-phosphate of ATP approached His217 to form two hydrogen bonds with εΝ or δN of His217 (Figure 6B, yellow dashed lines). In addition, Leu265 from monomer *B* is close to His217 to form van der Waals interactions (Figure 6B, grey dashed lines). Arg220 of monomer *A* and Arg269 of monomer *B* are also in close contact with His217.

In addition to the ATP binding pocket, the CA domain positions itself toward the DHp domain to form a large interface (Figure S9). Many residues are involved in the direct interaction between the DHp and CA domains (Figure 6C). Arg294, Asp326, Gln330, Asn334/337, and Arg385 of the CA domain form hydrogen bonds with Glu218, Thr221, Glu253, Arg256, and Arg259 residues around the middle region of the DHp domain. In addition, Arg382 and Asp387 form hydrogen bonds with Arg206 and Glu207 at the upper part of the DHp domain. Phe295, Ile298, Phe383, and Ile403 of the CA domain also form van der Waals contacts with Asn214, Thr224, Ser225, and Tyr229 of the DHp domain. Ile403, which is located on the back of the ATP binding pocket of VicK, is one of the key residues that contribute to the hydrophobic interaction with Leu267 and the aliphatic side chain of Glu218.

To test whether these interactions are important for autokinase activity, we generated a series of mutants and analyzed their autokinase activity (Figure 6D). The Arg294, Gln297, Ile298, Asn334, and Asn337 mutations (R294A, Q297A/I298A, and N334A/N337A) were nearly as active as wt VicK (Figure 6D, lanes 5, 7, and 9). In contrast, mutations of Asp326, Gln330, Arg382, Arg385, and Phe383 (D326A/Q330A, R382A/R385A, and F383A/R385A) dramatically suppressed VicK autokinase activity (Figure 6D, lanes 8, 10, and 12), whereas the dual mutations of D326A/N337A and R382A/R385A completely eliminated VicK autokinase activity (Figure 6D, lane 13). As references, two mutations in the ATP-binding pocket (K341A/Y342A) and a deletion of the first G loop (del392–395, RAQG) negatively affected autokinase activity (Figure 6D, lanes 11 and 14). Two Ile403 mutations I403W and I403S did not significantly affect VicK autokinase activity (Figure 6D, lanes 3 and 4). Interestingly, the I403W mutation was found to dramatically increase autokinase activity in HK853 (I448W) [23].

Together, the CA domain of monomer *A* can position itself toward the DHp domain to form a large interface, which is further centered by two groups of key residues. One group is D326/Q330, which forms hydrogen bonds with Arg259 of the DHp domain. Another group is R382 and R385, which form hydrogen bonds with Glu207 and Glu218 of the DHp domain. F383 inserts into a hydrophobic pocket in the DHp domain and further stabilizes the interactions between the CA and DHp domains. Therefore, a mutant (D326A/Q330A/R382A/R385A) that disrupts both groups of key residues completely eliminated the autokinase activity (Figure 6D, lane 13).

### Positioning of Inactive CA Domain

The CA domain of VicK monomer *B* stays away from the DHp domain and represents an inactive state (Figures 1C and S5A). A relatively small interface is formed mainly by van der Waals interactions between Phe383, Ile403, and Leu399 from the CA domain and Phe211, Val215, and Leu264 from the DHp domain, which are further stabilized by several hydrogen bonds between Arg382, Arg385, Asp271, and Glu218 (Figure S10). The buried surface of this interface is 623 Å^2^, which suggests that it may not be sufficiently stable. Phe383 and Leu399 appear to be the most important residues because they insert into helices α6 and α7 for extensive van der Waals interactions with Leu264 and Val215. When compared with *T. maritima* HK853 alone, the VicK CA domain rotates ∼76° along an axis vertical to the DHp domain (Figure S8). Overall, the CA domain remains inactive by positioning itself away from the DHp domain and this interface may act as a hinge.

## Discussion

The ability to mount a rapid response to various stress signals is essential for the adaptation and survival of prokaryotic cells. Some of the TCSs convert these stress signals ultimately to transcriptional reprogramming for necessary adaptive responses. How SKs are activated in response to these stimuli to initiate subsequent signal transduction cascades has been a long-standing question. In this study, we report the crystal structure of the entire intracellular portion of VicK (aa 31–450), which comprises an SK dimer with one signal transducer, one signal sensor, and one intact kinase domain. This structure allows us to dissect the molecular mechanisms underlying SK-mediated signal transduction in prokaryotes.

### VicK Is a Long Rod-Shaped Molecule

Our crystal structure revealed a long rod-shaped VicK holoenzyme (Figure 1). The three modules of the HAMP, PAS, and DHp/CA domains are connected through two groups of straight helices. Such an extended VicK molecule may provide sufficient surface and accessibility for potential ligands or protein partners to interact with.

The long shaped molecules often have the largest radius of gyration and tend to polymerize. VicK and its homologs have one or two integrated TMs rather than being free cytosolic proteins and are localized as clusters within the membrane [26]. In *B. subtilis*, YycG proteins center in the division ring possibly through interactions with FtsZ, a tubulin-like protein [41]. In contrast, approximately 420 dimeric VicK holoenzymes are randomly distributed as clusters throughout the periphery of one *S. pneumoniae* cell [42]. This clustering characteristic indicates that its function might require direct interaction between individual molecules, which is consistent with the physical properties of rod-shaped molecules.

### Signal Transduction by HAMP

A long-standing question is how HAMP domains serve in signal transduction because they often directly connect TMs and extracellular sensors. The HAMP domains may undergo a 26° rotation, which is derived from the unusual knobs-to-knobs packing of the solution structure of Af1503 HAMP, when they receive transmembrane signals [14],[15]. The crystal structure of concatenated HAMP domains from *Pseudomonas aeruginosa* Aer-2 implicates that the conformational dynamics may also serve a role in signal transduction [20]. Consistently, a series of structure and functional experiments have shown that intrinsic thermodynamic instability is required for HAMP signaling [16],[17],[19],[21],[43].

The VicK HAMP domain appears to be a stable four-helix coiled-coil with classical knobs-into-holes interactions, wherein three pairs of hydrophobic residues from helix α2 form a central hydrophobic core with knobs-to-knobs packing, and it is unlikely that structural alternations of the HAMP domain play a central role in signal transmission (Figures 2 and S1B). The VicK HAMP domain is further stabilized by Phe82, which is conserved in the majority of streptococcal species (Figures 2A and S11). However, variable residues, including isoleucine, valine, and occasionally threonine, are present at position 82 in some VicK homologs, which suggests that the stability of HAMP domains may vary in different species.

It is worth noting that the α2 helices of the HAMP domain connect with downstream PAS domains through continuous helices, part of which (aa 86–103) form a fairly rigid leucine-zipper (Figure 3C). This long helical structure most likely serves two purposes: (1) The HAMP domain might be further stabilized by additional interlock packing of the coiled-coil; and (2) The HAMP domain can easily transfer any conformational change down to the PAS domain. In contrast, the connection of the α6 helices between the PAS and DHp domains, particularly a short linker between α6 and β5, is rather flexible. Thus, the VicK molecule also appears to have a potential thermodynamic property for signal transduction from the HAMP or PAS domains to the catalytic CA domains.

### Mobile PAS Domain

The VicK PAS domains form a stable dimer through the short leucine-zipper (aa 89–103) (Figure 3C). In general, canonical PAS domains are rather flexible and readily subjected to ligand-induced conformational changes [9]. The three loops, L3–L5, form a mobile surface that could be regulated by unknown ligands (Figure 3B). Winkler et al. recently demonstrated that deletion of the PAS domain but not a triple mutation (D133N, N136Y, and L140R) of *S. pneumoniae* VicK reduced the autokinase activity and, more dramatically, phosphatase activity [33]. A similar deletion experiment also showed that the PAS domain is essential for the phosphatase activity of *T. maritima* ThkA [44]. Our *S. mutans* VicK structure shows that Leu135 (equivalent to N136 of *S. pneumoniae* VicK) stabilizes helix α4 by interactions with Ile116 and Ile139 (equivalent to L140 of *S. pneumoniae* VicK), which contributes to the hydrophobic cavity of the PAS domain (unpublished data). Therefore, it is likely that an Ile139 to arginine mutation only disrupted potential ligand binding but not dimerization. In contrast, an L100R mutation disrupted dimerization of the PAS domain, and in turn, the kinase activity of *S. pneumoniae* VicK [38].

In ligand-free VicK, helix α5 adapts an open conformation when compared to flavin adenine dinucleotide (FAD)-bound NifL, heme-bound FixL, and malate-bound DcuS (Figure S4). Thus, loop L3 is able to provide a sufficient cavity and tunnel for potential ligands to bind. Interestingly, when aligned with PhoQ, helix α4 and loop L3 of the VicK PAS domain adapt significantly different conformations and the trajectory of helix α5 is similar. Unfortunately, despite our efforts, no ligand specific to the VicK PAS domain has been experimentally identified. Only when such ligands are identified will it be possible to analyze how induced conformational changes regulate VicK catalytic activities. Therefore, this remains an interesting area for future research.

### The Low Helical Propensity of the DHp Domain

The local region around the phosphoryl receptor histidine of DHp domains is highly conserved [45]. We found that this region has a low helical propensity and is subject to significant helical bending, which is consistent with its low helical propensity. The helical bending most likely helps place His217 in close proximity to the CA domain to allow hydrogen bonds to form with the γ-phosphate of ATP (Figures 4 and 6B). Similarly, the DHp domain of *B. subtilis* DesK bends 50–54° when His188 is phosphorylated [25]. The HK853 DHp domain also bends 20° when bound to its cognate RR468 [46]. Therefore, the helical bending appears to be an intrinsic property of the DHp domain and is relevant to its function. Indeed, we showed that the combined mutations, through introducing alanines (VSP2AAA and VSTP2AAAA) into the DHp domain, abolished VicK autokinase activity (Figure 4D).

Residues Pro222 and Thr221 are conserved among VicK homologs (Figure 4C). Interestingly, these residues are essential for phosphatase activity because mutations of either P222A or T221A abolished the phosphatase activity of VicK (Figure 5). Proline, which produces a kink and 18–35° bending that affects the thermodynamic stability of the α-helix [47], plays key roles in the transmembrane signaling of several proteins, including G-protein-coupled receptors and voltage-gated potassium channels [48]. In addition to proline, threonine and serine residues are able to bend a helix 3–4° larger than alanine, which is important for the channel gating of voltage-dependent connexin32 [49],[50]. Our data in this study demonstrate that Pro222 and Thr221 are essential for phosphatase activity in VicK (Figure 4D).

Glycine may also serve a similar role as proline. The glycine in the middle of an α-helix attributes unique flexibility [51]. *B. subtilis* DesK has a glycine instead of proline present in this region [25]. Consistently, our P222G, as opposed to the P222A mutant, had phosphatase activity similar to wt VicK (Figure 5A, lane 6). It is worth noting that the DHp domain is not significantly bent in the structure of *B. subtilis* Spo0B and Spo0F complex compared with *T. maritima* HK853 and RR468 complex, although a glycine residue is localized adjacent to the phosphoryl receptor histidine [52]. However, it is possible that the crystal structure of the Spo0B and Spo0F complex captures only one state of the DHp domain of Spo0B kinase.

### Transient Formation of the Active Site

Our structure has shown that monomer *A* positions toward to its own His217 to form an active state, which is consistent with the findings using heterodimeric kinase mutants of HK853 and PhoR [46]. A flexible linker between the CA and DHp domains has been postulated to play a role in CA domain swinging [23]. In addition, the small interface created by Arg382, Phe383, and Arg385 provides a docking site as well as sufficient freedom for the CA domain to rotate (Figure S10). In the HK853 and RR468 complex, the CA domain swings ∼37° along the axis of the DHp domain when compared with free HK853 [46]. Crystal structures of *B. subtilis* DesK have shown that the CA domains could position themselves differently relative to the DHp domain [25].

The buried surface of the active monomer *A* is 1140 Å^2^. However, this interface is mainly composed of hydrophilic and polar residues, which suggests that these contacts may only be transient (Figure 6C). Our mutagenesis experiments showed that these residues are important for VicK autokinase activity (Figure 6D). Interestingly, while Ile403 mediates van der Waals contacts with Asn214 and Glu218 in the active state, it also contributes to the interface in the inactive state (Figures 6C and S10). Thr221 mediates a hydrogen bond with Asn337 and van der Waals interactions with Phe295 (Figure 6C). The T221A mutation eliminated phosphatase activity but did not affect the autokinase activity (Figures 4D and 5). In contrast, Winkler et al. found that a T221R mutant of *S. pneumoniae* VicK completely abolished its autokinase activity and greatly reduced phosphatase activity [33]. It is possible that the large arginine side chain may block the CA domain from properly accessing the DHp domain for active site formation.

It is important to note that the asymmetrical positioning of the CA domains and the different conformations of each monomer of the DHp domain are captured in a unique crystal-packing environment. The two VicK dimers form an anti-parallel tetrameric packing where the active CA position is stabilized by direct interactions with HAMP and PAS domains from another dimer in the same asymmetric unit (Figure S12). The N terminus of the HAMP domain makes contacts with the inactive CA domain of the VicK dimer from another asymmetric unit through the extended N terminus of monomer B (*N*b) (Figure 1C). However, the overall structures of the HAMP and PAS domains remain symmetric.

### Sequential Autokinase Activation Model

**Figure 7:**
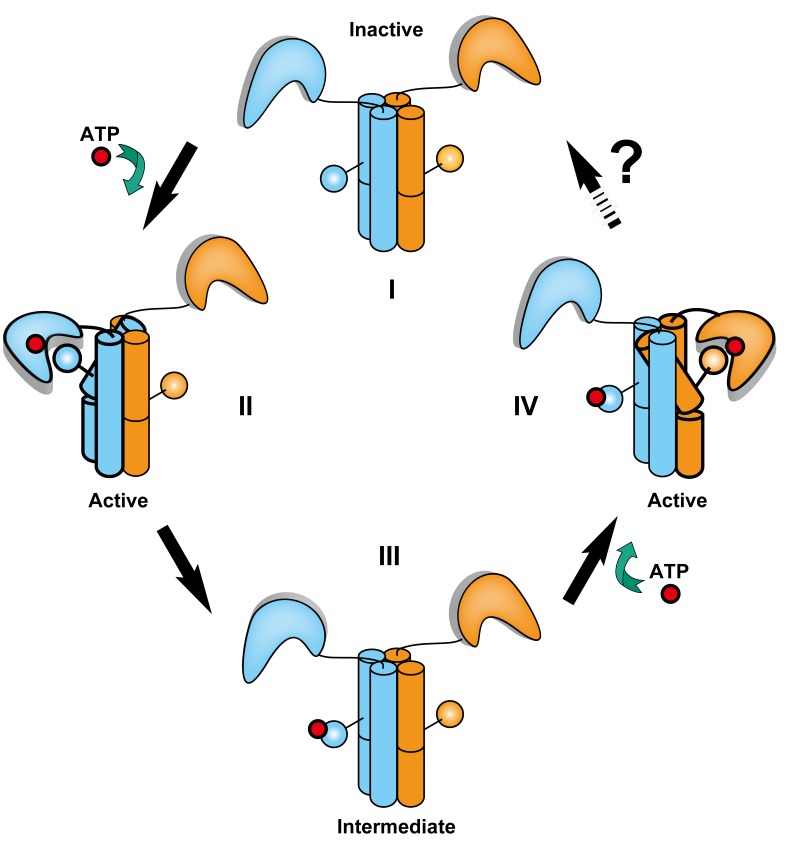
Model of VicK autokinase activation. The four cylinders in the middle of each state (I–IV) represent the four helical bundle of the DHp domain. The His217 residue is indicated in sphere and line attached to the four cylinders. The CA domains are shown in an L shape and ATP or its γ-phosphate in a red sphere. The unknown steps for VicK to return to the inactive state are indicated by a black arrow and labeled with a question mark.

Together, coordinated helical shifts of DHp and movement of the CA domains can be combined into a model to illustrate the activation steps for VicK (Figure 7). When both CA domains are inactive, they stay relaxed and further away from the phosphoryl acceptor histidine (I). Upon stimulation, one helix bends ∼25° toward the DHp central axis in coordination with the global shifts of DHp to expose the phosphoryl acceptor histidine. Consequently, one CA domain *in cis* rotates ∼61° to reach this histidine for initial phosphorylation (II). It is likely that this activation does not happen simultaneously for both CA domains because the DHp domain allows only one helix to bend at a time (Figure 4B). To fully activate both histidines (IV), VicK may go through an intermediate state that is similar to the inactive state (III) before the second CA domain rotates. Finally, the CA domains swing back to the initial state (IV to I) through several steps that remain to be determined.

## Materials and Methods

### Protein Expression and Crystallization

*S. mutans* VicK (aa31–450) was cloned into vector pET15 (Novagen). *S. mutans* VicR (aa1–235) and *S. pneumoniae* VicR (aa1–234) were cloned into pETHis vector. All these constructs were expressed with N terminal 6× Histidine tag in *Escherichia coli* BL21/DE3 Rosetta (Novagen). VicK protein was purified through nickel affinity agarose (Qiagen), Q sepharose, phenyl sepharose, and Superdex S200 (GE Healthcare). The two VicR homologs were simply purified by nickel affinity agarose and gel filtration. Protein preps were concentrated down to 8 mg/ml in a buffer of 10 mM Tris (pH 8.0), 100 mM NaCl, 300 mM ammonium acetate, 2 mM DTT, and 1 mM β-mecaptoethanol (β-ME). The preliminary crystals were obtained using the screen kits of JCSG Plus (Qiagen) and Index (Hampton Research). Most crystals from these initial screening could only diffract to ∼4.5 Å. The later optimizations of those crystals defined the best condition of 2.3–2.8 M Sodium formate, 50 mM Tris (pH 7.4–8.6), and 4% PEG 4000 at room temperature. Selenium-methionine labeled protein was prepared following a standard protocol [53].

### Structural Determination

Bar-like crystals were grown for 2–4 d and immediately frozen in liquid nitrogen after quickly soaked with crystal growth buffer and additional 12% isopropanol as a cryo-protectant. Data were collected in BM17U, Shanghai Synchrotron Radiation facility (SSRF), China and processed by HKL2000 [54] and CCP4 suite [55]. Data collection statistics are summarized in Table S2.

The selenium sites were located using SHELXD [56]. Heavy atom positions were refined and phases were calculated with PHASER [57]. The real-space constraints were applied to the electron density map in DM [58]. An initial model was then built manually using COOT [59]. The model was further refined using PHENIX with stereochemistry and secondary structure information as restraints [60]. The structure and refinement statistics are summarized in Table S2.

### Model Analyses and Graphics

Model analyses were performed using a variety of programs. The structural alignments were calculated in Coot [59]. Similar folds searches were carried out using Dali server [61]. The helical bending was calculated using a program HELANAL [62]. The buried surface areas were calculated in CNS [63]. The electrostatic potential surfaces was calculated and graphed by CCP4mg [64], while other graphics were made using Pymol (DeLano Scientific LLC).

### Multiangle Laser Light Scattering

The VicK protein prep at ∼4 mg/ml was first resolved on a size exclusion column (Shodex KW-802.5) in a buffer of 50 mM HEPES (pH 7.0) and 200 mM Na_2_SO_4_ at 25°C. Data were then collected on a DAWN HELEOS II laser photometer with an emission at 658 nm (Wyatt, USA). Molecular mass was calculated using ASTRA V (Wyatt, USA).

### Autokinase Assay by γ^32^P-ATP

All VicK mutants were generated by our modified Quikchange mutagenesis protocol [65]. All these mutants were purified as wt VicK described above. β–ME was excluded from buffer in the final protein preps. The autokinase activity of VicK was measured by using isotope γ^32^P-ATP (Perkin-Elmer, NEG002Z001MC) according to a recent protocol published by Winkler and his colleague [33]. The working solution of the hot ATP was freshly made by mixing the hot ATP with an equal volume of 3 mM cold ATP. The VicK proteins at 0.5–10 µM were incubated with a concentration gradient (0–80 µM) of the hot ATP working solution in 10 µl reaction buffer of 50 mM Tris (pH 7.5), 200 mM KCl, and 10 mM MgCl_2_ for 0.25 to 8 min at room temperature. The measurements were setup at four different time points. The protein concentration and time points for each VicK protein were pre-determined to have the signals within linear ranges. The reactions were stopped by adding 3 µl 4× SDS loading buffer. The resulted mixtures were then subjected to 12% SDS-PAGE before the gels were dried and scanned in Typhoon 9410 (GE Healthcare). Quantifications were carried out using a program Totallab Quant. The autokinase parameters were derived from non-linear regression of the Michaelis-Menten equation. For simple comparison of the various VicK mutants, the VicK protein preps of 2 µg were mixed with 0.3 µl hot ATP working solution in 15 µl reaction buffer and further incubated for 30 min at room temperature. The reactions were stopped with 4× SDS loading buffer and separated in 15% SDS-PAGE. The gels were dried before being exposed to X-ray film.

### Phosphatase Assay by PMS and HPLC

*S. pneumoniae* VicR at concentration of 4 µM was phosphorylated in 50 mM AcP, 50 mM Tris (pH 7.4), 50 mM KCl, 2 mM MgCl_2_, and 20% glycerol for 1 h at 37°C [66],[67]. The phosphorylated VicR was then mixed with VicK wt and mutants at a final concentration of 4 µM for another hour at 37°C. AcP was diluted by at least 10-fold in the latter reaction. The resulted mixtures were first analyzed by PMS [68]. Briefly, the regular 8% SDS gels (29∶1) were prepared with additional 50 µM phos-tag acrylamide (Wako) and 100 µM MnCl_2_. The gels were run at 120 V for 120 min at 4°C for the best mobility shift and stained with coomassie blue.

The reactions of phosphorylated VicR were also analyzed by HPLC [67]. Briefly, additional 20% glycerol was added into reactions of VicR after treated with AcP as described above, which were further mixed with HPLC running buffers (A: 0.1% trifluoric acid; B: 0.1% trifluoric acid and 100% acetonitrile) to reach 40% acetonitrile. HPLC was run using a reverse-phase C8 column (4.6 mm×250 mm) (Agilent 1200). The phosphorylation state of VicR was confirmed by PMS as described above.

### Sequence Conservation, Helicity, and Protein Engineering

Homologs of the full-length VicK with >50% sequence identities were initially pooled from Genebank using a program BLAST [69]. Redundant entries of 96%–100% identity were identified using a multiple alignment program CLUSTAL [70] and subsequently removed, resulting in >50 unique homologs with 50%–96% identities (Figure S11). The DHp regions corresponding to amino acids 197–269 of these VicK homologs were used to generate the conservation logo using Weblogo server (weblogo.berkeley.edu). Redundant sequences (with 100% identity) of the DHp domain were further removed, resulting in >40 unique sequences (Figure S6). The helicity of the DHp was analyzed by a comprehensive secondary structure prediction program Phyre, which scores each amino acid as 0–9 for a helical probability [71]. DHp engineering was carried out through reiterative process of mutations and secondary structure calculation. Those unfavorable amino acids to α-helices within the bending region of DHp domain were determined according to statistical analyses [39].

### Accession Number

The coordinates of the structure and relevant information have been deposited into the Protein Data Bank (4I5S).

## Supporting Information

Figure S1 **Structural analyses of VicK HAMP domain.** (A) Ribbon presentation of VicK HAMP domain. The right is the top view, 90° rotated along a horizontal axis of the dashed line. The colors and labels are the same as those in Figure 1C. The connecting loop between helices α1 and α2 is labeled as L1. (B) Interaction networks between helices of VicK HAMP. The Cα backbones are represented in puffy ribbon and colored by B factors. Hydrophilic and polar residues are shown in gold sticks. Residues involved in knobs-into-holes packing are in green sticks while those in the central hydrophobic core are in blue sticks. Salt bridges and hydrogen bonds are indicated in yellow dashed lines. The two red circles separate their interactions into three layers (outer shell, middle shell, and inner shell). (C) The electrostatic potential surface of the HAMP domain. The color scheme is the same as that of Figure 1D. Three rings of hydrogen bond networks in outer shell are indicated with yellow arrows.
(JPG)

Figure S2 **The canonical PAS domain and its interaction network with leucine zipper.** (A) Alignment of two canonical PAS domains of VicK. The color scheme is the same as that of Figure 1C. (B) The interaction network between the leucine-zipper and the canonical PAS domain. This figure depicts only one side of their interaction network mediated by the leucine-zipper helix of monomer A (magenta ribbon) and the β-sheet of the PAS domain of monomer B (gold thick line). Residues involved in hydrogen bonds and hydrophobic interactions are labeled with sticks and these contacts are further shown in blue dashed lines.
(JPG)

Figure S3 **Sequence alignment of ligand-bound PAS domains with that of VicK.** All representative ligand-bound PAS domains were selected according to a recent review by Henry and Crosson [11]. The alignment was performed and color-boxed by PRALINE [72]. FixL and DosH are two heme binding PAS domains [73],[74]. NifL is a FAD binding PAS domain [75]. DcuS, CitA, and DctB are the di- and tri-carboxylate binding PAS domains [76]–[78], and PhoQ is a Ca2+ binding PAS domain [79].
(TIFF)

Figure S4 **Structural alignments of ligand-bound PAS domains with** ***S. mutans*** **VicK.** The alignments were performed by Coot [59]. Rmsd of the aligned Cα backbone is indicated below each alignment and the total numbers of amino acids used in the alignment are shown in parentheses. (A) Alignment of the VicK PAS domain with *Azotobacter vinelandii* NifL (2GJ3, shown in cyan) [75]. FAD from NifL is represented with sticks. (B) Alignment of the VicK PAS domain with *Bradyrhizobium japonicum* FixL (1DRM, shown in green) [73]. Heme from FixL is represented with sticks. (D) Alignment of the VicK PAS domain with *E. coli* DcuS (3BY8, shown in blue) [76]. Malate ion from DcuS is represented with sticks. (D) Alignment of the VicK PAS domain with *Salmonella typhimurium* PhoQ (1YAX, shown in grey) [79]. Calcium ion from PhoQ is represented with a sphere.
(JPG)

Figure S5 **Structure of the VicK C terminal DHp/CA domain.** (A) C terminal domains of VicK shown in ribbon. The color scheme is the same as that described for Figure 1C. Two disordered loops L7 and 11 are labeled in dashed lines. The phosphoryl acceptor His217 is shown in green sticks. (B) CA domains of VicK with the conserved globular folds. The blue arrow indicates a distinct conformational region between two CA domains. (C) The CA domain of *T. maritima* HK853 aligned with both VicK CA domains. HK853 CA domain is colored in grey and VicK CA domains in magenta and gold. ADP is taken from the HK853 structure.
(JPG)

Figure S6 **Alignment of the DHp domain of the non-redundant VicK homologs.** The alignment is colored by default in CLUSTAL program [70]. The amino acids are grouped into hydrophobic (red), polar (green), basic (blue), and acidic (pink) residues. Highly conserved residues are labeled by asterisks. Similar residues are labeled with colons and less conserved residues are with periods. S, *Streptococcus*; E, *Enterococcus*; L, *Lactobacillus*; Lc, *Lactococcus*; P, *Pediococcus*. The alignment of all full-length homologs is presented in Figure S11.
(JPG)

Figure S7 **Mutations in DHp domain did not affect the interaction between VicK and VicR.** (A,B) The interactions of VicK and VicR were detected by native PAGE. All buffers were essentially the same as regular SDS-PAGE but without SDS. The 10% gels were used and run under 120 V for 120 min at 4°C and stained with coomassie blue. VicR, VicK, and the VicK/VicR complex are indicated by black, green, and red arrows, respectively. The wt VicK and mutants are labeled on top of each gel.
(JPG)

Figure S8 **Alignment of the C terminal VicK with HK853.** The alignment was performed as described in Figure 4B. The monomers of the VicK are colored in magenta and gold and the monomers of *T. maritima* HK853 in blue and cyan. The red circle is an axis in parallel to the DHp domain and going to the paper. The black dashed line is an axis perpendicular to the DHp domain. Grey arrows indicate rotation directions and rotation angles are labeled. His217 is highlighted in green sticks.
(JPG)

Figure S9 **Interface between the DHp and active CA domain.** The CA domain is presented in an electrostatic potential surface as described in Figure 1D. The two helices of DHp are in spectrally colored ribbon. The green star indicates His217 position in close proximity to the ATP binding pocket of the CA domain.
(JPG)

Figure S10 **Detailed interactions between the VicK DHp domain and inactive CA domain.** Residues involved in hydrogen bonds and hydrophobic interactions are labeled in sticks. Hydrogen bonds are shown in yellow dashed lines.
(JPG)

Figure S11 **Alignment of** *S. mutans* **VicK homologs.** The alignment are colored by default in CLUSTAL program [70]. The amino acids are grouped into hydrophobic (red), polar (green), basic (blue), and acidic (pink) residues. Highly conserved residues are labeled by asterisks on the bottom of the alignment. Similar residues are labeled with colons and less conserved residues are with periods. The positions for H, N, G1, F, G2, and G3 boxes are labeled on the top of the alignment. The abbreviations of genera are described in Figure S6.
(PDF)

Figure S12 **Crystal contacts of two VicK dimers in one asymmetric unit.** The top dimer is in ribbon colored and labeled as described in Figure 1C. The HAMP, PAS, and DHp/CA domains of the bottom dimer are shown in molecular surfaces and colored in steelblue, skyblue, and green, respectively. Loop L4 from the bottom dimer is presented in molecular surface and labeled in white. Loops L4 and L5 from the top dimer are presented in ribbon and labeled in blue.
(JPG)

Table S1 **Autokinase activity of VicK wt and mutants.** *The measurement performed at a protein concentration of 0.5 µM with time points of 15 s, 30 s, 45 s, and 60 s. **Not able to determine. ***The measurement performed at a protein concentration of 1 µM with time points of 15 s, 30 s, 45 s, and 60 s. #The measurement performed at a protein concentration of 10 µM with time points of 60 s, 120 s, 240 s, and 480 s.
(XLS)

Table S2 **Statistics of data collection and structure refinement.** ^†^The data for the highest resolution shell are shown in parenthesis. *Rsym = ∑|I−<I>|/∑I, where I is the observed intensity, <I> is the statistically weighted average intensity of multiple observations of symmetry-related reflections. ^††^I/σ(I) – ratio of mean intensity to a mean standard deviation of intensity. ^‡^R = ∑||Fo|−|Fc||/∑|Fo|, where Fo and Fc are observed and calculated structure factor amplitudes, respectively. **R_free_ is calculated using 5% of randomly selected reflections. ***Number of protein atoms of the ordered regions. ^§^Rmsd – root mean square deviation.
(DOC)

## Abbreviations

AcP: acetyl phosphate
CA: catalytic and ATP binding domain
DHp: dimerization and histidine phosphorylation
FAD: flavin adenine dinucleotide
HAMP: histidine kinase, adenyl cyclase, methyl-accepting proteins, phosphatase
HPLC: high performance liquid chromatography
PAS: Per-ARNT-SIM
rmsd: root mean square deviation
PMS: Phos-tag gel mobility shift
RR: response regulator
SK: sensor kinase
TCS: two-component system
TM: transmembrane domain
wt: wild-type

## References

[1] Nixon BT, Ronson CW, Ausubel FM (1986) Two-component regulatory systems responsive to environmental stimuli share strongly conserved domains with the nitrogen assimilation regulatory genes ntrB and ntrC. Proc Natl Acad Sci U S A 83: 7850–7854. 3020561 10.1073/pnas.83.20.7850 PMC386820

[2] Stock AM, Robinson VL, Goudreau PN (2000) Two-component signal transduction. Annu Rev Biochem 69: 183–215. 10966457 10.1146/annurev.biochem.69.1.183

[3] Stephenson K, Hoch JA (2002) Two-component and phosphorelay signal-transduction systems as therapeutic targets. Curr Opin Pharmacol 2: 507–512. 12324251 10.1016/s1471-4892(02)00194-7

[4] Gotoh Y, Eguchi Y, Watanabe T, Okamoto S, Doi A, et al (2010) Two-component signal transduction as potential drug targets in pathogenic bacteria. Curr Opin Microbiol 13: 232–239. 20138000 10.1016/j.mib.2010.01.008

[5] Zhulin IB, Taylor BL, Dixon R (1997) PAS domain S-boxes in Archaea, bacteria and sensors for oxygen and redox. Trends Biochem Sci 22: 331–333. 9301332 10.1016/s0968-0004(97)01110-9

[6] Aravind L, Ponting CP (1999) The cytoplasmic helical linker domain of receptor histidine kinase and methyl-accepting proteins is common to many prokaryotic signalling proteins. FEMS Microbiol Lett 176: 111–116. 10418137 10.1111/j.1574-6968.1999.tb13650.x

[7] Galperin MY, Nikolskaya AN, Koonin EV (2001) Novel domains of the prokaryotic two-component signal transduction systems. FEMS Microbiol Lett 203: 11–21. 11557134 10.1111/j.1574-6968.2001.tb10814.x

[8] Ponting CP, Aravind L (1997) PAS: a multifunctional domain family comes to light. Curr Biol 7: R674–677. 9382818 10.1016/s0960-9822(06)00352-6

[9] Taylor BL, Zhulin IB (1999) PAS domains: internal sensors of oxygen, redox potential, and light. Microbiol Mol Biol Rev 63: 479–506. 10357859 10.1128/mmbr.63.2.479-506.1999 PMC98974

[10] Moglich A, Ayers RA, Moffat K (2009) Structure and signaling mechanism of Per-ARNT-Sim domains. Structure 17: 1282–1294. 19836329 10.1016/j.str.2009.08.011 PMC3092527

[11] Henry JT, Crosson S (2011) Ligand-binding PAS domains in a genomic, cellular, and structural context. Annu Rev Microbiol 65: 261–286. 21663441 10.1146/annurev-micro-121809-151631 PMC3298442

[12] Slavny P, Little R, Salinas P, Clarke TA, Dixon R (2010) Quaternary structure changes in a second Per-Arnt-Sim domain mediate intramolecular redox signal relay in the NifL regulatory protein. Mol Microbiol 75: 61–75. 19906177 10.1111/j.1365-2958.2009.06956.x

[13] Parkinson JS (2010) Signaling mechanisms of HAMP domains in chemoreceptors and sensor kinases. Annu Rev Microbiol 64: 101–122. 20690824 10.1146/annurev.micro.112408.134215

[14] Hulko M, Berndt F, Gruber M, Linder JU, Truffault V, et al (2006) The HAMP domain structure implies helix rotation in transmembrane signaling. Cell 126: 929–940. 16959572 10.1016/j.cell.2006.06.058

[15] Dunin-Horkawicz S, Lupas AN (2010) Comprehensive analysis of HAMP domains: implications for transmembrane signal transduction. J Mol Biol 397: 1156–1174. 20184894 10.1016/j.jmb.2010.02.031

[16] Ferris HU, Dunin-Horkawicz S, Mondejar LG, Hulko M, Hantke K, et al (2011) The mechanisms of HAMP-mediated signaling in transmembrane receptors. Structure 19: 378–385. 21397188 10.1016/j.str.2011.01.006

[17] Manson MD (2008) The tie that binds the dynamic duo: the connector between AS1 and AS2 in the HAMP domain of the *Escherichia coli* Tsr chemoreceptor. J Bacteriol 190: 6544–6547. 18708501 10.1128/JB.00943-08 PMC2566210

[18] Watts KJ, Johnson MS, Taylor BL (2008) Structure-function relationships in the HAMP and proximal signaling domains of the aerotaxis receptor Aer. J Bacteriol 190: 2118–2127. 18203838 10.1128/JB.01858-07 PMC2258896

[19] Zhou Q, Ames P, Parkinson JS (2009) Mutational analyses of HAMP helices suggest a dynamic bundle model of input-output signalling in chemoreceptors. Mol Microbiol 73: 801–814. 19656294 10.1111/j.1365-2958.2009.06819.x PMC2749569

[20] Airola MV, Watts KJ, Bilwes AM, Crane BR (2010) Structure of concatenated HAMP domains provides a mechanism for signal transduction. Structure 18: 436–448. 20399181 10.1016/j.str.2010.01.013 PMC2892831

[21] Zhou Q, Ames P, Parkinson JS (2011) Biphasic control logic of HAMP domain signalling in the *Escherichia coli* serine chemoreceptor. Mol Microbiol 80: 596–611. 21306449 10.1111/j.1365-2958.2011.07577.x PMC3095108

[22] Watts KJ, Johnson MS, Taylor BL (2011) Different conformations of the kinase-on and kinase-off signaling states in the Aer HAMP domain. J Bacteriol 193: 4095–4103. 21665965 10.1128/JB.01069-10 PMC3147692

[23] Marina A, Waldburger CD, Hendrickson WA (2005) Structure of the entire cytoplasmic portion of a sensor histidine-kinase protein. EMBO J 24: 4247–4259. 16319927 10.1038/sj.emboj.7600886 PMC1356327

[24] Tomomori C, Tanaka T, Dutta R, Park H, Saha SK, et al (1999) Solution structure of the homodimeric core domain of *Escherichia coli* histidine kinase EnvZ. Nat Struct Biol 6: 729–734. 10426948 10.1038/11495

[25] Albanesi D, Martin M, Trajtenberg F, Mansilla MC, Haouz A, et al (2009) Structural plasticity and catalysis regulation of a thermosensor histidine kinase. Proc Natl Acad Sci U S A 106: 16185–16190. 19805278 10.1073/pnas.0906699106 PMC2738621

[26] Dubrac S, Bisicchia P, Devine KM, Msadek T (2008) A matter of life and death: cell wall homeostasis and the WalKR (YycGF) essential signal transduction pathway. Mol Microbiol 70: 1307–1322. 19019149 10.1111/j.1365-2958.2008.06483.x

[27] Dubrac S, Boneca IG, Poupel O, Msadek T (2007) New insights into the WalK/WalR (YycG/YycF) essential signal transduction pathway reveal a major role in controlling cell wall metabolism and biofilm formation in *Staphylococcus aureus* . J Bacteriol 189: 8257–8269. 17827301 10.1128/JB.00645-07 PMC2168699

[28] Ajdic D, McShan WM, McLaughlin RE, Savic G, Chang J, et al (2002) Genome sequence of *Streptococcus mutans* UA159, a cariogenic dental pathogen. Proc Natl Acad Sci U S A 99: 14434–14439. 12397186 10.1073/pnas.172501299 PMC137901

[29] Lee SF, Delaney GD, Elkhateeb M (2004) A two-component covRS regulatory system regulates expression of fructosyltransferase and a novel extracellular carbohydrate in *Streptococcus mutans* . Infect Immun 72: 3968–3973. 15213141 10.1128/IAI.72.7.3968-3973.2004 PMC427443

[30] Senadheera MD, Guggenheim B, Spatafora GA, Huang YC, Choi J, et al (2005) A VicRK signal transduction system in *Streptococcus mutans* affects gtfBCD, gbpB, and ftf expression, biofilm formation, and genetic competence development. J Bacteriol 187: 4064–4076. 15937169 10.1128/JB.187.12.4064-4076.2005 PMC1151735

[31] Senadheera D, Krastel K, Mair R, Persadmehr A, Abranches J, et al (2009) Inactivation of VicK affects acid production and acid survival of *Streptococcus mutans* . J Bacteriol 191: 6415–6424. 19684142 10.1128/JB.00793-09 PMC2753040

[32] Kim D, Forst S (2001) Genomic analysis of the histidine kinase family in bacteria and archaea. Microbiology 147: 1197–1212. 11320123 10.1099/00221287-147-5-1197

[33] Gutu AD, Wayne KJ, Sham LT, Winkler ME (2010) Kinetic characterization of the WalRKSpn (VicRK) two-component system of *Streptococcus pneumoniae*: dependence of WalKSpn (VicK) phosphatase activity on its PAS domain. J Bacteriol 192: 2346–2358. 20190050 10.1128/JB.01690-09 PMC2863487

[34] Szurmant H, White RA, Hoch JA (2007) Sensor complexes regulating two-component signal transduction. Curr Opin Struct Biol 17: 706–715. 17913492 10.1016/j.sbi.2007.08.019 PMC2175030

[35] Cheung J, Hendrickson WA (2010) Sensor domains of two-component regulatory systems. Curr Opin Microbiol 13: 116–123. 20223701 10.1016/j.mib.2010.01.016 PMC3078554

[36] Krell T, Lacal J, Busch A, Silva-Jimenez H, Guazzaroni ME, et al (2010) Bacterial sensor kinases: diversity in the recognition of environmental signals. Annu Rev Microbiol 64: 539–559. 20825354 10.1146/annurev.micro.112408.134054

[37] Crick FHC (1953) The packing of a-helices: simple coiled-coils. Acta Crystallogr 6: 689–697.

[38] Echenique JR, Trombe MC (2001) Competence repression under oxygen limitation through the two-component MicAB signal-transducing system in *Streptococcus pneumoniae* and involvement of the PAS domain of MicB. J Bacteriol 183: 4599–4608. 11443095 10.1128/JB.183.15.4599-4608.2001 PMC95355

[39] Williams RW, Chang A, Juretic D, Loughran S (1987) Secondary structure predictions and medium range interactions. Biochim Biophys Acta 916: 200–204. 3676331 10.1016/0167-4838(87)90109-9

[40] Celikel R, Veldore VH, Mathews I, Devine KM, Varughese KI (2012) ATP forms a stable complex with the essential histidine kinase WalK (YycG) domain. Acta Crystallogr D Biol Crystallogr 68: 839–845. 22751669 10.1107/S090744491201373X PMC3388812

[41] Fukushima T, Szurmant H, Kim EJ, Perego M, Hoch JA (2008) A sensor histidine kinase co-ordinates cell wall architecture with cell division in *Bacillus subtilis* . Mol Microbiol 69: 621–632. 18573169 10.1111/j.1365-2958.2008.06308.x PMC2574549

[42] Wayne KJ, Sham LT, Tsui HC, Gutu AD, Barendt SM, et al (2010) Localization and cellular amounts of the WalRKJ (VicRKX) two-component regulatory system proteins in serotype 2 *Streptococcus pneumoniae* . J Bacteriol 192: 4388–4394. 20622066 10.1128/JB.00578-10 PMC2937396

[43] Kishii R, Falzon L, Yoshida T, Kobayashi H, Inouye M (2007) Structural and functional studies of the HAMP domain of EnvZ, an osmosensing transmembrane histidine kinase in *Escherichia coli* . J Biol Chem 282: 26401–26408. 17635923 10.1074/jbc.M701342200

[44] Yamada S, Sugimoto H, Kobayashi M, Ohno A, Nakamura H, et al (2009) Structure of PAS-linked histidine kinase and the response regulator complex. Structure 17: 1333–1344. 19836334 10.1016/j.str.2009.07.016

[45] Huynh TN, Noriega CE, Stewart V (2010) Conserved mechanism for sensor phosphatase control of two-component signaling revealed in the nitrate sensor NarX. Proc Natl Acad Sci U S A 107: 21140–21145. 21078995 10.1073/pnas.1013081107 PMC3000247

[46] Casino P, Rubio V, Marina A (2009) Structural insight into partner specificity and phosphoryl transfer in two-component signal transduction. Cell 139: 325–336. 19800110 10.1016/j.cell.2009.08.032

[47] Yun RH, Anderson A, Hermans J (1991) Proline in alpha-helix: stability and conformation studied by dynamics simulation. Proteins 10: 219–228. 1881878 10.1002/prot.340100306

[48] Sansom MS, Weinstein H (2000) Hinges, swivels and switches: the role of prolines in signalling via transmembrane alpha-helices. Trends Pharmacol Sci 21: 445–451. 11121576 10.1016/s0165-6147(00)01553-4

[49] Ballesteros JA, Deupi X, Olivella M, Haaksma EE, Pardo L (2000) Serine and threonine residues bend alpha-helices in the chi(1) = g(-) conformation. Biophys J 79: 2754–2760. 11053148 10.1016/S0006-3495(00)76514-3 PMC1301156

[50] Ri Y, Ballesteros JA, Abrams CK, Oh S, Verselis VK, et al (1999) The role of a conserved proline residue in mediating conformational changes associated with voltage gating of Cx32 gap junctions. Biophys J 76: 2887–2898. 10354417 10.1016/S0006-3495(99)77444-8 PMC1300261

[51] Blaber M, Zhang XJ, Matthews BW (1993) Structural basis of amino acid alpha helix propensity. Science 260: 1637–1640. 8503008 10.1126/science.8503008

[52] Zapf J, Sen U, Madhusudan, Hoch JA, Varughese KI (2000) A transient interaction between two phosphorelay proteins trapped in a crystal lattice reveals the mechanism of molecular recognition and phosphotransfer in signal transduction. Structure 8: 851–862. 10997904 10.1016/s0969-2126(00)00174-x

[53] Doublie S (1997) Preparation of selenomethionyl proteins for phase determination. Methods Enzymol 276: 523–530. 9048379

[54] Otwinowski Z, Minor W (1997) Processing of X-ray diffraction data collected in oscillation mode. Method Enzymol 276: 307–326. 10.1016/S0076-6879(97)76066-X 27754618

[55] ccp4 (1994) The CCP4 suite: programs for protein crystallography. Acta Crystallogr D Biol Crystallogr 50: 760–763. 15299374 10.1107/S0907444994003112

[56] Schneider TR, Sheldrick GM (2002) Substructure solution with SHELXD. Acta Crystallogr D Biol Crystallogr 58: 1772–1779. 12351820 10.1107/s0907444902011678

[57] McCoy AJ, Grosse-Kunstleve RW, Adams PD, Winn MD, Storoni LC, et al (2007) Phaser crystallographic software. J Appl Crystallogr 40: 658–674. 19461840 10.1107/S0021889807021206 PMC2483472

[58] Cowtan K (1994) ‘dm’: An automated procedure for phase improvement by density modification. Joint CCP4 and ESF-EACBM Newsletter on Protein Crystallography 31: 34–38.

[59] Emsley P, Cowtan K (2004) Coot: model-building tools for molecular graphics. Acta Crystallogr D Biol Crystallogr 60: 2126–2132. 15572765 10.1107/S0907444904019158

[60] Adams PD, Grosse-Kunstleve RW, Hung LW, Ioerger TR, McCoy AJ, et al (2002) PHENIX: building new software for automated crystallographic structure determination. Acta Crystallogr D Biol Crystallogr 58: 1948–1954. 12393927 10.1107/s0907444902016657

[61] Holm L, Rosenstrom P (2010) Dali server: conservation mapping in 3D. Nucleic Acids Res 38 Suppl: W545–549. 20457744 10.1093/nar/gkq366 PMC2896194

[62] Bansal M, Kumar S, Velavan R (2000) HELANAL: a program to characterize helix geometry in proteins. J Biomol Struct Dyn 17: 811–819. 10798526 10.1080/07391102.2000.10506570

[63] Brunger AT, Adams PD, Clore GM, DeLano WL, Gros P, et al (1998) Crystallography & NMR system: A new software suite for macromolecular structure determination. Acta Crystallogr D Biol Crystallogr 54: 905–921. 9757107 10.1107/s0907444998003254

[64] Potterton E, McNicholas S, Krissinel E, Cowtan K, Noble M (2002) The CCP4 molecular-graphics project. Acta Crystallogr D Biol Crystallogr 58: 1955–1957. 12393928 10.1107/s0907444902015391

[65] Mao Y, Lin J, Zhou A, Ji K, Downey JS, et al (2011) Quikgene: a gene synthesis method integrated with ligation-free cloning. Anal Biochem 415: 21–26. 21530481 10.1016/j.ab.2011.04.004

[66] Kenney LJ, Bauer MD, Silhavy TJ (1995) Phosphorylation-dependent conformational changes in OmpR, an osmoregulatory DNA-binding protein of *Escherichia coli* . Proc Natl Acad Sci U S A 92: 8866–8870. 7568033 10.1073/pnas.92.19.8866 PMC41068

[67] Ng WL, Tsui HC, Winkler ME (2005) Regulation of the pspA virulence factor and essential pcsB murein biosynthetic genes by the phosphorylated VicR (YycF) response regulator in *Streptococcus pneumoniae* . J Bacteriol 187: 7444–7459. 16237028 10.1128/JB.187.21.7444-7459.2005 PMC1272996

[68] Barbieri CM, Stock AM (2008) Universally applicable methods for monitoring response regulator aspartate phosphorylation both in vitro and in vivo using Phos-tag-based reagents. Anal Biochem 376: 73–82. 18328252 10.1016/j.ab.2008.02.004 PMC2504525

[69] Altschul SF, Gish W, Miller W, Myers EW, Lipman DJ (1990) Basic local alignment search tool. J Mol Biol 215: 403–410. 2231712 10.1016/S0022-2836(05)80360-2

[70] Larkin MA, Blackshields G, Brown NP, Chenna R, McGettigan PA, et al (2007) Clustal W and Clustal X version 2.0. Bioinformatics 23: 2947–2948. 17846036 10.1093/bioinformatics/btm404

[71] Kelley LA, Sternberg MJ (2009) Protein structure prediction on the Web: a case study using the Phyre server. Nat Protoc 4: 363–371. 19247286 10.1038/nprot.2009.2

[72] Simossis VA, Heringa J (2005) PRALINE: a multiple sequence alignment toolbox that integrates homology-extended and secondary structure information. Nucleic Acids Res 33: W289–294. 15980472 10.1093/nar/gki390 PMC1160151

[73] Gong W, Hao B, Mansy SS, Gonzalez G, Gilles-Gonzalez MA, et al (1998) Structure of a biological oxygen sensor: a new mechanism for heme-driven signal transduction. Proc Natl Acad Sci U S A 95: 15177–15182. 9860942 10.1073/pnas.95.26.15177 PMC28016

[74] Park H, Suquet C, Satterlee JD, Kang C (2004) Insights into signal transduction involving PAS domain oxygen-sensing heme proteins from the X-ray crystal structure of *Escherichia coli* Dos heme domain (Ec DosH). Biochemistry 43: 2738–2746. 15005609 10.1021/bi035980p

[75] Key J, Hefti M, Purcell EB, Moffat K (2007) Structure of the redox sensor domain of Azotobacter vinelandii NifL at atomic resolution: signaling, dimerization, and mechanism. Biochemistry 46: 3614–3623. 17319691 10.1021/bi0620407

[76] Cheung J, Hendrickson WA (2008) Crystal structures of C4-dicarboxylate ligand complexes with sensor domains of histidine kinases DcuS and DctB. J Biol Chem 283: 30256–30265. 18701447 10.1074/jbc.M805253200 PMC2573060

[77] Reinelt S, Hofmann E, Gerharz T, Bott M, Madden DR (2003) The structure of the periplasmic ligand-binding domain of the sensor kinase CitA reveals the first extracellular PAS domain. J Biol Chem 278: 39189–39196. 12867417 10.1074/jbc.M305864200

[78] Zhou YF, Nan B, Nan J, Ma Q, Panjikar S, et al (2008) C4-dicarboxylates sensing mechanism revealed by the crystal structures of DctB sensor domain. J Mol Biol 383: 49–61. 18725229 10.1016/j.jmb.2008.08.010

[79] Cho US, Bader MW, Amaya MF, Daley ME, Klevit RE, et al (2006) Metal bridges between the PhoQ sensor domain and the membrane regulate transmembrane signaling. J Mol Biol 356: 1193–1206. 16406409 10.1016/j.jmb.2005.12.032

