# Supplementary figures and images for: Mechanistic Insights Revealed by the Crystal Structure of a Histidine Kinase with Signal Transducer and Sensor Domains
Source: PLoS Biol. 2013 Feb 26;11(2):e1001493. doi: 10.1371/journal.pbio.1001493 (PMC3582566; doi:10.1371/journal.pbio.1001493)

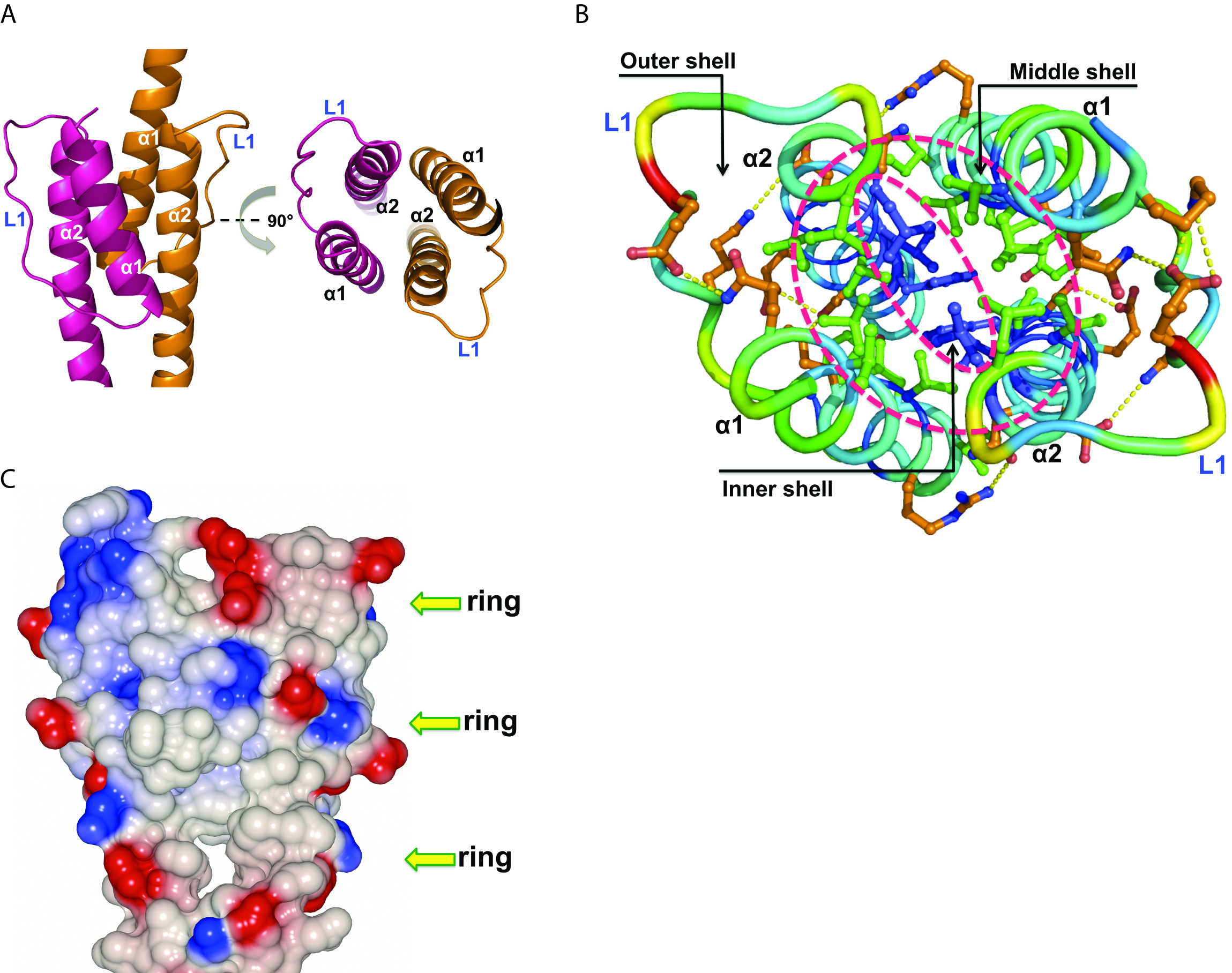

Supplement: Figure S1 — Structural analyses of VicK HAMP domain. (A) Ribbon presentation of VicK HAMP domain. The right is the top view, 90° rotated along a horizontal axis of the dashed line. The colors and labels are the same as those in Figure 1C. The connecting loop between helices α1 and α2 is labeled as L1. (B) Interaction networks between helices of VicK HAMP. The Cα backbones are represented in puffy ribbon and colored by B factors. Hydrophilic and polar residues are shown in gold sticks. Residues involved in knobs-into-holes packing are in green sticks while those in the central hydrophobic core are in blue sticks. Salt bridges and hydrogen bonds are indicated in yellow dashed lines. The two red circles separate their interactions into three layers (outer shell, middle shell, and inner shell). (C) The electrostatic potential surface of the HAMP domain. The color scheme is the same as that of Figure 1D. Three rings of hydrogen bond networks in outer shell are indicated with yellow arrows. (JPG) [file pbio.1001493.s001.jpg]

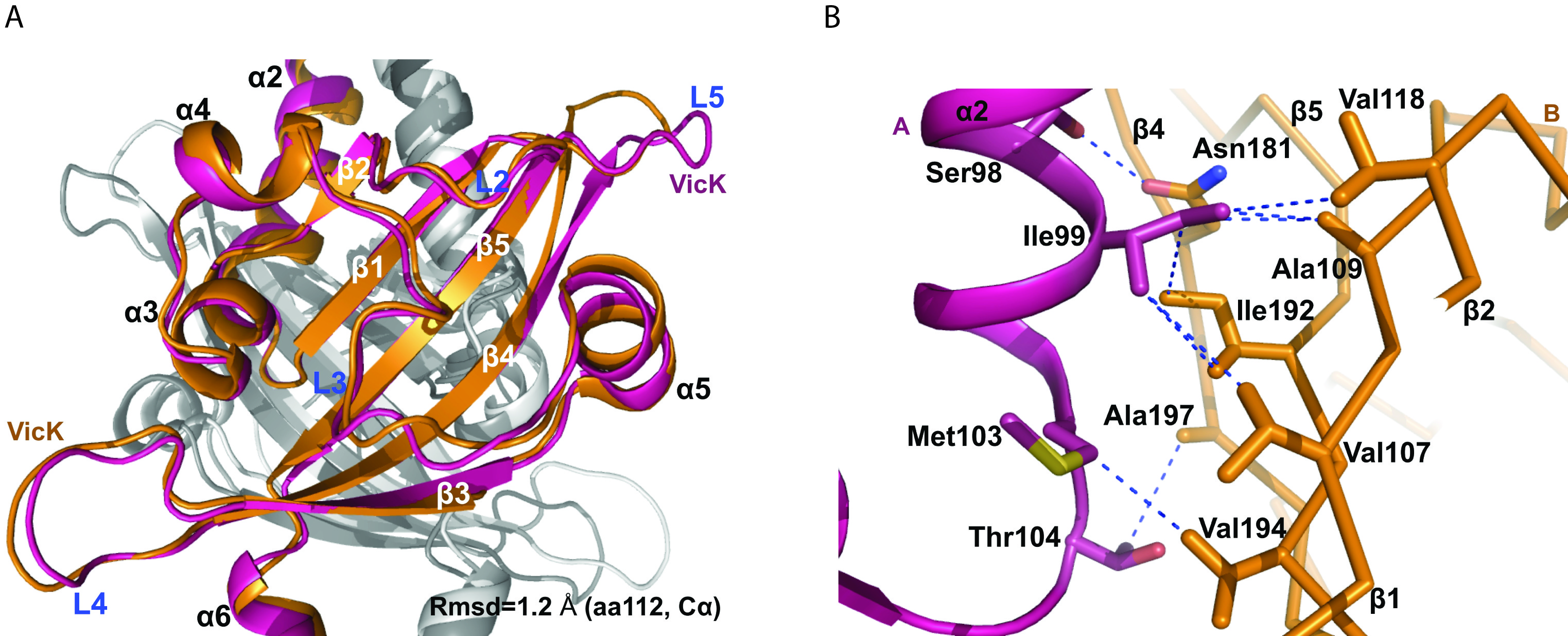

Supplement: Figure S2 — The canonical PAS domain and its interaction network with leucine zipper. (A) Alignment of two canonical PAS domains of VicK. The color scheme is the same as that of Figure 1C. (B) The interaction network between the leucine-zipper and the canonical PAS domain. This figure depicts only one side of their interaction network mediated by the leucine-zipper helix of monomer A (magenta ribbon) and the β-sheet of the PAS domain of monomer B (gold thick line). Residues involved in hydrogen bonds and hydrophobic interactions are labeled with sticks and these contacts are further shown in blue dashed lines. (JPG) [file pbio.1001493.s002.jpg]

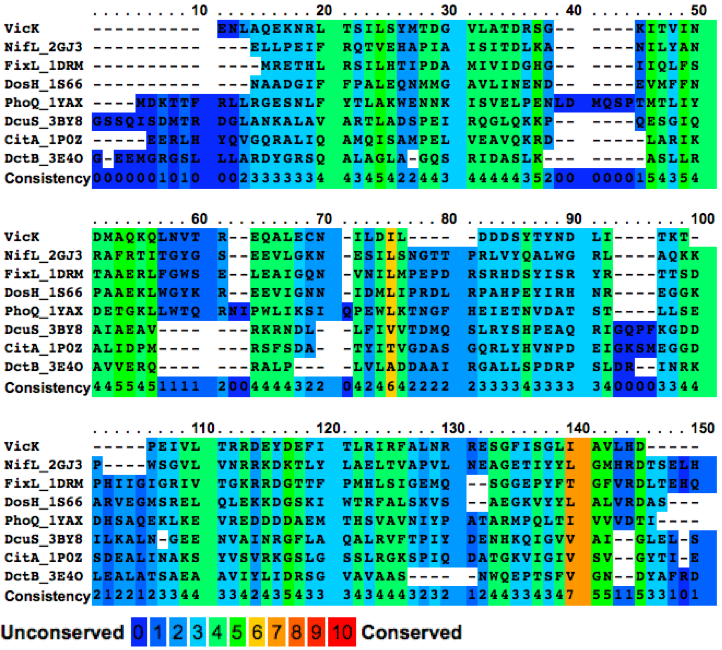

Supplement: Figure S3 — Sequence alignment of ligand-bound PAS domains with that of VicK. All representative ligand-bound PAS domains were selected according to a recent review by Henry and Crosson [11]. The alignment was performed and color-boxed by PRALINE [72]. FixL and DosH are two heme binding PAS domains [73],[74]. NifL is a FAD binding PAS domain [75]. DcuS, CitA, and DctB are the di- and tri-carboxylate binding PAS domains [76]–[78], and PhoQ is a Ca2+ binding PAS domain [79]. (TIFF) [file pbio.1001493.s003.tiff]

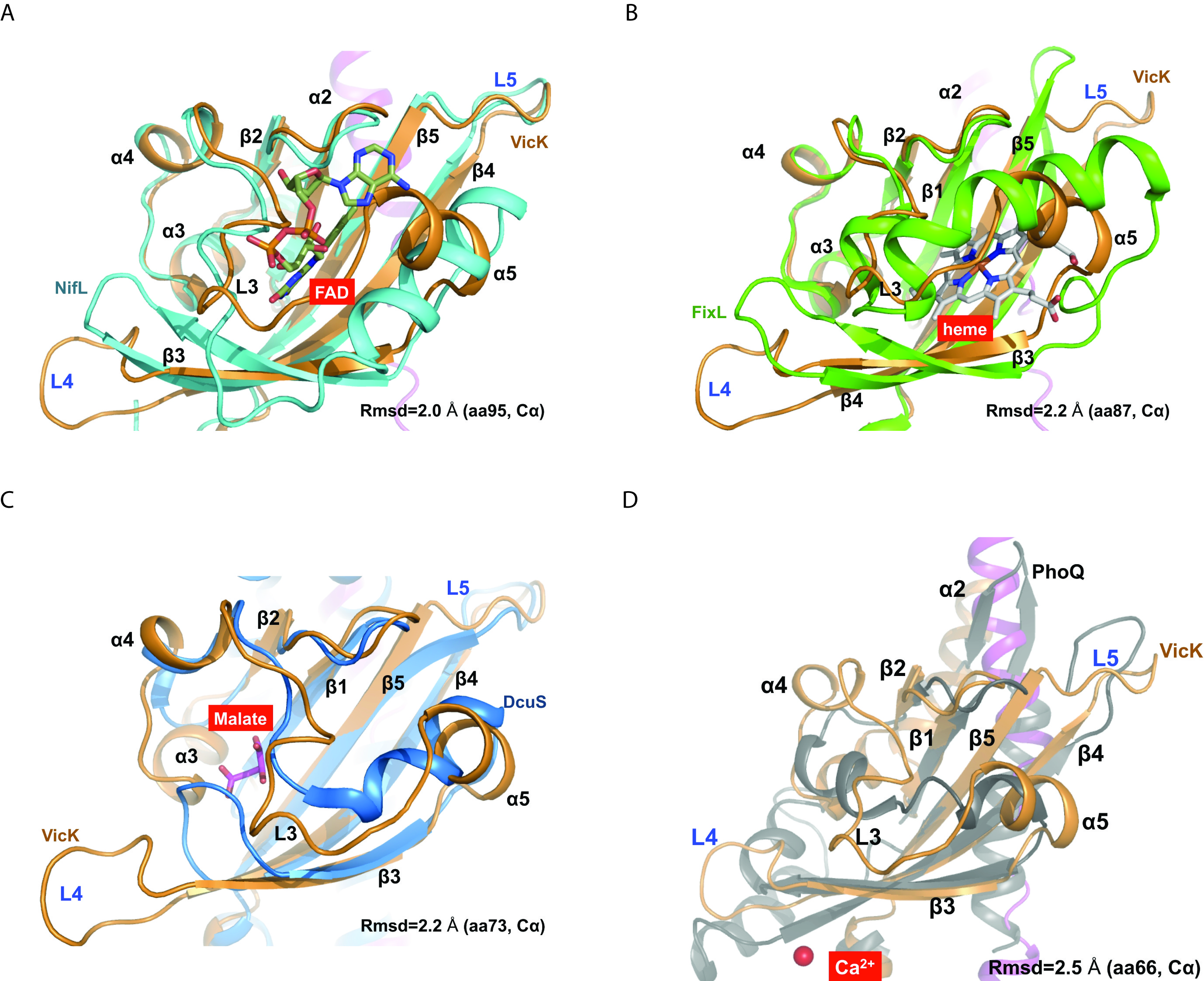

Supplement: Figure S4 — Structural alignments of ligand-bound PAS domains with S. mutans VicK. The alignments were performed by Coot [59]. Rmsd of the aligned Cα backbone is indicated below each alignment and the total numbers of amino acids used in the alignment are shown in parentheses. (A) Alignment of the VicK PAS domain with Azotobacter vinelandii NifL (2GJ3, shown in cyan) [75]. FAD from NifL is represented with sticks. (B) Alignment of the VicK PAS domain with Bradyrhizobium japonicum FixL (1DRM, shown in green) [73]. Heme from FixL is represented with sticks. (D) Alignment of the VicK PAS domain with E. coli DcuS (3BY8, shown in blue) [76]. Malate ion from DcuS is represented with sticks. (D) Alignment of the VicK PAS domain with Salmonella typhimurium PhoQ (1YAX, shown in grey) [79]. Calcium ion from PhoQ is represented with a sphere. (JPG) [file pbio.1001493.s004.jpg]

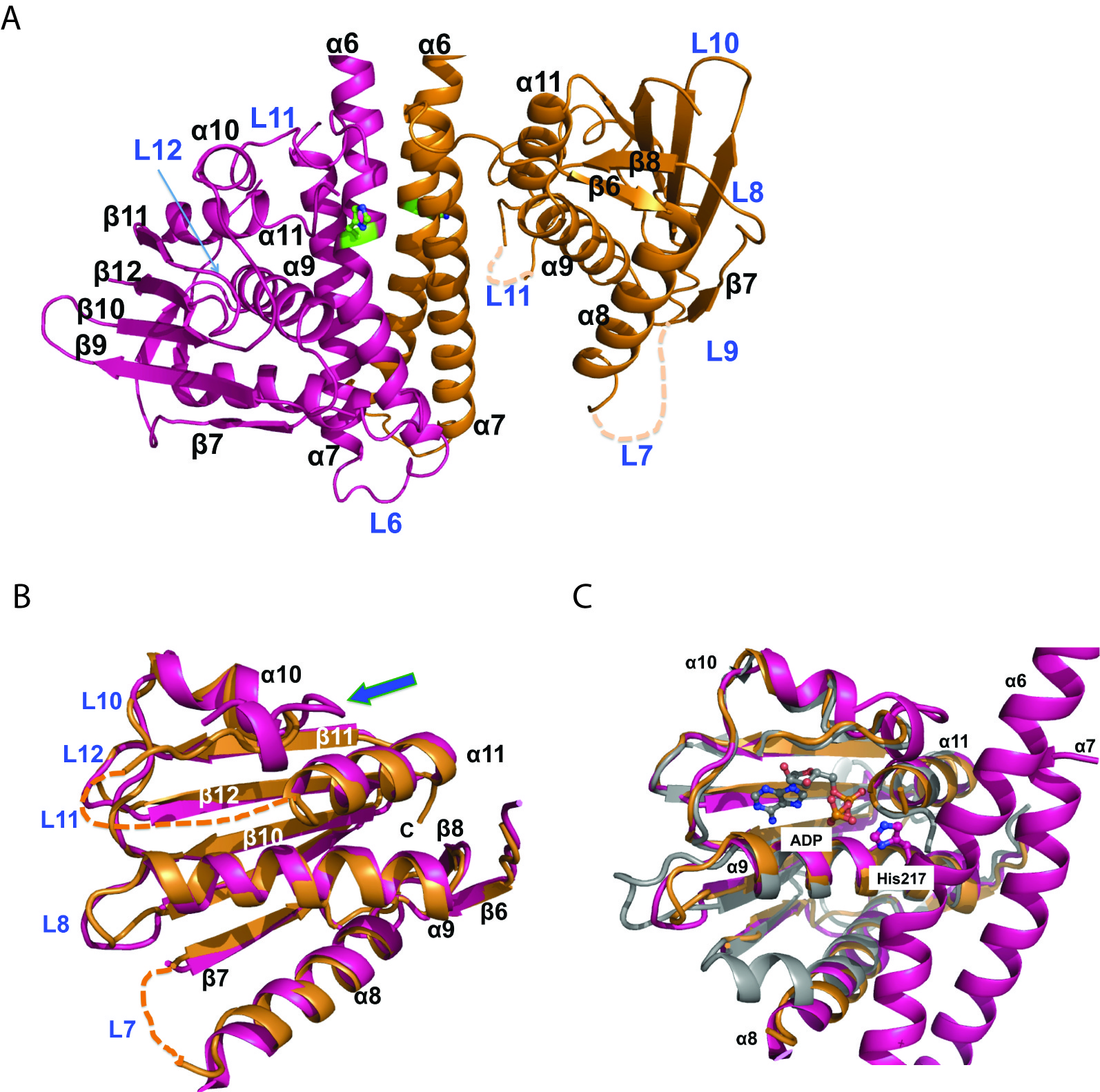

Supplement: Figure S5 — Structure of the VicK C terminal DHp/CA domain. (A) C terminal domains of VicK shown in ribbon. The color scheme is the same as that described for Figure 1C. Two disordered loops L7 and 11 are labeled in dashed lines. The phosphoryl acceptor His217 is shown in green sticks. (B) CA domains of VicK with the conserved globular folds. The blue arrow indicates a distinct conformational region between two CA domains. (C) The CA domain of T. maritima HK853 aligned with both VicK CA domains. HK853 CA domain is colored in grey and VicK CA domains in magenta and gold. ADP is taken from the HK853 structure. (JPG) [file pbio.1001493.s005.jpg]

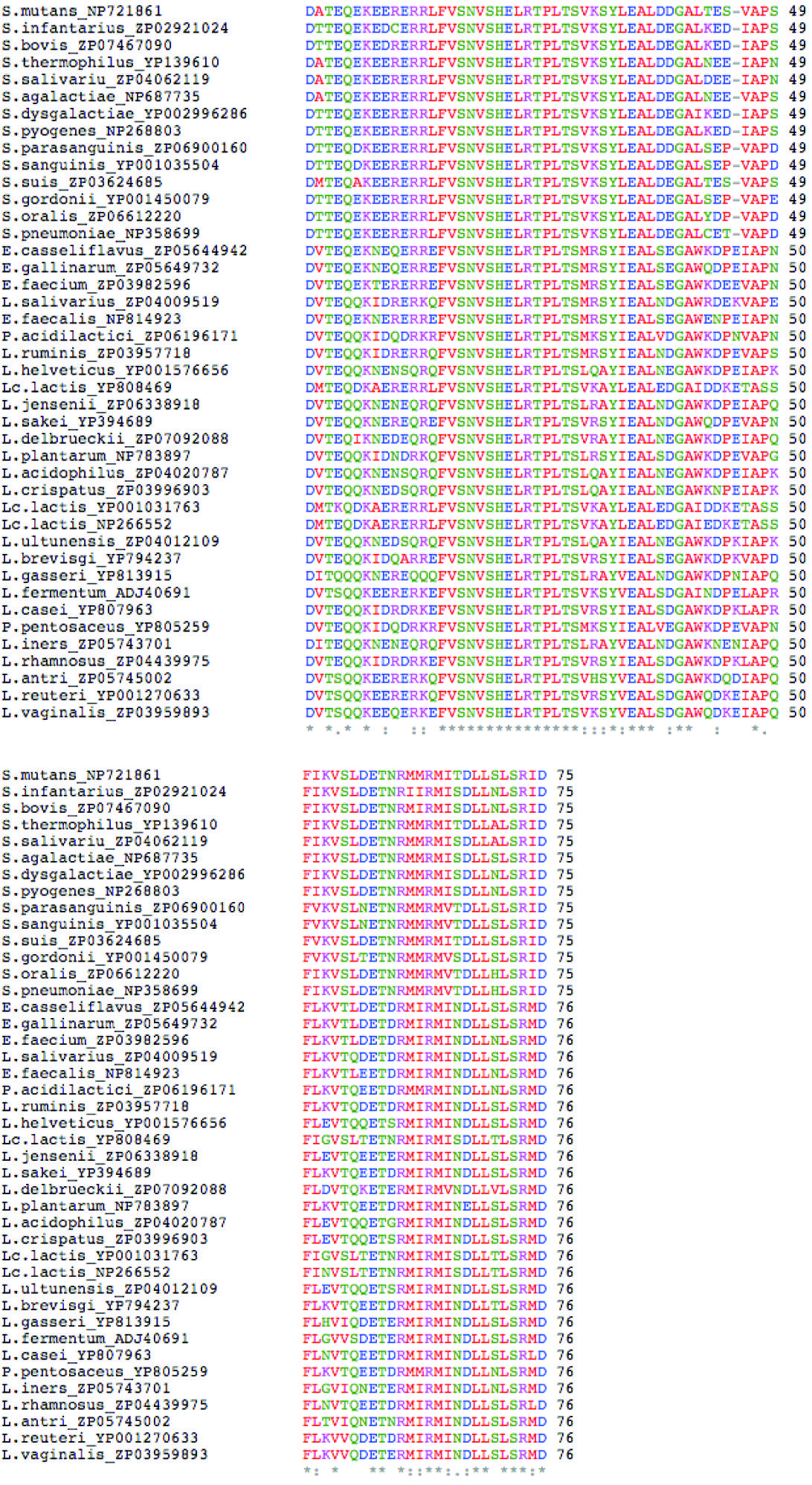

Supplement: Figure S6 — Alignment of the DHp domain of the non-redundant VicK homologs. The alignment is colored by default in CLUSTAL program [70]. The amino acids are grouped into hydrophobic (red), polar (green), basic (blue), and acidic (pink) residues. Highly conserved residues are labeled by asterisks. Similar residues are labeled with colons and less conserved residues are with periods. S, Streptococcus; E, Enterococcus; L, Lactobacillus; Lc, Lactococcus; P, Pediococcus. The alignment of all full-length homologs is presented in Figure S11. (JPG) [file pbio.1001493.s006.jpg]

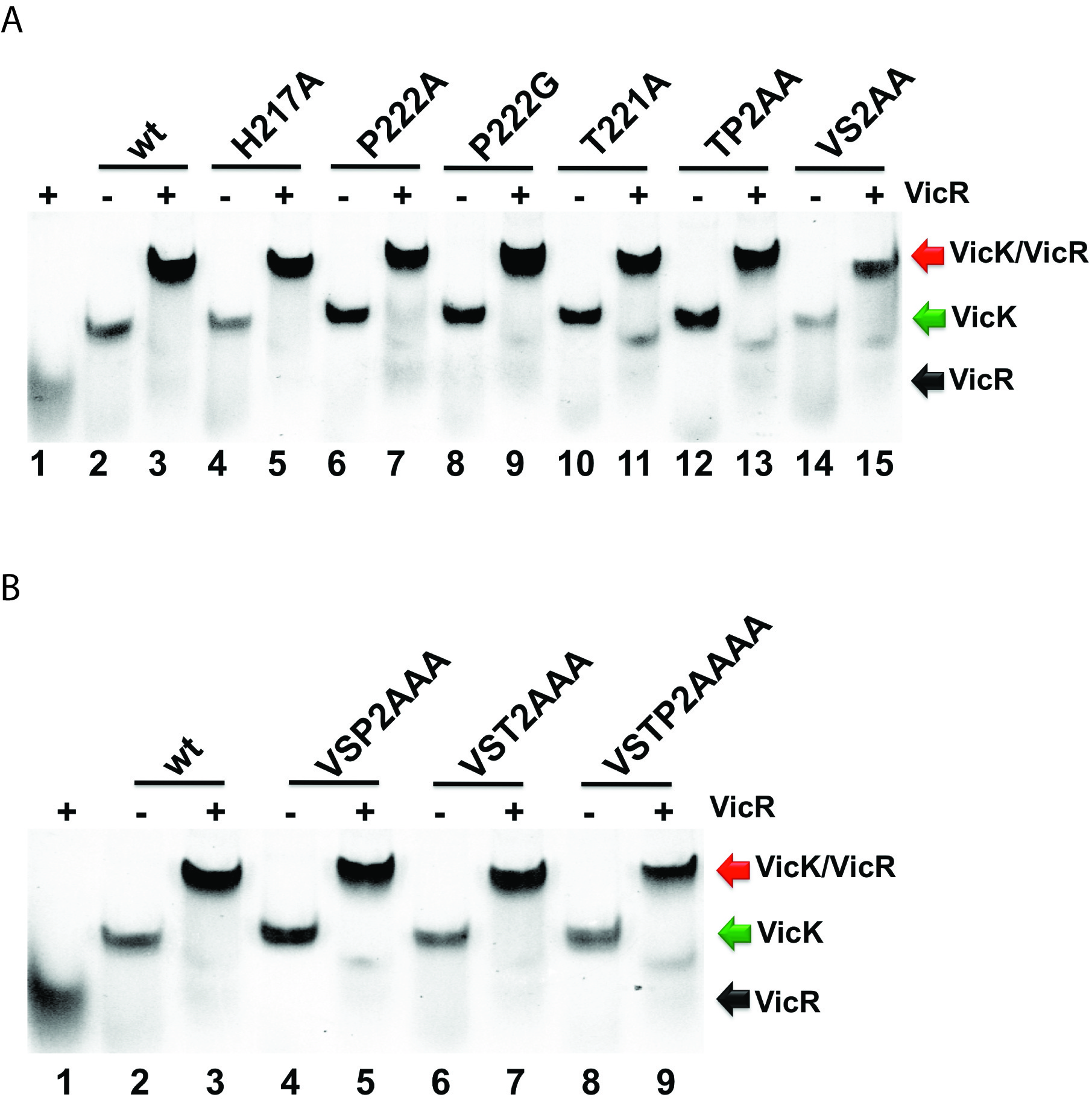

Supplement: Figure S7 — Mutations in DHp domain did not affect the interaction between VicK and VicR. (A,B) The interactions of VicK and VicR were detected by native PAGE. All buffers were essentially the same as regular SDS-PAGE but without SDS. The 10% gels were used and run under 120 V for 120 min at 4°C and stained with coomassie blue. VicR, VicK, and the VicK/VicR complex are indicated by black, green, and red arrows, respectively. The wt VicK and mutants are labeled on top of each gel. (JPG) [file pbio.1001493.s007.jpg]

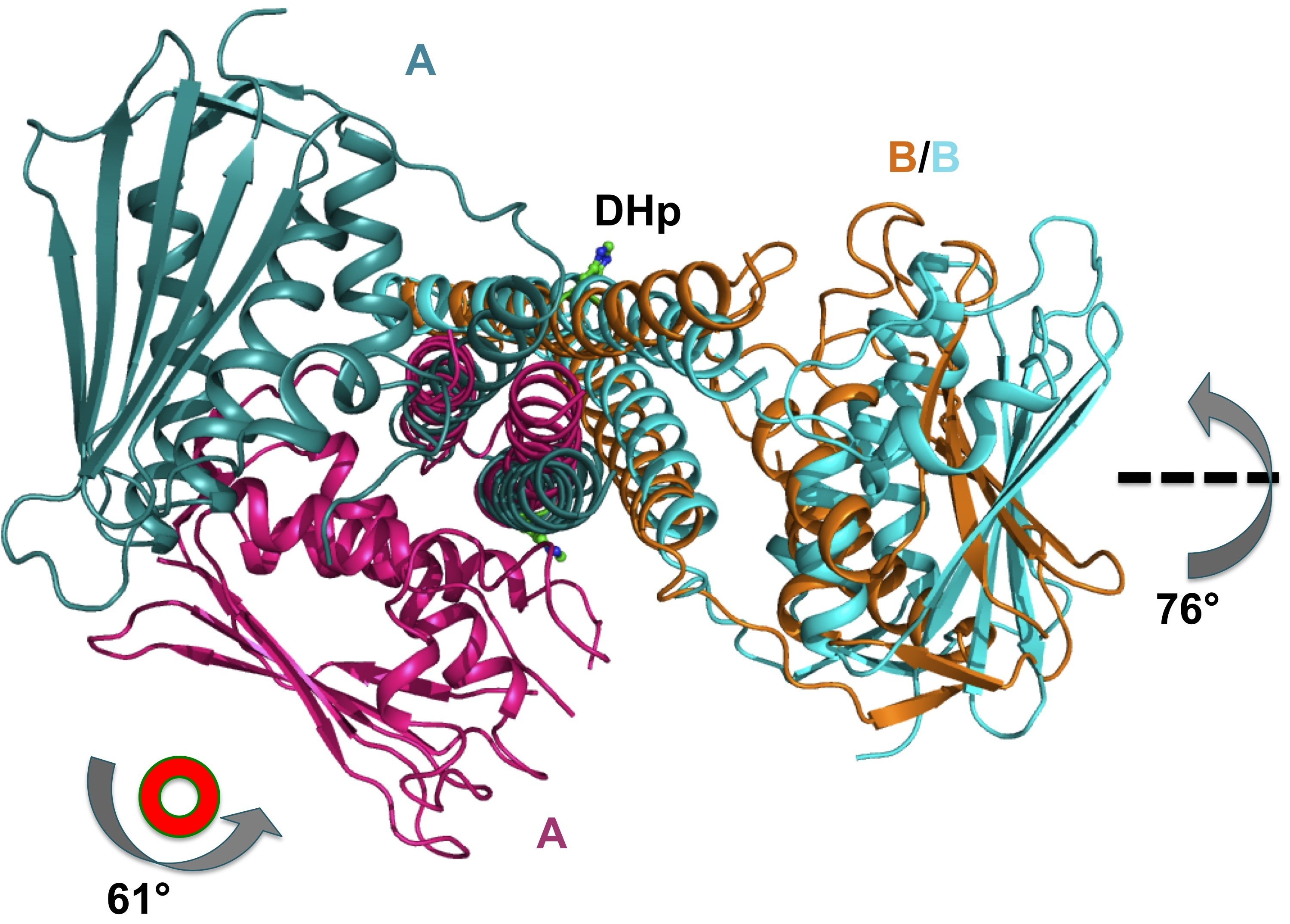

Supplement: Figure S8 — Alignment of the C terminal VicK with HK853. The alignment was performed as described in Figure 4B. The monomers of the VicK are colored in magenta and gold and the monomers of T. maritima HK853 in blue and cyan. The red circle is an axis in parallel to the DHp domain and going to the paper. The black dashed line is an axis perpendicular to the DHp domain. Grey arrows indicate rotation directions and rotation angles are labeled. His217 is highlighted in green sticks. (JPG) [file pbio.1001493.s008.jpg]

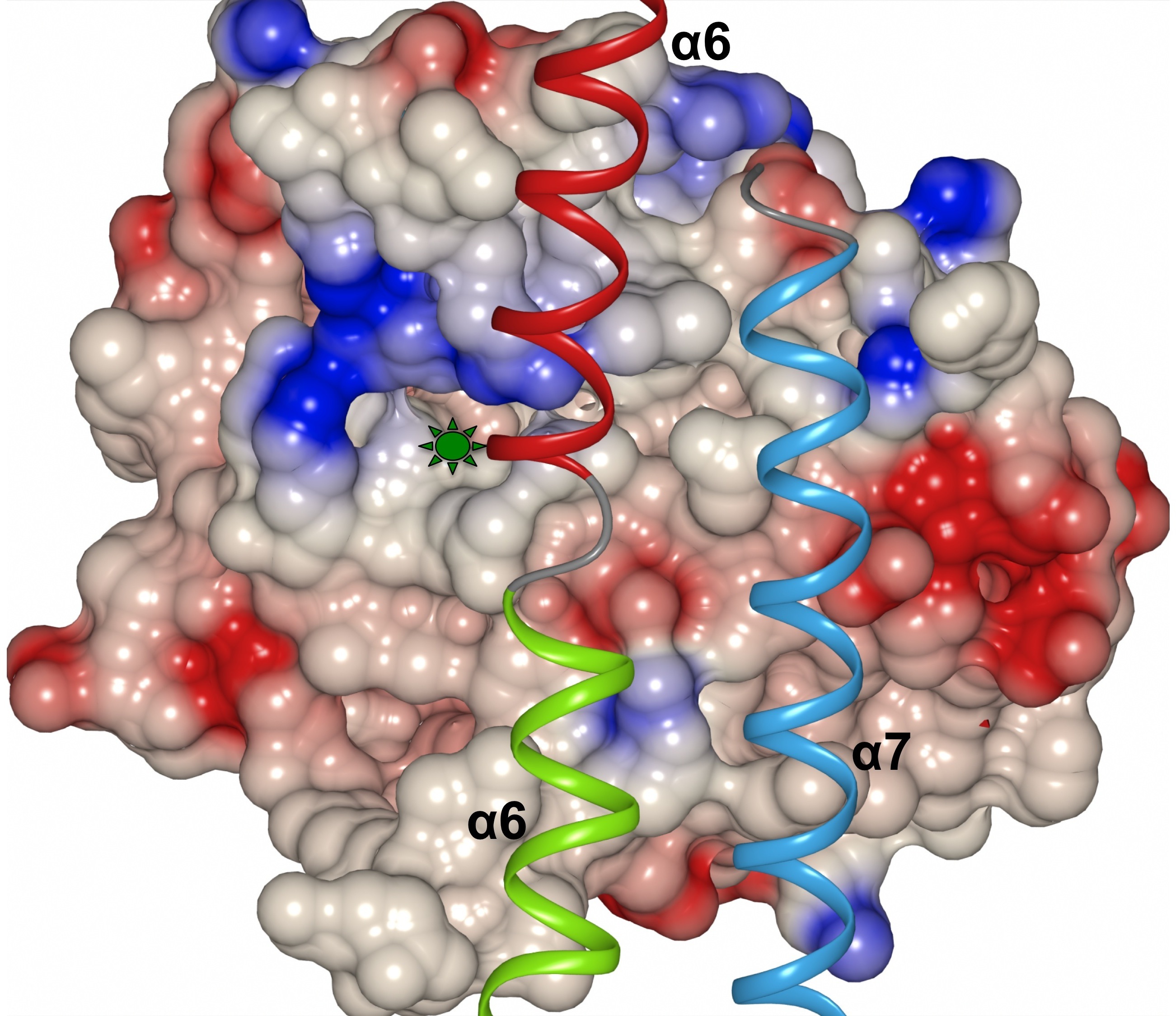

Supplement: Figure S9 — Interface between the DHp and active CA domain. The CA domain is presented in an electrostatic potential surface as described in Figure 1D. The two helices of DHp are in spectrally colored ribbon. The green star indicates His217 position in close proximity to the ATP binding pocket of the CA domain. (JPG) [file pbio.1001493.s009.jpg]

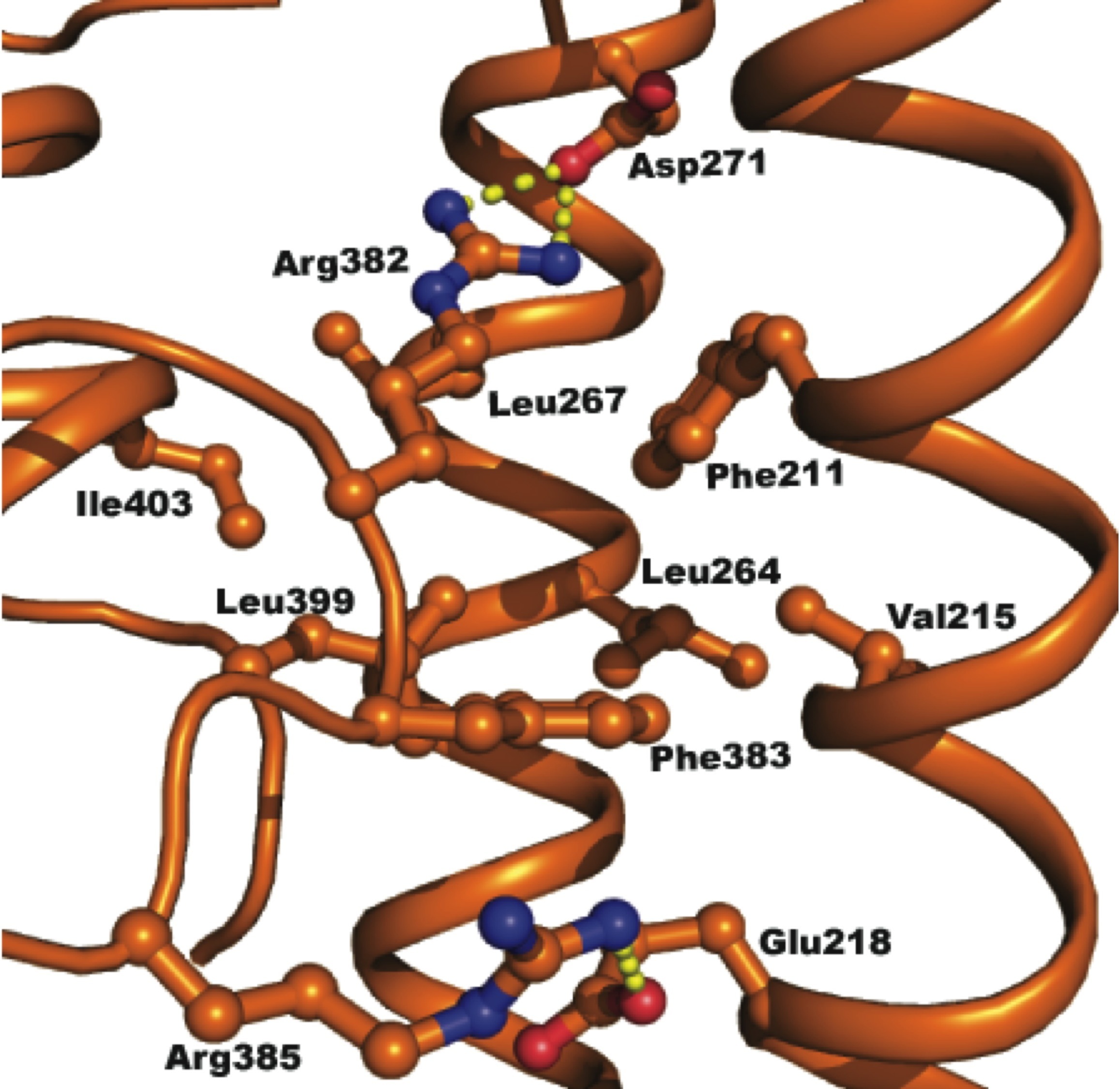

Supplement: Figure S10 — Detailed interactions between the VicK DHp domain and inactive CA domain. Residues involved in hydrogen bonds and hydrophobic interactions are labeled in sticks. Hydrogen bonds are shown in yellow dashed lines. (JPG) [file pbio.1001493.s010.jpg]

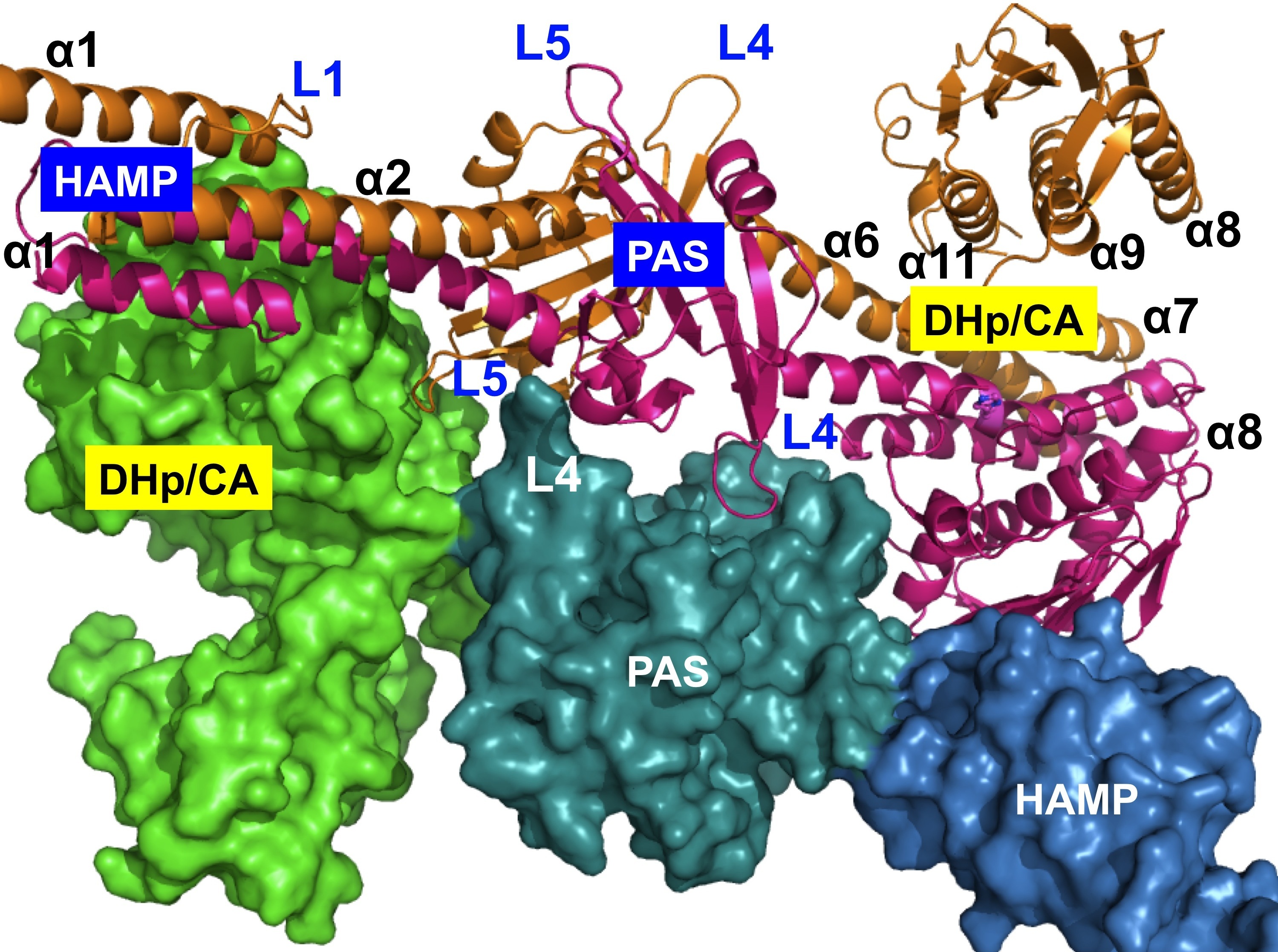

Supplement: Figure S12 — Crystal contacts of two VicK dimers in one asymmetric unit. The top dimer is in ribbon colored and labeled as described in Figure 1C. The HAMP, PAS, and DHp/CA domains of the bottom dimer are shown in molecular surfaces and colored in steelblue, skyblue, and green, respectively. Loop L4 from the bottom dimer is presented in molecular surface and labeled in white. Loops L4 and L5 from the top dimer are presented in ribbon and labeled in blue. (JPG) [file pbio.1001493.s012.jpg]
