# Supplementary material for: Mechanistic Insights Revealed by the Crystal Structure of a Histidine Kinase with Signal Transducer and Sensor Domains
Source: PLoS Biol. 2013 Feb 26;11(2):e1001493. doi: 10.1371/journal.pbio.1001493 (PMC3582566; doi:10.1371/journal.pbio.1001493)
Supplement: Figure S11 — Alignment of S. mutans VicK homologs. The alignment are colored by default in CLUSTAL program [70]. The amino acids are grouped into hydrophobic (red), polar (green), basic (blue), and acidic (pink) residues. Highly conserved residues are labeled by asterisks on the bottom of the alignment. Similar residues are labeled with colons and less conserved residues are with periods. The positions for H, N, G1, F, G2, and G3 boxes are labeled on the top of the alignment. The abbreviations of genera are described in Figure S6. (PDF) [file pbio.1001493.s011.pdf]

```
CLUSTAL 2.0.12 multiple sequence alignment

S.mutans_NP721861              --------------------------------------------------
S.mutans_YP003484506           --------------------------------------------------
S.gallolyticus_YP003430968     --------------------------------------------------
S.bovis_ZP07467090             --------------------------------------------------
S.infantarius_ZP02921024       --------------------------------------------------
S.dysgalactiae_YP002996286     --------------------------------------------------
S.pyogenes_NP268803            --------------------------------------------------
S.equi_YP002744127             --------------------------------------------------
S.equi_YP002123744             --------------------------------------------------
S.uberis_YP002561898           --------------------------------------------------
S.agalactiae_NP687735          --------------------------------------------------
S.thermophilus_YP139610        --------------------------------------------------
S.salivariu_ZP04062119         --------------------------------------------------
S.sanguinis_YP001035504        --------------------------------------------------
S.gordonii_YP001450079         --------------------------------------------------
S.parasanguinis_ZP06900160     --------------------------------------------------
S.oralis_ZP06612220            --------------------------------------------------
S.mitis_ZP07462753             --------------------------------------------------
S.oralis_ZP07458504            --------------------------------------------------
S.mitis_YP003446287            --------------------------------------------------
S.pneumoniae_NP358699          --------------------------------------------------
S.suis_ZP03624685              --------------------------------------------------
S.suis_YP001198724             --------------------------------------------------
L.reuteri_YP001270633          ---------------MNSKLK-----FYQSIRFKIALVFVLILMLTLECV 30
L.vaginalis_ZP03959893         ---------------MNSKLK-----FYQSIKFKIALVFALMLMLTLECV 30
L.antri_ZP05745002             ---------------MNSKLK-----FYQSIRVKIALVFALMLMLTLECV 30
L.fermentum_ADJ40691           ---------------MNSKIK-----FYQSIHFKIALVFALMLMLTLEVV 30
P.acidilactici_ZP06196171      ---------------MNKRTN-----FFRSINFKIALVFAMLLVVTLEVV 30
P.pentosaceus_YP805259         ---------------MNKRTN-----FFKSINFKIALVFAMLLVVTLEVV 30
L.plantarum_NP783897           -------------MLRFKRKN-----FFKTINFKIALVFALLLLITLEIV 32
L.brevisgi_YP794237            ---------------MNSKIR-----WFQSIHFKIALVFALLLLLTLEIV 30
L.salivarius_ZP04009519        ---------MIEAENMNKKVR-----FFHSIHFKIALVFALLLLITFQIV 36
L.ruminis_ZP03957718           -----------MEKQTNKKLH-----FFQSIHFKIALVFALLLLITFQIV 34
L.casei_YP807963               ---------------MNKKIR-----FFQSIHFKIAIVFILLLVVTLELI 30
L.rhamnosus_ZP04439975         ---------------MNKKIR-----FFQSIHFKIAIVFILLLVVTLEMI 30
L.sakei_YP394689               ---------------MNKKIR-----LFQSIHFKIAIVFVLLLLVTLEVI 30
L.acidophilus_ZP04020787       ---------------MKKLRIKLN-TTFNSINTKLAIVFMLMLLATIEVI 34
L.ultunensis_ZP04012109        ------------MRTMKRLKKKLS-TTFNSINIKLAIVFMLMLLATIEVI 37
L.crispatus_ZP03996903         ---------------MKKFKARLN-STFNSINTKIAIVFMLMLLATIEVI 34
L.helveticus_YP001576656       ---------------MKKLREKLN-ATFNSINTKLAVVFMLMLLATIEVI 34
L.jensenii_ZP06338918          ------------MVKMKKIKS-----ILNSLNTKIAVVFMLMILATIEII 33
L.jensenii_ZP04646123          ------------MVKMKKIKS-----ILNSLNTKIAVVFMLMILATIEII 33
L.delbrueckii_ZP07092088       MDGLMKKDKKARKEKVKKEKTNLLKRAVSSIQTKIALIFMLLLLATIEII 50
L.gasseri_YP813915             ----------MQLGQMKKIKS-----VLNSINFKIAVIFMLLLLATIEVV 35
L.johnsonii_NP964082           ---------------MKKIKS-----VLNSINFKIAIIFMLLLLATIEVV 30
L.iners_ZP05743701             ---------------MKKIRK-----PFTSINAKIAIIFMLLLLATIEIV 30
E.casseliflavus_ZP05644942     ---------------MKNKVR-----FFQSVNFKIALTFILILLIAIQII 30
E.gallinarum_ZP05649732        ---------------MKNKVR-----FFRSVNFKIALTFILILLISIQII 30
E.faecium_ZP03982596           ---------------MKRKVR-----FFRSVNFKIAITFILILLISIEII 30
E.faecalis_NP814923            ---------------MKKKVH-----FFQSVNFKIALSFILLLLIAIQII 30
Lc.lactis_YP808469             --------------------------------------------------
Lc.lactis_YP001031763          --------------------------------------------------
Lc.lactis_NP266552             --------------------------------------------------


S.mutans_NP721861              --------------------------------------------------
S.mutans_YP003484506           --------------------------------------------------
S.gallolyticus_YP003430968     --------------------------------------------------
S.bovis_ZP07467090             --------------------------------------------------
S.infantarius_ZP02921024       --------------------------------------------------
S.dysgalactiae_YP002996286     --------------------------------------------------
S.pyogenes_NP268803            --------------------------------------------------
S.equi_YP002744127             --------------------------------------------------
S.equi_YP002123744             --------------------------------------------------
S.uberis_YP002561898           --------------------------------------------------
S.agalactiae_NP687735          --------------------------------------------------
S.thermophilus_YP139610        --------------------------------------------------
S.salivariu_ZP04062119         --------------------------------------------------
S.sanguinis_YP001035504        --------------------------------------------------
S.gordonii_YP001450079         --------------------------------------------------
S.parasanguinis_ZP06900160     --------------------------------------------------
S.oralis_ZP06612220            --------------------------------------------------
S.mitis_ZP07462753             --------------------------------------------------
```

```
S.oralis_ZP07458504           --------------------------------------------------
S.mitis_YP003446287           --------------------------------------------------
S.pneumoniae_NP358699         --------------------------------------------------
S.suis_ZP03624685             --------------------------------------------------
S.suis_YP001198724            --------------------------------------------------
L.reuteri_YP001270633         GAVFVRQLEHQNLNTFKQTIELPS-YVDNSLAEQLSRSNT------KKAN 73
L.vaginalis_ZP03959893        GAVFVRQLEHQNLNTFKQTIELPS-YVDNSLSEQLVNTNH------KKAN 73
L.antri_ZP05745002            GAVFVRQLEHQNLNTFKQTIELPS-YVDNSLSEQLSRSNT------KKAD 73
L.fermentum_ADJ40691          GAVFVRQLEHQELANFKQQIELPS-YIDNSLATQLTSTDT------KTAN 73
P.acidilactici_ZP06196171     GVIFVRQLETQNLSQFKSQVQLQP-YVENEISAQLERSNT------KKAN 73
P.pentosaceus_YP805259        GVIFVRQLETQNLNQFKTQVQLQP-YVENEISTQLERANT------KSAN 73
L.plantarum_NP783897          GAIFVKQLESKNIQDFKQSVQIPT-YIDNSLSEQLTVSST------RKAN 75
L.brevisgi_YP794237           GAVFVRQLERQNLNTFKAQQVVQT-HIVNRLSDELSMSGM------TKSN 73
L.salivarius_ZP04009519       GAIFVRQLKNDNLKSFKQRVELST-YVENSLIVALSRTDT------EGAN 79
L.ruminis_ZP03957718          GAVFVQRLKSENIKSFKQRVELST-YVDNSLIKQLTSTNT------TKAN 77
L.casei_YP807963              GAYFVKTLEQQNIEQFKTSVNVDS-YIQDKLAADLLRTDT------EGAN 73
L.rhamnosus_ZP04439975        GAYFVKTLEQQNIEQFKTSVNVDS-YIQDKLAADLLRTDT------DNAN 73
L.sakei_YP394689              GASFVKQLEYQNVKDFKQSLQVTP-YVQNQLSNELMS-NS------GNAN 72
L.acidophilus_ZP04020787      GAYFTRQLEQTSIENFQSSIQIQT-IVSNQLANQLAS-DN------KNAN 76
L.ultunensis_ZP04012109       GAYFTRQLEQSSIENFQSSIQIQT-IVSNQLANQLAS-DN------KNAN 79
L.crispatus_ZP03996903        GAYFTRQLEQNSIENFQSSIQIQT-IVSNQLANQLTS-DN------KNTN 76
L.helveticus_YP001576656      GAYFTRQLEQNSIENFQSSIQIQT-IVSNQLANQLAS-DS------KNAN 76
L.jensenii_ZP06338918         GAYFTRQLEQSTIESFQTSIQIPT-IITNSLATQLSK-NT------KKAD 75
L.jensenii_ZP04646123         GAYFTRQLEQSTIESFQTSIQIPT-IITNSLATQLSK-NT------KKAD 75
L.delbrueckii_ZP07092088      GAYFTRQMEQTSINNFQTTIQLQS-IVTDQLTAQLVK-NG------KSAD 92
L.gasseri_YP813915            GASFTRQLEQNSIQNFESSIQVPN-IITNQISSQLSRANS------KKAN 78
L.johnsonii_NP964082          GASFTRQLEQNSIQNFESSIQVPN-IITNQIASQLSRANS------KSAN 73
L.iners_ZP05743701            GAYFTRQLEQNSIESFETSIQIPN-LISNQIATQLNKNNA------KKAN 73
E.casseliflavus_ZP05644942    GAYFIRELERTTVDAFKRDMEARASTLVVTLGSELSRMDNDDGLDWDSMN 80
E.gallinarum_ZP05649732       GAYFIRELEKTTIDAFKRDMEARASTLVVTLGSELSRMDAEDSLDWDAMN 80
E.faecium_ZP03982596          GAYFIRGLERSTINTFIKDMNQTVESLATTISPELNRKDN---ADDEEVN 77
E.faecalis_NP814923           GGYFIRELEATTISDFKKNMDSQVVQLSNTLSTQMSNKD----LERSDVD 76
Lc.lactis_YP808469            ------------MIKFFNSMIFKG-------------------------- 12
Lc.lactis_YP001031763         ------------MIKFFNSMIFKG-------------------------- 12
Lc.lactis_NP266552            ------------MIKFFNSMIFKG-------------------------- 12


S.mutans_NP721861             --------------------------------------------------
S.mutans_YP003484506          --------------------------------------------------
S.gallolyticus_YP003430968    --------------------------------------------------
S.bovis_ZP07467090            --------------------------------------------------
S.infantarius_ZP02921024      --------------------------------------------------
S.dysgalactiae_YP002996286    --------------------------------------------------
S.pyogenes_NP268803           --------------------------------------------------
S.equi_YP002744127            --------------------------------------------------
S.equi_YP002123744            --------------------------------------------------
S.uberis_YP002561898          --------------------------------------------------
S.agalactiae_NP687735         --------------------------------------------------
S.thermophilus_YP139610       --------------------------------------------------
S.salivariu_ZP04062119        --------------------------------------------------
S.sanguinis_YP001035504       --------------------------------------------------
S.gordonii_YP001450079        --------------------------------------------------
S.parasanguinis_ZP06900160    --------------------------------------------------
S.oralis_ZP06612220           --------------------------------------------------
S.mitis_ZP07462753            --------------------------------------------------
S.oralis_ZP07458504           --------------------------------------------------
S.mitis_YP003446287           --------------------------------------------------
S.pneumoniae_NP358699         --------------------------------------------------
S.suis_ZP03624685             --------------------------------------------------
S.suis_YP001198724            --------------------------------------------------
L.reuteri_YP001270633         KQINQILSEVNNNNISEIRVVDSKSIVRGTSNADNRSAIGQKTTDQAIKA 123
L.vaginalis_ZP03959893        QKIRKILAEVNNNNISEIRVIDSQGVVRGTSNFDNRNMIGQKTTEQMVKA 123
L.antri_ZP05745002            QEIRQILSEVNNNNISEIRVVDSKGVFRGTSTADNRNIVGQKTTDAMVKA 123
L.fermentum_ADJ40691          KEIKTILARVNNTSITEIRVVDAKGIIRGTSSSGNQGIVGQKTTDTVIKN 123
P.acidilactici_ZP06196171     DQIADIIGNINNQNITEIRVIDAKGVIRGTSNNADRSMVGQKTTDRNVKD 123
P.pentosaceus_YP805259        SQISDIIGNINNQNITEIRVIDAKGVIRGTSNSTNQNIVGQKTTDRNVKD 123
L.plantarum_NP783897          GQIKTILSNYNNTNVDEIQVVDSKGTIRGTSDVNDQSVVGQKTTDRNVKN 125
L.brevisgi_YP794237           KQIKSTLSEINNTNNAQIRVIDNKGIIRGTNEVDNQGIVGQKTTDNNIKN 123
L.salivarius_ZP04009519       TKIKNILDDINNANIQEVQVIDAKGTIRGDSDVNSRSLIGQKTTNSAVKG 129
L.ruminis_ZP03957718          KSINTILEDINNENISTIQVIDSKGTIRGENDVNGRSVVGQKTTNTNIKR 127
L.casei_YP807963              DDIKSTLSQADIANSADLQVVDAKGTIRGDSDINAQTNVGQKSANSSIKN 123
L.rhamnosus_ZP04439975        DDIKSTLSQADIANSADLQVIDAKGTIRGDSDINAQTNVGQKSANSNIKN 123
L.sakei_YP394689              KNIKDIIG--DMGNSAEIQVVDNKGTIRGVTNLNTQSTVGQKTRNPRVKQ 120
L.acidophilus_ZP04020787      DRLNQIVNDYNNDAISEIIVVDNKDTIRAVSNLNDKSKIGQRINNTDVKQ 126
L.ultunensis_ZP04012109       DRLNQIVNDYNNDLISEIIVVDNKDTIRAVSNLNDKSKIGQRINNTDVKQ 129
```

```
L.crispatus_ZP03996903         DRLNQIVNDYNNDAISEIIVVDNKDTIRAVSNLNDKSKIGQRINNTDVKQ 126
L.helveticus_YP001576656       DRLNQIVNDYNNDSISEIIVVDNKDTIRAVSNLNDKSKVGQRMNNTDVKQ 126
L.jensenii_ZP06338918          EQLSQIVSNYNNSAISEIIVVDNKDVIRAVSNLNDKSRVGQRATNVGIKQ 125
L.jensenii_ZP04646123          EQLSQIVSNYNNSAISEIIVVDNKDVIRAVSNLNDKSRVGQRATNVGIKQ 125
L.delbrueckii_ZP07092088       RKMNQIITDYSNDAISEIQVVDSKNVIRAVSNLNDKNRVGQLSNNGDIRE 142
L.gasseri_YP813915             QQLSQIISNYNLGDISQLMVVDNKGVIRAVSNVNDQNRIGQRTSNVDIKS 128
L.johnsonii_NP964082           QQLSQIISNYNLGDISQLMVVDNKGVIRAVSNVNDQNRIGQRTSNADIKS 123
L.iners_ZP05743701             KQLNRIVSNYSNDAINDITVVDSKSVIRSVSNVNDQGTIGQRATNSDIKK 123
E.casseliflavus_ZP05644942     ASLNRILTSTNSSEILEMRVVDEQGIIRATTNINDRGSVGEKNDYRDYDT 130
E.gallinarum_ZP05649732        SSLNRILSSTNSSEILEMRVVDEQGIIRATTNINDRGSVGEKNDYRDYDT 130
E.faecium_ZP03982596           ANIKRFIENSATSDIIEIRVVDEKGIIRGTTDVNEQSAVGKKNDYVDIND 127
E.faecalis_NP814923            ANLKKALSDFSNADISEARIVDDKGIIRATNDLNQQNIIGKKNDYRDLND 126
Lc.lactis_YP808469             --------------------------------------------------
Lc.lactis_YP001031763          --------------------------------------------------
Lc.lactis_NP266552             --------------------------------------------------


S.mutans_NP721861              -------------------------------------------------M 1
S.mutans_YP003484506           -------------------------------------------------M 1
S.gallolyticus_YP003430968     -------------------------------------------------M 1
S.bovis_ZP07467090             -------------------------------------------------M 1
S.infantarius_ZP02921024       -------------------------------------------------M 1
S.dysgalactiae_YP002996286     -------------------------------------------------M 1
S.pyogenes_NP268803            -------------------------------------------------M 1
S.equi_YP002744127             -------------------------------------------------M 1
S.equi_YP002123744             -------------------------------------------------M 1
S.uberis_YP002561898           -------------------------------------------------M 1
S.agalactiae_NP687735          --------------------------------------------------
S.thermophilus_YP139610        --------------------------------------------------
S.salivariu_ZP04062119         --------------------------------------------------
S.sanguinis_YP001035504        -------------------------------------------------M 1
S.gordonii_YP001450079         -------------------------------------------------M 1
S.parasanguinis_ZP06900160     -------------------------------------------------M 1
S.oralis_ZP06612220            -------------------------------------------------M 1
S.mitis_ZP07462753             -------------------------------------------------M 1
S.oralis_ZP07458504            -------------------------------------------------M 1
S.mitis_YP003446287            -------------------------------------------------M 1
S.pneumoniae_NP358699          -------------------------------------------------M 1
S.suis_ZP03624685              -------------------------------------------------M 1
S.suis_YP001198724             -------------------------------------------------M 1
L.reuteri_YP001270633          TLLNNRSHTENLYDNSNHTRYYVNVIPLVDN----SNNNVVGAVYLRASL 169
L.vaginalis_ZP03959893         TLVNNRSHTENVYDNSNHTRYYVNIIPLVDN----SNNNVVGVVYLRASL 169
L.antri_ZP05745002             TLLNNRSHSENVYDGANHNRYYVNVIPLND-----SNNNVVGAVLLRASL 168
L.fermentum_ADJ40691           TLVNNRSHTENLYDSANHNRYYVSVVPLLASG---NANNVVGVVYMRANL 170
P.acidilactici_ZP06196171      VIYNTRSYQQISYNKTNNTRYFISVVPLINTS--GATNNLTGVVYIRANL 171
P.pentosaceus_YP805259         VIYNTRTYQQVSYNKTTNTRYFVSIVPLINTA--GATNNLTGVVYIRANL 171
L.plantarum_NP783897           AIYNNRTYTKSTYDTQNNGRYYVSIVPLYSSNSAGNSSQLVGVLYVRANM 175
L.brevisgi_YP794237            AIYNNRDVVRTTYSSQSNSRYYTTITRLIRTQ--GNNSELVGVIYMRSSL 171
L.salivarius_ZP04009519        VLYTGRTYTETTYNRNDNKQYYISITPLLNVS--SNNNAVVGAIYTRADM 177
L.ruminis_ZP03957718           VLYSGRTFTTMTYDKSSNRRSYVSITPLFSAS--GNTNTVVGAVYLRANL 175
L.casei_YP807963               TLYSGQQYEGYEYRDRTGS-TYYKVIPIKNPNATGDNNSVIGAIAISASM 172
L.rhamnosus_ZP04439975         TLYSGQQYEGYEYRERTGS-TYYKVVPIKNPNATGDNNSVIGAIAISASM 172
L.sakei_YP394689               AIYSGHTEQEVAYSESQGS-YYTAIQPLTDPN--GDSNTVVGAVYIRASM 167
L.acidophilus_ZP04020787       VISTGHQINKVI--DDH-GNYMIQISPLTSGNG---SNNNVGAIYVKASM 170
L.ultunensis_ZP04012109        VISTGHQINKVV--DDH-GNYMIQISPLTSGNG---SNNNVGAIYVKASM 173
L.crispatus_ZP03996903         VISTGHQINKVV--DDH-GNYMIQISPLTSGNG---SNNTVGAVYVKASM 170
L.helveticus_YP001576656       VISTGHQINKVV--NDH-GNYMIQISPLTSGNG---SNNNVGALYVKASM 170
L.jensenii_ZP06338918          VTSTGRQITWID--SNNNGSNMVQITPLTIGTG---TNSTLGAIYVRANM 170
L.jensenii_ZP04646123          VTSTGRQVTWID--SNNTGSNMVQITPLTVGTG---ANSTLGAIYVRANM 170
L.delbrueckii_ZP07092088       VINSGQQKTQVA--SDQKGSFMVQIVPLTSGT------NIVGAVYVRASM 184
L.gasseri_YP813915             VLSNGKQVSKVI--NDN-GNYMVQISPLTSANG---TNTPVGAIYVRASL 172
L.johnsonii_NP964082           VLSNGKQVSKVI--NDN-GNYMVQISPLTSANG---TNTPVGAIYVRASL 167
L.iners_ZP05743701             VLTSGRQISKII--NDN-GKYMIQISPLNVGNG---PNTIVGVIVVKAGM 167
E.casseliflavus_ZP05644942     NRS-----EVLYDESSG-QRVFNIVQSIQSPTG----DTVLGTLYLRSNL 170
E.gallinarum_ZP05649732        NRS-----EVLYDESTG-QRVYNIVQSIQSPTG----DTVLGTLYLRSNL 170
E.faecium_ZP03982596           FTT-----KRYVALDNDDKRVNINVQPILSPTG----DAVIGALYVKSNI 168
E.faecalis_NP814923            FTS-----KKYQALDND-KRVYVNVQPIQSPTG----ETVIGVLYVKSNL 166
Lc.lactis_YP808469             ------------------------------------------VVAHLLLF 20
Lc.lactis_YP001031763          ------------------------------------------VVAHLLLF 20
Lc.lactis_NP266552             ------------------------------------------VVAHLLLF 20

S.mutans_NP721861              TNVFESSPLFLRILLAVLIILLFFYFIFLNYREYKNNNQVKQLNAKVRSL 51
S.mutans_YP003484506           TNVFESSPLFLRILLAVLIILLFFYFIFLNYREYKNNNQVKQLNAKVRSL 51
S.gallolyticus_YP003430968     NDTMLQNLTYFEQAILFLLAFVAVYFVYLAIRDYRTSVNIRRLSGKVREL 51
S.bovis_ZP07467090             NDTMLQNLTYFEQAILFLLAFVAVYFVYLAIRDYRTSVNIRRLSGKVREL 51
```

```
S.infantarius_ZP02921024        NDTMVQNQYLFEQAILFLLAFVAIYFIHLAIRDYRTSVNIRRLSGKVREL 51
S.dysgalactiae_YP002996286      TRDIIGNLSAFELAILLLLVFVAFYFIHLAVRDYRNARIIRMMSHKIRDL 51
S.pyogenes_NP268803             TRDIIGNLSTFELAILILLVFVAFYFIHLAVRDYRNARIIRMMSHKIRDL 51
S.equi_YP002744127              TKDIIGNLSAFELAILLLLVFVAFYFIHLAIRDYRNARIIRLMSHKIRDL 51
S.equi_YP002123744              TKDIIGNLSAFELAILLLLVFVAFYFIHLAIRDYRNARIIRLMSHKIRDL 51
S.uberis_YP002561898            TENLIGNLSLFELSILLLLIFVAAYFIYLAVRDYRNAKIIRQMSHKIRDL 51
S.agalactiae_NP687735           MNNSAANIRSFELALLFLLVFVAVYFVYLAVRDFKMSKNIRLLNWKVRDL 50
S.thermophilus_YP139610         MTSIGLNITSFELALLFMLLFVAFYFIFLAYRDYQQVKNIRKLTKRVKSL 50
S.salivariu_ZP04062119          MTSIGLNITSFELALLFTLLFVAFYFIFLAYRDYRRVKNIRKLTKRVKSL 50
S.sanguinis_YP001035504         IEAIKQFVISADFVFAIIIIGFIVVVALLLLENRRDNQKLVQLNQKVKDL 51
S.gordonii_YP001450079          IENIRQFVFSVDFVFVLIVLGFILVVALLFLENRRDNRQLRLLNQKIKDL 51
S.parasanguinis_ZP06900160      IDQLKQFVMSSNFVFVLITVGFIIVVALLLLENRRDNIKLRQLNSKIKDL 51
S.oralis_ZP06612220             IEDIRQTILTSDFIFILILLGFILVVTLLLLENRRDNIRLKEINQKVKDL 51
S.mitis_ZP07462753              IEDIRQTILTSDFIFILILLGFILVVTLLLLENRRDNIRLKEINQKVKDL 51
S.oralis_ZP07458504             IEDIRQTILTSDFIFILILLGFILVVTLLLLENRRDNIRLKEINQKVKDL 51
S.mitis_YP003446287             IKLIKDTVLTSDSIFILILLGFILVVTLLLLENRRDNIRLKQINQKVKDL 51
S.pneumoniae_NP358699           LDLLKQTIFTRDFIFILILLGFILVVTLLLLENRRDNIQLKQINQKVKDL 51
S.suis_ZP03624685               INQLRYLMTTAEFWFVVILIGFLIALTVLLIENYRDNKQIKQLNQKVNAL 51
S.suis_YP001198724              INQLRYLMTTAEFWFVVILIGFLIALTVLLIENYWDNKQIKQLNQKVNAL 51
L.reuteri_YP001270633           ESVYSNINNITLVYLSAAMITIVLSLILAIIISQEITRPIEEMRKQTLRI 219
L.vaginalis_ZP03959893          ESVYSNINSITLIYLSAALITIVIGLLLAIIISQEITRPIEEMRKQTLRI 219
L.antri_ZP05745002              EGVYSNINNITLIYFSAALVTIILSLFLAVLISQEITRPIEEMRKQTLRI 218
L.fermentum_ADJ40691            EGVYSTINSISLIYLSAALITIVLGLGLAVLISREITRPIEEMRKQTLRI 220
P.acidilactici_ZP06196171       ESVYQNVNNITLIFVVAALIAITIGLFLAVLIARAITRPIEEMRQRTMQI 221
P.pentosaceus_YP805259          ESVYQNVNNITLIFVVAALIAITIGLILAVLISRAITRPIEEMRQRTIQI 221
L.plantarum_NP783897            KSVYSTINNIMGIFVIASLAAMVLGLGIAIIIARAITRPIEEMKQQTQRI 225
L.brevisgi_YP794237             DSVYQNINQITLIYVSATFVAIILGLVMAIIISRAITRPIDEMKKQTLRI 221
L.salivarius_ZP04009519         ASVYDSVNNVVVIFASASLIAIVIGMFLAIIVSSSITKPIEEMKRQTARI 227
L.ruminis_ZP03957718            ESVYDSVNSVTLIFATASLIATAIGMALAITVSSAITRPIDEMKKQTMQI 225
L.casei_YP807963                EQAYDSINKIVGIFLISSLVAGLLGTLLSIVISRAITRPIDEMKKQAIRM 222
L.rhamnosus_ZP04439975          EQAYDSINKIVGIFLISSLVAGLLGTLLSIVISRTITRPIDEMKKQAIRM 222
L.sakei_YP394689                EEVYKGISSITVIFLTSSLVAGLLGMALSIVISRAITRPIDEMKKQAIQM 217
L.acidophilus_ZP04020787        QDVFNNLRQISLTFLIASLIAALLGAFLALVISRAITQPIEEMQKQALHI 220
L.ultunensis_ZP04012109         QDVFNNLRQISLTFLIASLIAALLGAFLALVISRAITQPIEEMQKQALHI 223
L.crispatus_ZP03996903          QDVFNNLRQISLTFLIASLIAALLGAFLSLVISRAITQPIEEMQKQALHI 220
L.helveticus_YP001576656        QDVFDNLRQISLTFLIASLIAALLGAFLALVISRAITQPIEEMQKQALHI 220
L.jensenii_ZP06338918           QGVFDNLRNISYMFLTASFAASILSAILALFISRAITKPIEEMRMQALQV 220
L.jensenii_ZP04646123           QGVFDNLRNISYMFLTASFAASILSAILALFISRAITKPIEEMRMQALQV 220
L.delbrueckii_ZP07092088        ADVFNNLRNISMMFLTASLLAVVVAAIASMFISRAITRPIEELRQQAIQV 234
L.gasseri_YP813915              QGVFNNLRQVSIYFLIASLIAAVLGAIVALVISRAITRPIEEMRKQALRV 222
L.johnsonii_NP964082            QGVFNNLRQVSIYFLIASLIAAVLGAVVALVISRAITRPIEEMRKQALRV 217
L.iners_ZP05743701              QSVFNNIHRVSFLFLTASLVAATLGAGVALVIARAITKPIEEMKDQALRI 217
E.casseliflavus_ZP05644942      ESKYNEVSTTAVIFVTASLIAAVISIIVALLVSRSITQPIGEMREQAIRI 220
E.gallinarum_ZP05649732         EQKYSEVSDTAVIFVTASLIAAAISIVVALLVSRSITQPIGEMREQAIRI 220
E.faecium_ZP03982596            EQKYSEINNTAVIFFTASLIAAFISMIVSVLVARSITQPIGEMREQAIRI 218
E.faecalis_NP814923             ENKYQEITNTASIFFTASIIAAAISIIVTLLIARSITKPIGEMREQAIRI 216
Lc.lactis_YP808469              VILFMSNFDYNLIHLSTFIGYYLFSLVFVLVFLNQQHRMIVNVSKQMNDI 70
Lc.lactis_YP001031763           VILFMSNFDYNLIHLSTFIGYYLFSLVFVLVFLNQQHRMIVNVSKQMNDI 70
Lc.lactis_NP266552              VILFMSSFDYNFAHLSTFIGYYLFSLVFVLVFLNQQHRMIVNVSKQMNDI 70


S.mutans_NP721861               ITGHYTDKLKVEDNSDLSELVNNVNDLSEVFRLTHENLAQEKNRLTSILS 101
S.mutans_YP003484506            ITGHYTDKLKVEDNSDLSELVNNVNDLSEVFRLTHENLAQEKNRLTSILS 101
S.gallolyticus_YP003430968      ITGKYTEDIIIEKDRDLAELADQLNDLSTVFRLAQENLAQEKNRLASILS 101
S.bovis_ZP07467090              ITGKYTEDIIIEKDRDLAELADQLNDLSTVFRLAQENLAQEKNRLASILS 101
S.infantarius_ZP02921024        ITGKYTEDIIIEKDRDLAELAGQLNDLSTVFRLAQENLAQEKNRLASILS 101
S.dysgalactiae_YP002996286      INGRYTDMINEKADIELMELADQLNDLSDVFRLTHENLAQEKNRLASILA 101
S.pyogenes_NP268803             INGRYTDIIDEKADIELMELSDQLNDLSDVFRLTHENLAQEKNRLASILA 101
S.equi_YP002744127              INGRYTDTINEKADIELMELSEQLNDLSDVFRLTHENLAQEKNRLASILA 101
S.equi_YP002123744              INGRYTDTINEKADIELMELSEQLNDLSDVFRLTHENLAQEKNRLASILA 101
S.uberis_YP002561898            INGRYTDEINEKADIELIELSEQLNDLSDVFRLTHENLAQEKNRLASILA 101
S.agalactiae_NP687735           IAGNYSDSILIQGDADLVELGESLNDLSDVFRMAHDNLEQEKNRLASILT 100
S.thermophilus_YP139610         MAGNYNEKLCIKGDSELLELANSLNDLSDVFRLTHENLIQEKNRLSSVLS 100
S.salivariu_ZP04062119          MAGNYNEELRLKGDPELLELADSLNDLSDVFRLTHENLAQEKNRLSSVLS 100
S.sanguinis_YP001035504         IAGDYSEVLDMQGSPEITDMTNSINDLSEVIRLTHENLEQETKRLSSILS 101
S.gordonii_YP001450079          IAGDYSEVLDMQGSPEITDMTNSINDLSEVTRLTQENLEQETKRLTSILS 101
S.parasanguinis_ZP06900160      IAGDYSEVVDMQGSPELTDMTNSINDLSEVIRLTHENLEQETKRLTSILS 101
S.oralis_ZP06612220             IAGDYSQVLDLQGSTEITNITNNLNDLSEVIRLTQENLEQESKRLNSILS 101
S.mitis_ZP07462753              IAGDYSQVLDLQGSTEITNISNNLNDLSEVIRLTQENLEQESKRLHSILS 101
S.oralis_ZP07458504             IAGDYSQVLDLQGSTEITNITNNLNDLSEVIRLTQENLEQESKRLHSILS 101
S.mitis_YP003446287             ITGDYSQVLDMQGSSEITNITNNLNDLSEVIRLTQENLEQESKRLNSILS 101
S.pneumoniae_NP358699           IAGDYSKVLDMQGGSEITNITNNLNDLSEVIRLTQENLEQESKRLNSILF 101
S.suis_ZP03624685               IEGNYADVLDMRGSPEITDMANSLNDLSEVIRLTHDNLEQEKTRLTSILS 101
S.suis_YP001198724              IEGNYADLLDMRGSPEITDMANSLNDLSEVIRLTHDNLEQEKTRLTSILS 101
L.reuteri_YP001270633           ARGDYSGQVKVLGNDELGQLAGAVNNLSVRVEEAQESSDSERRRLDSILS 269
```

```
L.vaginalis_ZP03959893          ARGDFSGQVRVMGNDELGQLAGAVNNLSVRVEEAQESSDSERRRLDSVLS 269
L.antri_ZP05745002              ARGDFSGQVRVMGNDELGQLAGAVNNLSVRVEEAQESSDSERRRLDSVLS 268
L.fermentum_ADJ40691            ARGDFSGQVKVMGNDELGQLAGAVNNLSVRVEESQESSDAERRRLDSVLS 270
P.acidilactici_ZP06196171       ARGDYSGQVQIYGDDELGQLAAAVNDLSVRVEESQELTESERRRLDSVLG 271
P.pentosaceus_YP805259          ARGDYSGQVQIYGDDELGQLAEAVNELSVRVEESQESTESERRRLDSVLG 271
L.plantarum_NP783897            ARGDYAGQVRVYSDDELGQLAKAINNLSIRVEESQESTEAERRRLDSVLS 275
L.brevisgi_YP794237             ARGDYSGHVHVYGNDELGQLASAVNNLSVRVEEAQESTESERRRLDSVMA 271
L.salivarius_ZP04009519         ARGDYSGHVHQYGNDELGQLAQAVNNLSIRVEETQELSESERRRLDSVLS 277
L.ruminis_ZP03957718            ADGDYSGKVHVYGTDELGQLAQAVNNLSVRVEEAQESSDSERRRLDSVLS 275
L.casei_YP807963                ARGDYSGQVRIYGQDELGQLAVAVNNLSVRVEEAQEASEAERRRLDSVLA 272
L.rhamnosus_ZP04439975          ARGDYSGQVRIYGQDELGQLAVAVNNLSVRVEEAQEASEAERRRLDSVLA 272
L.sakei_YP394689                ARGDYSGQVRIYGQDELGQLAIAVNNLSVRVEEAQEASESERRRLDSVMS 267
L.acidophilus_ZP04020787        ADGDYSSQVKIYSNDELGQLGKALNTLSVRIERSQEESDSERRRLDSVLS 270
L.ultunensis_ZP04012109         ADGDYSSQVKIYSNDELGQLGHAFNTLSVRIERSQEESDSERRRLDSVLS 273
L.crispatus_ZP03996903          ADGDYSSQVKIYSNDELGQLAKAFNTLSVRIERSQEESDSERRRLDSVLS 270
L.helveticus_YP001576656        ANGDYSSQVKIYSNDELGQLAKAFNTLSVRIEQSQEESDSERRRLDSVLS 270
L.jensenii_ZP06338918           ADGDYSGQVKIYSNDELGQLAEAFNTLSIRVERSQEQSESERRRLDSVLS 270
L.jensenii_ZP04646123           ADGDYSGQVKIYSNDELGQLAEAFNTLSIRVERSQEQSESERRRLDSVLS 270
L.delbrueckii_ZP07092088        ADGDYSTQVKIYSNDELGQLGAAFNTLSVKIERSNEQSESERRRLDSVLS 284
L.gasseri_YP813915              ANGDYSGHVRVYAQDELGQLAEAFNTLSVRIERTQEISDSERRRLDNVLT 272
L.johnsonii_NP964082            ANGDYSGHVRVYAQDELGQLAEAFNTLSVRIERTQEISDSERRRLDNVLT 267
L.iners_ZP05743701              SNGDYSGHVKVYANDEMGELARAFNILSVRIERSQEVSDSERRRLDNVLM 267
E.casseliflavus_ZP05644942      AKGDYSRKVAIHGKDELGQLAETFNQLGERIEETQEAMESERNRLDSVLS 270
E.gallinarum_ZP05649732         AKGDYSRKVAVHGQDELGQLAETFNQLGERIEETQEAMESERNRLDSVLS 270
E.faecium_ZP03982596            ARGDYSRKVKVHGQDELGQLADTFNQLAERIEEAQDTMEAERNRLDSVLS 268
E.faecalis_NP814923             ARGDYAGKVEVHGKDELGQLAETFNQLSERIEEAQETMEAERNRLDSVLT 266
Lc.lactis_YP808469              KNGYLNRDKIPKGTSEINELYEEILKLGEDIVRTQNNLENQRNQLDSILA 120
Lc.lactis_YP001031763           KNGYLNRDKIPKGTSKINELYEEILKLGEDIVRTQNNLENQRNQLDSILA 120
Lc.lactis_NP266552              KNGYLNRDKIPKGTSEINELYDEILKLGEDIVRTQNNLENQRNQLDSILA 120
                                  * :           :  .   .  *.  :  :::    :  :* .::

S.mutans_NP721861               YMTDGVLATDRSGKITVINDMAQKQLNVTR-EQALECNILDILDDDS-YT 149
S.mutans_YP003484506            YMTDGVLATDRRGKITVINDMAQKQLNVTR-EQALECNILDILDDDS-YT 149
S.gallolyticus_YP003430968      YMTDGVLATDRAGNITMINQTAQQQLNLER-EEALQMNIVDILGSDSTYS 150
S.bovis_ZP07467090              YMTDGVLATDRAGNITMINQTALQQLNLEH-EEALQMNIVDILGADSTYS 150
S.infantarius_ZP02921024        YMTDGVLATDRSGNITMINQTAQKQLNLER-EEALGMNIVDILGDDSDYS 150
S.dysgalactiae_YP002996286      YMSDGVLATDRSGKIIMINETARKQLNLTK-EKALEKNITDLLEGDTPYT 150
S.pyogenes_NP268803             YMSDGVLATDRSGKIIMINETARKQLNLSK-EEALKKNITDLLEGDTSYT 150
S.equi_YP002744127              YMTDGVLATNRSGKIIMINETAQKQLNLNR-EQALEKNITDLLDSDSPYT 150
S.equi_YP002123744              YMTDGVLATNRSGKIIMINETAQKQLNLNR-EQALEKNITDLLDSDSPYT 150
S.uberis_YP002561898            YMSDGVLATDRTGQIMMINETAQKQLNISK-EEALLMNITDLLGQDTPYT 150
S.agalactiae_NP687735           YMTDGVLATDRSGKIVMINETAQQQFNLAY-DEALSMNIVDMLGSGSPYS 149
S.thermophilus_YP139610         YMSDGVIATNRLGQITMINSTAQRLLNLDR-ETATQMSILDILDGENPYT 149
S.salivariu_ZP04062119          YMSDGVIATNRRGQITMINSTAQRLLNLDY-ETATQMSILDLLDGENPYT 149
S.sanguinis_YP001035504         YMTDGVLATNRRGQIITINDMATKQLGVKR-DEVQNMSILDLLSISDEYD 150
S.gordonii_YP001450079          YMTDGVLATNRRGQIIMINDMASKQLGVDP-EEVQNTSILDLLDIADEYD 150
S.parasanguinis_ZP06900160      YMTDGVLATNRRGQIIMVNEMAAKQLNVNP-DEVLNTSILDLLSLGDDYD 150
S.oralis_ZP06612220             YMTDGVLATNRRGQITMINDMAKKQLGVQK-EDVLNKSILELLKIEDEYE 150
S.mitis_ZP07462753              YMTDGVLATNRRGQITMINDMAKKQLGVQK-EDVLNKSILELLKIEDEYE 150
S.oralis_ZP07458504             YMTDGVLATNRRGQITMINDMAKKQLGVQK-EEVLNKSILELLKIEDEYE 150
S.mitis_YP003446287             YMTDGVLATNRRGQITMINDMAKKQLGVQK-EDVLNKSILELLEIVDEYE 150
S.pneumoniae_NP358699           YMTDGVLATNRRGQIIMINDTAKKQLGLVK-EDVLNRSILELLKIEENYE 150
S.suis_ZP03624685               YMSDGVIATDRIGRIIMINDMAQKQLGLSS-QKQEQYHLLEVLDLSDRYT 150
S.suis_YP001198724              YMSDGVIATDRIGRIIMINDMAQKQLGLSN-PKQEQYHLLEVLDLSDRYT 150
L.reuteri_YP001270633           HMSDGVLATDRRGNVTIVNNMALQLLGVENEDDLIGKSIIDVLDIRHDYT 319
L.vaginalis_ZP03959893          HMSDGVLATDRRGNVTIVNNMALQLLGVEHDDELIGKSIIDVLDIRHDYT 319
L.antri_ZP05745002              HMSDGVLATDRRGNITIVNNMALQLLGVDHEDDLIGKSIIDALDIRHDYT 318
L.fermentum_ADJ40691            HMSDGVLATDRRGNLTIVNNTALQFLNIADEEQVLGKSILDVLKIRRQFS 320
P.acidilactici_ZP06196171       YMTDGVLATDRRGRITIVNEMATDFLNLEN-DQIVGKSILDILDLRGSVT 320
P.pentosaceus_YP805259          YMTDGVLATDRRGRITIVNEMATDFLNLEN-DQIVGKSILDILDLRGTFT 320
L.plantarum_NP783897            HMSDGVIATDRRGNITIINETASDFMDVTA-EKAIGNSILDILKIRDDYS 324
L.brevisgi_YP794237             HMTDGVIATDRRGNVTIINETASSFLDHEP-DDAVGKSILDLLDIRDDYT 320
L.salivarius_ZP04009519         HMSDGVIATDRRGGITIVNDMAMNYLDISE-GEVLGKSILDILDIRNQYT 326
L.ruminis_ZP03957718            HMSDGVIATDRRGNISIINDMACEYLDVES-KDVIGTSILDVLKIREMQT 324
L.casei_YP807963                HMTDGVIATDRRGNVIIINQTALNFLNTKN-EDVIGVSILKLLNIAKDYT 321
L.rhamnosus_ZP04439975          HMTDGVIATDRRGNVIIINQTALSFLNTKN-EDVIGVSILKLLNIAKDYT 321
L.sakei_YP394689                HMTDGVIATDRRGNITIINETGQDFLNVTS-DGVLGQSLLKVLKISEEYT 316
L.acidophilus_ZP04020787        HMSDGVLATDRHGNVSVVNHMALTFLNAKE-EDVINKPIAEVLGLK-DTS 318
L.ultunensis_ZP04012109         HMSDGVLATDRHGNVSVVNHTALSYLNVKE-KDVINKPIAEVLGLK-DRS 321
L.crispatus_ZP03996903          HMSDGVLATDRHGNVSVVNHMALNFLNKKE-DEVINKPIAEVLGLE-DTS 318
L.helveticus_YP001576656        HMSDGVLATDRHGNVSVVNHMALSYLNADE-DEIINRPIAEVLGLK-DTS 318
L.jensenii_ZP06338918           HMTDGVIATDRHGNVIILNQMALSFLDIKE-KDAVSKPITKILGLDDDVS 319
L.jensenii_ZP04646123           HMTDGVIATDRHGNVIILNQMALSFLDIKE-KEAVNKPITKILGLADDVS 319
L.delbrueckii_ZP07092088        HMTDGVIATDRHGNVSIMNQMAQDFLDVKA-EDAIKKPISQVLGLGEDYT 333
L.gasseri_YP813915              HMTDGVIATDRHGNITIINETALDFLGKTE-KDVIGKPITNLLGLK-DVT 320
```

```
L.johnsonii_NP964082                HMTDGVIATDRHGNITIINETALDFLGKTE-KDVIGKPITNLLGLK-DVT 315
L.iners_ZP05743701                  HMTDGVIATDRHGNITIINETALNFLDKTE-NEVKGEPITQLLGLK-EVT 315
E.casseliflavus_ZP05644942          HMTDGVVATDRRGKVITINEMAMSLLNVSN-EEAIGQSILTLLEIDEEYT 319
E.gallinarum_ZP05649732             HMTDGVVATDRRGKVITINEMAMSLLNVTS-EEAVGQSILTLLQIDEEYT 319
E.faecium_ZP03982596                HMTDGVIATDRRGKVITINDMAVSLLDVKN-EEAIGQSILTLLDIEEEYT 317
E.faecalis_NP814923                 HMTDGVIATDRRGKVITINEMALSLLNVKN-ENVIGTSLLELLDIEEDYT 315
Lc.lactis_YP808469                  YMVDGVIATDRRGNIIMANKSALHYLNTSN-ELLMEKNIVDVLKIADQYN 169
Lc.lactis_YP001031763               YMVDGVIATDRRGNIIMANKSALHYLNTSN-ELLMEKNIVDVLKIADQYN 169
Lc.lactis_NP266552                  YMVDGVIATDRRGNIIMANKSALHYLNTDN-DLLMQKNIVEVLKITDQYN 169
                                    :* ***:**:* * :  *  .   :.          :   *

S.mutans_NP721861                   YNDLIT-KTPEIVLTRRDEY--DEFITLRIRFALNRRESGFISGLIAVLH 196
S.mutans_YP003484506                YNDLIT-KTPEIVLTRRDEY--DEFITLRIRFALNRRESGFISGLIAVLH 196
S.gallolyticus_YP003430968          YHDLVS-KTPEIVLNRRDET--GEFITLRIRFALNRRDSGFISGLIAVLH 197
S.bovis_ZP07467090                  YHDLVS-KTPEIVLNRRDGT--GEFITLRIRFALNRRDSGFISGLIAVLH 197
S.infantarius_ZP02921024            YHDLVS-KTPEIVLNRRDET--GEFITLRIRFALNRRDSGFISGLIAVLH 197
S.dysgalactiae_YP002996286          YRELVS-KTPVVTVNSRNDM--GEFVTLRLRFALNRRESGFISGLVVVLH 197
S.pyogenes_NP268803                 YRDLVS-KTPVVTVNSRNDM--GEFVSLRLRFALNRRESGFISGLVVVLH 197
S.equi_YP002744127                  YRELVS-KTPIVTLNRRNDT--GEFVSLRLRFALNRRESGFISGLVVVLH 197
S.equi_YP002123744                  YRELVS-KTPIVTLNRRNDT--GEFVSLRLRFALNRRESGFISGLVVVLH 197
S.uberis_YP002561898                YRELVS-KTPIVTLNRRDET--GEFITLRLRFALNRRESGFISGLVVVLH 197
S.agalactiae_NP687735               FQDLVS-KTPEVVLNRRDEN--GEFVTLRIRFALNRRESGFISGLVAVSH 196
S.thermophilus_YP139610             YSELLS-KTPEIHLSRRDAN--DEFVTLRVNFALIRKESGFISGLVAVLH 196
S.salivariu_ZP04062119              YSELLS-KTPEIHLSRRDAN--DEFVTLRVNFALIRKESGFISGLVAVLH 196
S.sanguinis_YP001035504             LRDLIT-NVPELTIDSQDEN--GEYLSLRVRFALVRRESGFISGLVAVLH 197
S.gordonii_YP001450079              LRDLIT-KMPELTIDSQDEN--GEYLSLRVRFALVRRESGFISGLVAVLH 197
S.parasanguinis_ZP06900160          LRSLIT-EVPELTIDSQDEN--GEYISLRVRFALIRRESGFISGLVAVLH 197
S.oralis_ZP06612220                 LRDLIT-QIPELTIDSQDVN--GEYLSLRVRFALVRRESGFISGLVAVLH 197
S.mitis_ZP07462753                  LRDLIT-QIPELTIDSQDVN--GEYLSLRVRFALVRRESGFISGLVAVLH 197
S.oralis_ZP07458504                 LRDLIT-QIPELTIDSQDAN--GEYLSLRVRFALVRRESGFISGLVAVLH 197
S.mitis_YP003446287                 LRDLIT-QIPELMIDSQDAN--GEYLSLRVRFALVRRESGFISGLVAVLH 197
S.pneumoniae_NP358699               LRDLIT-QSPELLLDSQDIN--GEYLNLRVRFALIRRESGFISGLVAVLH 197
S.suis_ZP03624685                   LRDLLA-QTPEIVIDHTNEN--EEFLTLRANFATIRSESGLISGLVVVLH 197
S.suis_YP001198724                  LRDLLA-QTPEIVIDHTNEN--EEFLTLRANFATIRSESGLISGLVVVLH 197
L.reuteri_YP001270633               VRQLVNGEQREMIIDMSNS---GNNLILNAYFSPIQRESGFVSGLVCVLH 366
L.vaginalis_ZP03959893              VRQLVNSEQKEMILDMSNS---GSNLILNAYFSPIQRESGFVSGLVCVLH 366
L.antri_ZP05745002                  VRELINSNQKEMIIDMSAG---GNDLILNAYFSPIQRESGFISGLVCVLH 365
L.fermentum_ADJ40691                IRHLVDEDQDQVILDDLN-----EDLILSAYFSPIKRESGFVSGLVCVLH 365
P.acidilactici_ZP06196171           LRDLLE-NQDPEVLDLSND---EQDLILHASFALIQRESGFISGLVCVLH 366
P.pentosaceus_YP805259              LRDLLE-NQEQVVLDLSDD---DQDLILHASFALIQRESGFISGLVCVLH 366
L.plantarum_NP783897                LRDLIE-NQDELMLDFSSN---ERDLILNAYFSLIQRESGFISGLVCVLH 370
L.brevisgi_YP794237                 LRELLE-DPDDLVLDFSTK---EHDLILQAYFSLIQRESGFISGLVCVLH 366
L.salivarius_ZP04009519             LRDLLE-KQDEVVLDMSGE----KDLILNAYFSVIQRESGFISGIVCVLH 371
L.ruminis_ZP03957718                MRELIE-NQDGIIVDMSDA---GHDQILNAYFSTIQRESGFISGLVCVLH 370
L.casei_YP807963                    LRDLLE-HQTELLLDFSEQL--DHELILRVDFSLIQRETGFISGLVAVLH 368
L.rhamnosus_ZP04439975              LRDLLE-HQTELLLDFSEQL--DHDLILRVDFSLIQRETGFISGLVAVLH 368
L.sakei_YP394689                    LRDLLE-TQQVMVLDFSDQQ--DHDLILHVDFSLIQRETGYITGLVCVLH 363
L.acidophilus_ZP04020787            SQDLIS-SQKEIVITLDEGT--RDEMILHASFSLIKRVTGFVSGSVCVLH 365
L.ultunensis_ZP04012109             SQDLIS-SQKEIVITIDEGT--RDEMILHASFSLIKRVTGFVSGSVCVLH 368
L.crispatus_ZP03996903              SQDLIS-SQKEIVITLDPGT--RDEIILHASFSLIKRVTGFVSGSVCVLH 365
L.helveticus_YP001576656            SQDLIS-SQKEIVVTVKPGT--RDEIILHASFSLIKRVTGFVSGSVCVLH 365
L.jensenii_ZP06338918               AQELIG-NQQEMMITVNEGT--PDEMILHANFSLIRRVTGFVSGAVCVLH 366
L.jensenii_ZP04646123               AQELIG-NQQEMMITVNEGT--PDEMILHASFSLIRRVTGFVSGAVCVLH 366
L.delbrueckii_ZP07092088            SQDLIS-NQSDMQVTVNAGT--DDEIILHASFTLIKRVTGFVSGAVCVLH 380
L.gasseri_YP813915                  IQDLLS-TQQELVVRVNDNT--RDEMILHANFSLIQRVTGFVSGLVCVLH 367
L.johnsonii_NP964082                IQDLLS-TQQELVVRVNDNT--RDEMILHANFSLIQRVTGFVSGLVCVLH 362
L.iners_ZP05743701                  IQDLMS-TQQELIVTANVNT--HDELILHADFSLIQRVTGFVSGLICVLH 362
E.casseliflavus_ZP05644942          LRKLLE-NPEDMLLERPSNDLTGTNMILRIDFSMIRRESGFISGLVAVMH 368
E.gallinarum_ZP05649732             LRKLLE-SPDEMLIERPNNDIVGTNLILRIDFSMIRRESGFISGLVAVMH 368
E.faecium_ZP03982596                LRKLLE-DPNEMVLDRSTSTLESDQMILRVDFSMIRRESGFISGLVAVLH 366
E.faecalis_NP814923                 LRKLLE-EPDELLIDRSTSDREEDQMIIRVDFTMIRRESGFITGLVCVLH 364
Lc.lactis_YP808469                  FYDLLE-KEPEITIETHDAG--NKSISLHIKFALFRRESGFISGIIAVLH 216
Lc.lactis_YP001031763               FYDLLE-KEPEITIETHDAG--NESISLHIKFALFRRESGFISGIIAVLH 216
Lc.lactis_NP266552                  FYDLLE-KEPEITIETHDSG--NESISLHIKFALFRRESGFISGIIAVLH 216
                                       *:       :            :   *:  : :*::**: * :*

                                                      H box
S.mutans_NP721861                   DATEQEKEERERRLFVSNVSHELRTPLTSVKSYLEALDDGALTES-VAPS 245
S.mutans_YP003484506                DATEQEKEERERRLFVSNVSHELRTPLTSVKSYLEALDDGALTES-VAPS 245
S.gallolyticus_YP003430968          DTTEQEKEDRERRLFVSNVSHELRTPLTSVKSYLEALDEGALKED-IAPS 246
S.bovis_ZP07467090                  DTTEQEKEDRERRLFVSNVSHELRTPLTSVKSYLEALDEGALKED-IAPS 246
S.infantarius_ZP02921024            DTTEQEKEDCERRLFVSNVSHELRTPLTSVKSYLEALDEGALKED-IAPS 246
S.dysgalactiae_YP002996286          DTTEQEKEERERRLFVSNVSHELRTPLTSVKSYLEALDEGAIKED-IAPS 246
S.pyogenes_NP268803                 DTTEQEKEERERRLFVSNVSHELRTPLTSVKSYLEALDEGALKED-IAPS 246
S.equi_YP002744127                  DTTEQEKEERERRLFVSNVSHELRTPLTSVKSYLEALDEGALKED-IAPS 246
```

```
S.equi_YP002123744              DTTEQEKEERERRLFVSNVSHELRTPLTSVKSYLEALDEGALKED-IAPS 246
S.uberis_YP002561898            DTTEQEKEERERRLFVSNVSHELRTPLTSVKSYLEALDEGALKED-IAPS 246
S.agalactiae_NP687735           DATEQEKEERERRLFVSNVSHELRTPLTSVKSYLEALDEGALNEE-VAPS 245
S.thermophilus_YP139610         DATEQEKEERERRLFVSNVSHELRTPLTSVKSYLEALDDGALNEE-IAPN 245
S.salivariu_ZP04062119          DATEQEKEERERRLFVSNVSHELRTPLTSVKSYLEALDDGALDEE-IAPN 245
S.sanguinis_YP001035504         DTTEQDKEERERRLFVSNVSHELRTPLTSVKSYLEALDEGALSEP-VAPD 246
S.gordonii_YP001450079          DTTEQEKEERERRLFVSNVSHELRTPLTSVKSYLEALDEGALSEP-VAPE 246
S.parasanguinis_ZP06900160      DTTEQDKEERERRLFVSNVSHELRTPLTSVKSYLEALDDGALSEP-VAPD 246
S.oralis_ZP06612220             DTTEQEKEERERRLFVSNVSHELRTPLTSVKSYLEALDEGALYDP-VAPD 246
S.mitis_ZP07462753              DTTEQEKEERERRLFVSNVSHELRTPLTSVKSYLEALDEGALYDP-VAPD 246
S.oralis_ZP07458504             DTTEQEKEERERRLFVSNVSHELRTPLTSVKSYLEALDEGALYDP-VAPD 246
S.mitis_YP003446287             DTTEQEKEERERRLFVSNVSHELRTPLTSVKSYLEALDEGALSEP-VAPD 246
S.pneumoniae_NP358699           DTTEQEKEERERRLFVSNVSHELRTPLTSVKSYLEALDEGALCET-VAPD 246
S.suis_ZP03624685               DMTEQAKEERERRLFVSNVSHELRTPLTSVKSYLEALDEGALTES-VAPS 246
S.suis_YP001198724              DMTEQAKEERERRLFVSNVSHELRTPLTSVKSYLEALDEGALTES-VAPS 246
L.reuteri_YP001270633           DVTSQQKEERERKQFVSNVSHELRTPLTSVRSYVEALSDGAWQDKEIAPQ 416
L.vaginalis_ZP03959893          DVTSQQKEEQERKEFVSNVSHELRTPLTSVKSYVEALSDGAWQDKEIAPQ 416
L.antri_ZP05745002              DVTSQQKEERERKQFVSNVSHELRTPLTSVHSYVEALSDGAWKDQDIAPQ 415
L.fermentum_ADJ40691            DVTSQQKEERERKEFVSNVSHELRTPLTSVKSYVEALSDGAINDPELAPR 415
P.acidilactici_ZP06196171       DVTEQQKIDQDRKRFVSNVSHELRTPLTSMKSYIEALVDGAWKDPNVAPN 416
P.pentosaceus_YP805259          DVTEQQKIDQDRKRFVSNVSHELRTPLTSMKSYIEALVEGAWKDPEVAPN 416
L.plantarum_NP783897            DVTEQQKIDNDRKQFVSNVSHELRTPLTSLRSYIEALSDGAWKDPEVAPG 420
L.brevisgi_YP794237             DVTEQQKIDQARREFVSNVSHELRTPLTSVRSYIEALSEGAWKDPKVAPD 416
L.salivarius_ZP04009519         DVTEQQKIDRERKQFVSNVSHELRTPLTSMRSYIEALNDGAWRDEKVAPE 421
L.ruminis_ZP03957718            DVTEQQKIDRERRQFVSNVSHELRTPLTSMRSYIEALNDGAWKDPEVAPS 420
L.casei_YP807963                DVTEQQKIDRDRKEFVSNVSHELRTPLTSVRSYIEALSDGAWKDPKLAPR 418
L.rhamnosus_ZP04439975          DVTEQQKIDRDRKEFVSNVSHELRTPLTSVRSYIEALSDGAWKDPKLAPQ 418
L.sakei_YP394689                DVTEQQKNEREQREFVSNVSHELRTPLTSVRSYIEALNDGAWQDPEVAPN 413
L.acidophilus_ZP04020787        DVTEQQKNENSQRQFVSNVSHELRTPLTSLQAYIEALNEGAWKDPEIAPK 415
L.ultunensis_ZP04012109         DVTEQQKNEDSQRQFVSNVSHELRTPLTSLQAYIEALNEGAWKDPKIAPK 418
L.crispatus_ZP03996903          DVTEQQKNEDSQRQFVSNVSHELRTPLTSLQAYIEALNEGAWKNPEIAPK 415
L.helveticus_YP001576656        DVTEQQKNENSQRQFVSNVSHELRTPLTSLQAYIEALNEGAWKDPEIAPK 415
L.jensenii_ZP06338918           DVTEQQKNENEQRQFVSNVSHELRTPLTSLRAYIEALNDGAWKDPEIAPQ 416
L.jensenii_ZP04646123           DVTEQQKNENEQRQFVSNVSHELRTPLTSLRAYIEALNDGAWKDPEIAPQ 416
L.delbrueckii_ZP07092088        DVTEQIKNEDEQRQFVSNVSHELRTPLTSVRAYIEALNEGAWKDPEIAPQ 430
L.gasseri_YP813915              DITQQQKNEREQQQFVSNVSHELRTPLTSLRAYVEALNDGAWKDPNIAPQ 417
L.johnsonii_NP964082            DITQQQKNEREQQQFVSNVSHELRTPLTSLRAYVEALNDGAWKDPNIAPQ 412
L.iners_ZP05743701              DITEQQKNENEQRQFVSNVSHELRTPLTSLRAYVEALNDGAWKNENIAPQ 412
E.casseliflavus_ZP05644942      DVTEQEKNEQERREFVSNVSHELRTPLTSMRSYIEALSEGAWKDPEIAPN 418
E.gallinarum_ZP05649732         DVTEQEKNEQERREFVSNVSHELRTPLTSMRSYIEALSEGAWQDPEIAPN 418
E.faecium_ZP03982596            DVTEQEKTERERREFVSNVSHELRTPLTSMRSYIEALSEGAWKDEEVAPN 416
E.faecalis_NP814923             DVTEQEKNERERREFVSNVSHELRTPLTSMRSYIEALSEGAWENPEIAPN 414
Lc.lactis_YP808469              DMTEQDKAERERRLFVSNVSHELRTPLTSVKAYLEALEDGAIDDKETASS 266
Lc.lactis_YP001031763           DMTKQDKAERERRLFVSNVSHELRTPLTSVKAYLEALEDGAIDDKETASS 266
Lc.lactis_NP266552              DMTEQDKAERERRLFVSNVSHELRTPLTSVKAYLEALEDGAIEDKETASS 266
                                * *.* * :  :: ***************:::*:*** :**  :   *.

S.mutans_NP721861               FIKVSLDETNRMMRMITDLLSLSRIDNQTSHLDVELTNFTAFMNYILDRF 295
S.mutans_YP003484506            FIKVSLDETNRMMRMITDLLSLSRIDNQTSHLDVELTNFTAFMNYILDRF 295
S.gallolyticus_YP003430968      FIKVSLDETNRMIRMISDLLNLSRIDNQTVQLEVEMTNFTAFMTSILNRF 296
S.bovis_ZP07467090              FIKVSLDETNRMIRMISDLLNLSRIDNQTVQLEVEMTNFTAFMTSILNRF 296
S.infantarius_ZP02921024        FIKVSLDETNRIIRMISDLLNLSRIDNQTVQLEVEMTNFTAFMTSILNRF 296
S.dysgalactiae_YP002996286      FIKVSLDETNRMMRMISDLLNLSRIDNQVTQLSVEMTNFTAFMTSILNRF 296
S.pyogenes_NP268803             FIKVSLDETNRMMRMISDLLNLSRIDNQVTQLAVEMTNFTAFITSILNRF 296
S.equi_YP002744127              FIKVSLDETNRMMRMISDLLNLSRIDNHVTQLSVEMTNFTAFMTSILNRF 296
S.equi_YP002123744              FIKVSLDETNRMMRMISDLLNLSRIDNHVTQLSVEMTNFTAFMTSILNRF 296
S.uberis_YP002561898            FIKVSLDETNRMMRMISDLLNLSRIDNQVTALAVEMTNFTAFMTSILNRF 296
S.agalactiae_NP687735           FIKVSLDETNRMMRMISDLLSLSRIDNEVTHLDVEMTNFTAFMTSILNRF 295
S.thermophilus_YP139610         FIKVSLDETNRMMRMITDLLALSRIDNKSTQLNVEMTNFTAFMTYILNRF 295
S.salivariu_ZP04062119          FIKVSLDETNRMMRMISDLLALSRIDNKSTQLDVEMTNFTAFMTYILNRF 295
S.sanguinis_YP001035504         FVKVSLNETNRMMRMVTDLLSLSRIDNETSHLEVELTNFTAFITFILNRF 296
S.gordonii_YP001450079          FVKVSLTETNRMMRMVSDLLSLSRIDNDSSHLDVELTNFTAFITFILNRF 296
S.parasanguinis_ZP06900160      FVKVSLNETNRMMRMVTDLLSLSRIDNETSQLDIELTNFTAFITFILNRF 296
S.oralis_ZP06612220             FIKVSLDETNRMMRMVTDLLHLSRIDNTTSQLDVELINFTAFITFILNRF 296
S.mitis_ZP07462753              FIKVSLDETNRMMRMVTDLLHLSRIDNATTQLDVELINFTAFITFILNRF 296
S.oralis_ZP07458504             FIKVSLDETNRMMRMVTDLLHLSRIDNATTKLDIELINFTAFITFILNRF 296
S.mitis_YP003446287             FIKVSLDETNRMMRMVTDLLHLSRIDNATSHLDVELINFTAFITFILNRF 296
S.pneumoniae_NP358699           FIKVSLDETNRMMRMVTDLLHLSRIDNATSHLDVELINFTAFITFILNRF 296
S.suis_ZP03624685               FVKVSLDETNRMMRMITDLLSLSRIDNQVGQIDVELINFTAFVTFILNRF 296
S.suis_YP001198724              FVKVSLDETNRMMRMITDLLSLSRIDNQVGQIDVELINFTAFVTFILNRF 296
L.reuteri_YP001270633           FLKVVQDETDRMIRMINDLLSLSRMDAGTTKLNLEYVNINELFNYILDRF 466
L.vaginalis_ZP03959893          FLKVVQDETERMIRMINDLLSLSRMDAGTTKLNLEYVNINELFNYILNRF 466
L.antri_ZP05745002              FLTVIQNETNRMIRMINDLLSLSRMDSGTTKLNLEYVNIKELFNYILNRF 465
L.fermentum_ADJ40691            FLGVVSDETERMIRMINDLLSLSRMDSGTAKLNLEYVNINELFNYILNRF 465
P.acidilactici_ZP06196171       FLKVTQEETDRMMRMINDLLNLSRMDLGTARLDKEYVNLNELFNHILDRF 466
```

```
P.pentosaceus_YP805259          FLKVTQEETDRMMRMINDLLNLSRMDLGTARLEREYVNLNELFNHILDRF 466
L.plantarum_NP783897            FLKVTQEETDRMIRMINELLSLSRMDSGTTRVDMELVNINEMFNYVLDRF 470
L.brevisgi_YP794237             FLKVTQEETDRMIRMINDLLTLSRMDSGTQKVDLEMVNLNELFNYVLDRF 466
L.salivarius_ZP04009519         FLKVTQEETDRMIRMINDLLSLSRMDSGTVKLDLELVNINGLVNYILDRF 471
L.ruminis_ZP03957718            FLKVTQDETDRMIRMINDLLSLSRMDSGTVKLDFELVNLNELFNYILNRF 470
L.casei_YP807963                FLNVTQEETDRMIRMINDLLSLSRLDSGTSKFNLETVNLNEFFNYVLDRF 468
L.rhamnosus_ZP04439975          FLNVTQEETDRMIRMINDLLSLSRLDSGTSKFNLETVNLNEFFNYVLDRF 468
L.sakei_YP394689                FLKVTQEETDRMIRMINDLLSLSRMDQGTAKMNLEFVNLNEFFGYVLDRF 463
L.acidophilus_ZP04020787        FLEVTQQETGRMIRMINDLLSLSRMDRGVSKMDLEFVNLNDFVNHILNRF 465
L.ultunensis_ZP04012109         FLEVTQQETSRMIRMINDLLSLSRMDRGVSKMDLEFVNLNDFVNHILNRF 468
L.crispatus_ZP03996903          FLEVTQQETSRMIRMINDLLSLSRMDRGVSKMDLEFVNLNDFVNHILNRF 465
L.helveticus_YP001576656        FLEVTQQETSRMIRMINDLLSLSRMDRGVSKMDLEFVNFNDFVSHILNRF 465
L.jensenii_ZP06338918           FLEVTQEETERMIRMINDLLSLSRMDRGVVKMDLEWVNFNDFLSHVLNRF 466
L.jensenii_ZP04646123           FLEVTQEETERMIRMINDLLSLSRMDRGVAKMDLEWVNFNDFLSHVLNRF 466
L.delbrueckii_ZP07092088        FLDVTQKETERMIRMVNDLLVLSRMDRGATKLEPEWVNFTDFVSHLLNRF 480
L.gasseri_YP813915              FLHVIQDETERMIRMINDLLSLSRMDRGVARMDLEWVNLNDFVNHVLNRF 467
L.johnsonii_NP964082            FLHVIQDETERMIRMINDLLSLSRMDRGVAKMDLEWVNLNDFVNHVLNRF 462
L.iners_ZP05743701              FLGVIQNETERMIRMINDLLNLSRMDRGVVKMDLEWVNLNDFLSHILNRF 462
E.casseliflavus_ZP05644942      FLKVTLDETDRMIRMINDLLSLSRMDSGNSQLQLEYINFNEMVNFVLDRF 468
E.gallinarum_ZP05649732         FLKVTLDETDRMIRMINDLLSLSRMDSGNAQLQLEYINFNELVSFVLDRF 468
E.faecium_ZP03982596            FLKVTLDETDRMIRMINDLLNLSRMDTGNTQLQLEYVNFNELVNFVLDRF 466
E.faecalis_NP814923             FLKVTLEETDRMIRMINDLLNLSRMDSGNTHLQLEYVNFNELINFVLDRF 464
Lc.lactis_YP808469              FIGVSLTETNRMIRMISDLLTLSRMDQDRIVLNKEIINLIAFLDYQINRL 316
Lc.lactis_YP001031763           FIGVSLTETNRMIRMISDLLTLSRMDQDRIVLNKEIINLIAFLDYQINRL 316
Lc.lactis_NP266552              FINVSLTETNRMIRMISDLLTLSRMDQDRIVLNKEMINLIAFLDYQINRL 316
                                *: *   ** *::**:.:** ***:*     .  *  *:  :.   ::*:

S.mutans_NP721861               DQIQSQQS-------------------TNKVYEIIRDYPDKSVWIEIDTDK 327
S.mutans_YP003484506            DQIQSQQS-------------------TNKVYEIIRDYPDKSVWIEIDTDK 327
S.gallolyticus_YP003430968      DQIKSQHT------------------ISGKQYEIVRDYPIKSIWLEIDPDK 329
S.bovis_ZP07467090              DQIKSQHT------------------ISGKQYEIVRDYPIKSIWLEIDPDK 329
S.infantarius_ZP02921024        DQIKSQHT------------------VAGKQYEIVRDYPIKSIWVEIDPDK 329
S.dysgalactiae_YP002996286      DLVKNQQT------------------GTGKVYEIVRDYPITSVWLEIDNDK 329
S.pyogenes_NP268803             DLVKNQHT------------------GTGKVYEIVRDYPITSVWIEIDNDK 329
S.equi_YP002744127              DLVKNQHT------------------STGKSYEIVRDYPITSIWLEIDNDK 329
S.equi_YP002123744              DLVKNQHT------------------STGKSYEIVRDYPITSIWLEIDDDK 329
S.uberis_YP002561898            DLVRNQNT------------------VSGKSYEIIRDYPITSVWLEIDNDK 329
S.agalactiae_NP687735           DQIRNQKT------------------VTGKVYEIVRDYPLKSIWVEIDTDK 328
S.thermophilus_YP139610         GQIKSQET------------------NPGKSYEIIRDYPVNSIWVEIDTDK 328
S.salivariu_ZP04062119          DQIKSQET------------------NTGKTYEIIRDYPVNSIWVEIDTDK 328
S.sanguinis_YP001035504         DKIKNQ--------------------DETKKYEIIRDYPITPIWVEIDTDK 327
S.gordonii_YP001450079          DKIKSQ--------------------DETKKYEIVRDYPITPIWVEIDTDK 327
S.parasanguinis_ZP06900160      DKIKSQSQ------------------EDTKKYELIREYPITPIWVEIDTDK 329
S.oralis_ZP06612220             DKMRSQ--------------------DDEKKYELVRDYPINSVWIEIDTDK 327
S.mitis_ZP07462753              DKMRSQ--------------------DDEKKYELVRDYPINSVWIEIDTDK 327
S.oralis_ZP07458504             DKMRNQ--------------------DEEKKYELVRDYPITSVWIEIDTDK 327
S.mitis_YP003446287             DQIRGQ--------------------DEEKKYELVRDYPITSVWIEIDTDK 327
S.pneumoniae_NP358699           DKMKGQ--------------------EKEKKYELVRDYPINSIWMEIDTDK 327
S.suis_ZP03624685               DQMKNA--------------------DSDKVYTIVRDYQISPIWVEIDTDK 327
S.suis_YP001198724              DQMKNA--------------------DSDKVYTIVRDYQISPIWVEIDTDK 327
L.reuteri_YP001270633           DMIIKKEE---DPK--------------KKKYTIERYFTKKDLWVEIDTDK 500
L.vaginalis_ZP03959893          DMIIKKEE---DPK--------------KKKYTIERFFTKKDLWVEIDTDK 500
L.antri_ZP05745002              DMIIKKNE---NNKD-------------SKKYTIERYFTDKDLWVEIDTDK 500
L.fermentum_ADJ40691            DMIIKNEA---ADPS-------------KKKYTIVRYFTKKDLWVEIDTDK 500
P.acidilactici_ZP06196171       DMILKNGE---KSE---------------KNYTIKRDFTRRDIWVEVDTDK 499
P.pentosaceus_YP805259          DMILKNSD---KPD---------------KNYSIKRDFTRRDIWVEVDTDK 499
L.plantarum_NP783897            DMILKKDD---NPA---------------KYYTIKREFTKRDLWVEIDTDK 503
L.brevisgi_YP794237             DMMLKQDD---QPE---------------KTYTIKRDFTKRDLWVEIDTDR 499
L.salivarius_ZP04009519         DMMLKKDKIDTRDIK-------------TKNYTIKRDFTKRDLWVELDTDK 509
L.ruminis_ZP03957718            DMMLKKDNNDTRETK-------------SKNYTIKRDFTKRDLWVEIDTDK 508
L.casei_YP807963                DMMLKTDNEHTGEAKTAQPLPQP-DGQKKRYSIKREITDQDLWVEIDTDK 517
L.rhamnosus_ZP04439975          DMMLKTDNEHTGEARTAQPLPQP-DGQKKRYSIKREITHEDLWVEIDTDK 517
L.sakei_YP394689                DMMIKRDQEHDNNASPRGTGKLPTDDPNKKYSITRDFTKRDLWVEIDTDK 513
L.acidophilus_ZP04020787        DMIVKTD----KNKD-----------HKKKYTIKRELGNQALWVEIDTDK 500
L.ultunensis_ZP04012109         DMIVKTD----KNKS-----------HKKKYTINRELGSQALWVEIDTDK 503
L.crispatus_ZP03996903          DMIVKTD----KNK------------EKKKYTIKRELGSQALWVEIDTDK 499
L.helveticus_YP001576656        DMIVKTD----KNKG-----------HKKKYTIKRELGNQALWVEIDTDK 500
L.jensenii_ZP06338918           DMIVKND---EKDQT-----------GQKKYSIKRKITNQDLWVEIDTDQ 502
L.jensenii_ZP04646123           DMIVKKDA--EKGQE-----------GEKKYSIKRKITNQDLWVEIDTDQ 503
L.delbrueckii_ZP07092088        DMIVEHDQ--GEAQS-----------DTKEYVIKRDLGNQALWVEIDTDQ 517
L.gasseri_YP813915              DMMLKSD----SDKM-----------HKKKYTIKREFPHQALWVEIDTDK 502
L.johnsonii_NP964082            DMMLKSD----TDKT-----------HKKKYSIKREFPHQALWVEIDTDK 497
L.iners_ZP05743701              DMMLKND----G---------------VKKYKIRRIFPHQALWVEIDTDK 493
E.casseliflavus_ZP05644942      DMMVTNES--------------------KKYLIRREFTKRELWVEVDTDK 498
E.gallinarum_ZP05649732         DMMVTNEN--------------------KKYVIRREFTKRELWVEVDTDK 498
```

```
E.faecium_ZP03982596            DMMVGNQE--------------------KNYKIRREFTQRDLWVEVDTDK 496
E.faecalis_NP814923             DMMIENEQ--------------------KNYKICREFTKRDLWVELDTDK 494
Lc.lactis_YP808469              DQILETESN---------------DLISSNFTLVRKYSDKPIWVEIDTDK 351
Lc.lactis_YP001031763           DQILETESN---------------DLISSNFTLVRKYSDKPIWVEIDTDK 351
Lc.lactis_NP266552              DQILETESN---------------DLISSNFTLVRKYSEQPIWVEIDTDK 351
                                . :                         . : : *      :*:*:* *:

                                      N box                          G1 box
S.mutans_NP721861               MTQVIDNILNNAIKYSPDGGKVTITMQTTDTQLILSISDQGLGIPKKDLP 377
S.mutans_YP003484506            MTQVIDNILNNAIKYSPDGGKVTVTMQTTDTQLILSISDQGLGIPKKDLP 377
S.gallolyticus_YP003430968      MTQVLDNILNNAIKYSPDGGKITVSMKTTETQLIISISDEGLGIPKKDLP 379
S.bovis_ZP07467090              MTQVLDNILNNAIKYSPDGGKITVSMKTTETQLIISISDEGLGIPKKDLP 379
S.infantarius_ZP02921024        MTQVLDNILNNAIKYSPDGGKITVSMKTTETQLIISISDEGLGIPKKDLP 379
S.dysgalactiae_YP002996286      MTQVIENILNNAIKYSPDGGKITVRMKTTDTQLIISISDQGLGIPKKDLP 379
S.pyogenes_NP268803             MTQVIENILNNAIKYSPDGGKITVRMKTTDTQLIISISDQGLGIPKTDLP 379
S.equi_YP002744127              MTQVIENILNNAIKYSPDGGKIIVNMKTTDTQLIISISDEGLGIPKKDLP 379
S.equi_YP002123744              MTQVIENILNNAIKYSPDGGKIVVNMKTTDTQLIISISDEGLGIPKKDLP 379
S.uberis_YP002561898            MTQVIENILNNAIKYSPDGGKIRVKMKTTDSQLIISISDQGLGIPKKDLP 379
S.agalactiae_NP687735           MTQVIDNILNNAVKYSPDGGKITVNLRTTKTQMILSISDQGLGIPKKDLP 378
S.thermophilus_YP139610         MTQVIDNILNNAIKYSPDGGKITVSMKTTDTQLIVSISDQGLGIPKKDLP 378
S.salivariu_ZP04062119          MTQVVDNILNNAIKYSPDGGKITVSMKTTDSQLIVSISDQGLGIPKKDLP 378
S.sanguinis_YP001035504         LTQVIDNIMNNAIKYSPDGGTITVSIKTTDEQLILSIADEGLGIPKQDLP 377
S.gordonii_YP001450079          MTQVIDNIMNNAIKYSPDGGKITVGMKTTEDQLIISISDEGLGIPKKDLP 377
S.parasanguinis_ZP06900160      MTQVIDNILNNAIKYSPDGGKIKVGMRTTDAQLIISISDEGLGIPKKDLP 379
S.oralis_ZP06612220             MTQVIDNILNNAIKYSPDGGKITVSMKTTDDQMILSIKDQGLGIPKQDLP 377
S.mitis_ZP07462753              MTQVIDNILNNAIKYSPDGGKITVSMKTTDDQMILSIKDQGLGIPKQDLP 377
S.oralis_ZP07458504             MTQVIDNILNNAIKYSPDGGKITVSMKTTDDQMILSISDQGLGIPKQDLP 377
S.mitis_YP003446287             MTQVIDNILNNAIKYSPDGGKITVTMKTTDDQMILSISDQGLGIPKQDLP 377
S.pneumoniae_NP358699           MTQVVDNILNNAIKYSPDGGKITVRMKTTEDQMILSISDHGLGIPKQDLP 377
S.suis_ZP03624685               MTQVLDNILNNAIKYSPDGGTITFSMKTTDSQLIVSVSDEGLGIPKADLP 377
S.suis_YP001198724              MTQVLDNILNNAIKYSPDGGTITFSMKTTDSQLIVSVSDEGLGIPKADLP 377
L.reuteri_YP001270633           FTQVIDNIMNNAIKYSPDGGVITTRLLETHNHVILSISDQGLGIPRKDLG 550
L.vaginalis_ZP03959893          FTQVIDNIMNNAIKYSPDGGVITARLLETHNHVIMSISDQGLGIPRKDLS 550
L.antri_ZP05745002              FTQVIDNIMNNAIKYSPDGGVITTRLLETHNHVILSISDQGLGIPRKDLG 550
L.fermentum_ADJ40691            FTQVVDNIMNNAVKYSPDGGVITARLLETHNHVILSISDQGLGIPRKDLG 550
P.acidilactici_ZP06196171       IQQVLDNIMNNAIKYSPDGGVITCRLVETHNHVIMSITDQGLGIPKEAIS 549
P.pentosaceus_YP805259          IQQVLDNIMNNAIKYSPDGGIITCRLLETHNHVIMSITDQGLGIPKDSIS 549
L.plantarum_NP783897            FTQVLDNIMNNAIKYSPDGGVVTCRLLETHNQVIISISDQGLGIPRADLG 553
L.brevisgi_YP794237             FTQVLDNLMNNAIKYSPDGGVVTARLLETHNNVILSVSDQGLGIPRADIP 549
L.salivarius_ZP04009519         FIQVVDNIMNNAIKYSPDGGVITCRLLETHNNVILSISDQGLGIPKKDLG 559
L.ruminis_ZP03957718            FIQVVDNIMNNAIKYSPDGGVITCRLLETHNHVILSITDQGLGIPRKDLG 558
L.casei_YP807963                FMQVIDNIMNNAIKYSPDGGTITARLFQTHDHVILSISDQGLGIPRQDLG 567
L.rhamnosus_ZP04439975          FMQVIDNIMNNAIKYSPDGGVITARLFQTHDHVILSISDQGLGIPRQDLG 567
L.sakei_YP394689                FMQVVDNIMNNAIKYSPDGGVITCRLLETHRHVILSISDQGLGIPRKDLG 563
L.acidophilus_ZP04020787        MMQVIDNIMNNAIKYSPDGGVITVRLTQNQNHVILSISDQGLGIPRKDLA 550
L.ultunensis_ZP04012109         MMQVIDNIMNNAIKYSPDGGVITVRLTQNQNHVILSISDQGLGIPRKDLG 553
L.crispatus_ZP03996903          MMQVIDNIMNNAIKYSPDGGVITVRMTQNQNHVILSISDQGLGIPRKDLG 549
L.helveticus_YP001576656        MMQVIDNIMNNAIKYSPDGGVITVRLTHSQGHVILSISDQGLGIPRKDLG 550
L.jensenii_ZP06338918           MMQVIDNIMNNAIKYSPDGGVITVGLTQSQNQIILSISDQGLGIPKKDLN 552
L.jensenii_ZP04646123           MMQVIDNIMNNAIKYSPDGGVITVGLSQSQNQIILSISDQGLGIPKKDLN 553
L.delbrueckii_ZP07092088        MMRVIDNIMNNAIKYSPDGGTITVRLTQNQNQILLSISDQGLGIPRKDLG 567
L.gasseri_YP813915              MMQVIDNIMNNAIKYSPDGGVITVRLLQAQKHVILSISDQGLGIPRKDLN 552
L.johnsonii_NP964082            MMQVIDNIMNNAIKYSPDGGVITVRLLQAQKHVILSISDQGLGIPRKDLN 547
L.iners_ZP05743701              MMQVIDNIMNNAIKYSPDGGLITIRLLQTHKHVILSVSDQGLGIPRKDLS 543
E.casseliflavus_ZP05644942      MIQVIDNIMNNAIKYSPDGGIITVKLMETHNNIVLSINDQGLGIPKKDLT 548
E.gallinarum_ZP05649732         MIQVIDNIMNNAIKYSPDGGVITVKLMETHNNIVLSINDQGLGIPKKDLT 548
E.faecium_ZP03982596            IIQVVDNIMNNAIKYSPDGGTITCRLLETHNNVILSITDQGLGIPKKDLN 546
E.faecalis_NP814923             VIQVLDNILNNAIKYSPDGGVITCRLVETHNNVVFSISDQGLGIPKKDLG 544
Lc.lactis_YP808469              IAQVIDNIIGNAIKYSPKGGTITISIETRERDVLISIKDEGMGIPKSDLL 401
Lc.lactis_YP001031763           IAQVIDNIIGNAIKYSPKGGTITIYVETRERDVLISIKDEGMGIPKSDLL 401
Lc.lactis_NP266552              IAQVIDNIIGNAIKYSPKGGTITISVEKRESDVLISISDQGMGIPKSDLL 401
                                . :*::*::.**:****.** :   :   . .::.*: *.*:***:  :

                                    F box         G2 box                     G3 box
S.mutans_NP721861               LIFDRFYRVDKA-RSRAQGGTGLGLAIAKEIVKQHKGFIWANSE-EGEGS 425
S.mutans_YP003484506            LIFDRFYRVDKA-RSRAQGGTGLGLAIAKEIVKQHKGFIWANSE-EGEGS 425
S.gallolyticus_YP003430968      LIFDRFYRVDKA-RSRAQGGSGLGLAIAKEIVKQHKGFIWAQST-YGKGS 427
S.bovis_ZP07467090              LIFDRFYRVDKA-RSRAQGGSGLGLAIAKEIVKQHKGFIWAQST-YGKGS 427
S.infantarius_ZP02921024        LIFDRFYRVDKA-RSRAQGGSGLGLAIAKEIVKQHKGFIWAQST-YGKGS 427
S.dysgalactiae_YP002996286      LIFDRFYRVDKA-RSRAQGGTGLGLAIAKEIIKQHHGFIWAKSD-YGKGS 427
S.pyogenes_NP268803             LIFDRFYRVDKA-RSRAQGGTGLGLAIAKEIIKQHHGFIWAKSD-YGKGS 427
S.equi_YP002744127              LIFDRFYRVDKA-RSRAQGGTGLGLAIAKEIIKQHRGFIWAKSD-YGKGS 427
S.equi_YP002123744              LIFDRFYRVDKA-RSRAQGGTGLGLAIAKEIIKQHRGFIWAKSD-YGKGS 427
S.uberis_YP002561898            LIFDRFYRVDKA-RSRAQGGTGLGLAIAKEIVKQHNGFIWAKSD-YGKGS 427
S.agalactiae_NP687735           LIFDRFYRVDKA-RSRKQGGTGLGLSIAKEIVKQHKGFIWAKSE-YGKGS 426
```

```
S.thermophilus_YP139610       LIFDRFYRVDKA-RSRAQGGTGLGLSIAKEIVKQHNGFIWAKSE-YGKGS 426
S.salivariu_ZP04062119        LIFDRFYRVDKA-RSRAQGGTGLGLSIAKEIIKQHKGFIWAKSE-YGKGS 426
S.sanguinis_YP001035504       KIFDRFYRVDKA-RSRAQGGTGLGLAIAKEIIKQHQGFIWAKSE-YGVGS 425
S.gordonii_YP001450079        KIFDRFYRVDKA-RSRAQGGTGLGLAIAKEIIKQHHGFIWAKSE-YEVGS 425
S.parasanguinis_ZP06900160    RIFDRFYRVDKA-RSRAQGGTGLGLAIAKEIIKQHKGFIWAKSE-YGKGS 427
S.oralis_ZP06612220           KIFDRFYRVDRA-RSRAQGGTGLGLAIAKEIIKQHNGFIWAKSE-YGKGS 425
S.mitis_ZP07462753            KIFDRFYRVDRA-RSRAQGGTGLGLAIAKEIIKQHNGFIWAKSE-YGKGS 425
S.oralis_ZP07458504           RIFDRFYRVDRA-RSRAQGGTGLGLAIAKEIIKQHNGFIWAKSE-YGKGS 425
S.mitis_YP003446287           RIFDRFYRVDRA-RSRAQGGTGLGLAIAKEIIKQHNGFIWAKSE-YGKGS 425
S.pneumoniae_NP358699         RIFDRFYRVDRA-RSRAQGGTGLGLSIAKEIIKQHKGFIWAKSE-YGKGS 425
S.suis_ZP03624685             RIFDRFYRVDKA-RSRAQGGTGLGLAIAKEIVKQHKGFIWAKSE-YGHGS 425
S.suis_YP001198724            RIFDRFYRVDKA-RSRAQGGTGLGLAIAKEIVKQHKGFIWAKSE-YGHGS 425
L.reuteri_YP001270633         RIFDRFFRVDKA-RSRKQGGTGLGLAISKEVINMLGGQIWVDSV-EGKGS 598
L.vaginalis_ZP03959893        HIFDRFFRVDKA-RSRKQGGTGLGLAISKEVVNMLGGQIWVDSV-EGKGS 598
L.antri_ZP05745002            RIFDRFFRVDKA-RSRKQGGTGLGLAISKEVVNLLGGQIWVDSV-EGQGS 598
L.fermentum_ADJ40691          HIFDRFFRVDKA-RSRKQGGTGLGLAISKEVVNLLGGQIWVDSV-EGKGS 598
P.acidilactici_ZP06196171     HVFDRFYRVDKA-RSRAQGGTGLGLAISKEVVQMHGGRIWVESR-EGEGS 597
P.pentosaceus_YP805259        HIFDRFYRVDKA-RSRAQGGTGLGLAISKEVIQLHGGRIWVESR-EGEGS 597
L.plantarum_NP783897          HVFDRFFRVDKA-RSRAQGGTGLGLAISKEVVQMLGGRIWVDSV-EGKGS 601
L.brevisgi_YP794237           RIFDRFYRVDKA-RSRAQGGTGLGLAISREVVQMLGGRIWVDSR-EGRGS 597
L.salivarius_ZP04009519       HVFDRFFRVDKA-RSRAQGGTGLGLAISKEVIELHHGRIWVDSV-EGKGS 607
L.ruminis_ZP03957718          HIFDRFFRVDKA-RSRAQGGTGLGLAISKEVIEMHHGKIWVDSI-EGKGS 606
L.casei_YP807963              KIFDRFYRVDKA-RSRKQGGTGLGLAISKEVVEALHGRIWVDSQ-EGRGS 615
L.rhamnosus_ZP04439975        KIFDRFYRVDKA-RSRKQGGTGLGLAISKEVVEALHGRIWVDSQ-EGRGS 615
L.sakei_YP394689              KIFDRFYRVDKA-RSRKQGGTGLGLAISKEVIEAHNGRVWVDSQ-EGKGS 611
L.acidophilus_ZP04020787      KIFDRFYRVDKA-RSRAQGGTGLGLAIAKEIVEAHHGRIWADSS-EGKGS 598
L.ultunensis_ZP04012109       KIFDRFYRVDKA-RSRAQGGTGLGLAIAKEIVEAHHGRIWADSS-EGRGS 601
L.crispatus_ZP03996903        KIFDRFYRVDKA-RSRAQGGTGLGLAIAKEIVEAHHGKIWADSS-EGKGS 597
L.helveticus_YP001576656      KIFDRFYRVDKA-RSRAQGGTGLGLAIAKEIVEAHHGRVWADSS-EGKGS 598
L.jensenii_ZP06338918         KIFDRFYRVDKA-RSREQGGTGLGLAIAKEIVEAHKGKIWADSQ-EGKGS 600
L.jensenii_ZP04646123         KIFDRFYRVDKA-RSREQGGTGLGLAIAKEIVEAHKGRIWADSQ-EGKGS 601
L.delbrueckii_ZP07092088      KIFDRFYRADKA-RSRAQGGTGLGLAIAKEIVTAHGGRIWADSR-EGHGS 615
L.gasseri_YP813915            KIFDRFYRVDKA-RSRKQGGTGLGLAISKEIVEAHHGRIWADSA-EGAGS 600
L.johnsonii_NP964082          KIFDRFYRVDKA-RSRKQGGTGLGLAISKEIVEAHHGRIWADSA-EGSGS 595
L.iners_ZP05743701            RIFDRFYRVDKA-RSRKQGGTGLGLAISKEIVEAHHGKIWADSS-EGNGS 591
E.casseliflavus_ZP05644942    KIFDRFYRVDKA-RARQQGGTGLGLAISREVIKAHGGAIWAESR-ENRGS 596
E.gallinarum_ZP05649732       KIFDRFYRVDKA-RARQQGGTGLGLAISREVIKSHGGAIWAESR-ENRGS 596
E.faecium_ZP03982596          RVFERFYRVDKA-RARAQGGTGLGLAISREVIKAHRGAIWAESK-EGKGS 594
E.faecalis_NP814923           KVFERFYRVDKA-RARAQGGTGLGLAISKEVIRAHNGSIWVEST-EGEGS 592
Lc.lactis_YP808469            KIFDRFYRVDNASRNSKVGGTGLGLSIVHDIVKIHGGTIFASSEGEGKGT 451
Lc.lactis_YP001031763         KIFDRFYRVDNASRNSKVGGTGLGLSIVHDIVKIHGGTIFASSEGEGKGT 451
Lc.lactis_NP266552            KIFDRFYRVDNASRNSKVGGTGLGLSIVHDIVKIHGGTIFASSEGEGKGT 451
                               :*:**:*.*.* *    **:****:* ::::    * ::..*     *:

S.mutans_NP721861             TFTIVLPYEND--NDAIDEWEEDE----DES 450
S.mutans_YP003484506          TFTIVLPYEND--NDAIDEWEEGE----DES 450
S.gallolyticus_YP003430968    TFTIVLPYEKD--SEIYDEWEDEE----E-- 450
S.bovis_ZP07467090            TFTIVLPYEKD--SEIYDEWEDEE----E-- 450
S.infantarius_ZP02921024      TFTIVLPYEKD--SDIYDEWEDDE----D-- 450
S.dysgalactiae_YP002996286    TFTIVLPYEKD--AAIYEEWEDDI----D-- 450
S.pyogenes_NP268803           TFTIVLPYEKD--AAIYEEWEEDV----D-- 450
S.equi_YP002744127            TFTIVLPYEKD--AAIYDEWEDDI----D-- 450
S.equi_YP002123744            TFTIVLPYEKD--AAIYDEWEDDI----D-- 450
S.uberis_YP002561898          TFTIVLPYEKDVVAGTDDEWEDDI----D-- 452
S.agalactiae_NP687735         TFTIVLPYDKD--AVTYEEWEDVE----D-- 449
S.thermophilus_YP139610       TFTIVLPYDQD--AMMVDEWEM--------- 446
S.salivariu_ZP04062119        TFTIVLPYDKD--AMMVDEWEM--------- 446
S.sanguinis_YP001035504       TFTIVLPYEND--GVRDDDWDNE-----DDI 449
S.gordonii_YP001450079        TFTIVLPYDKD--AVLDDGWESEEVESKDD- 453
S.parasanguinis_ZP06900160    TFTIVLPYDKD--AIKDD-WDTEE----EE- 450
S.oralis_ZP06612220           TFTIVLPYDKD--AVKEEIWEDEI----ED- 449
S.mitis_ZP07462753            TFTIVLPYDKD--AVKEEIWEDEI----ED- 449
S.oralis_ZP07458504           TFTIVLPYDKD--AVKEEIWEDEI----ED- 449
S.mitis_YP003446287           TFTIVLPYDKD--AVKEEVWEDEV----ED- 449
S.pneumoniae_NP358699         TFTIVLPYDKD--AVKEEVWEDEV----ED- 449
S.suis_ZP03624685             TFTIVLPYSKD--IALDEWDDSDE----EE- 449
S.suis_YP001198724            TFTIVLPYSKD--IALDEWDDSDE----EE- 449
L.reuteri_YP001270633         TFYISLPYVPYE--E--EDWDDEEAEK---- 621
L.vaginalis_ZP03959893        TFYISLPYVPYE--EG-DEWDDQKDQN---- 622
L.antri_ZP05745002            TFYISLPYVPYE--EE-DDWDD--------- 617
L.fermentum_ADJ40691          TFYISLPYVPYE--E--EEWDDQAD------ 619
P.acidilactici_ZP06196171     TFYISLPYEPFE--EG-DAWE---------- 615
P.pentosaceus_YP805259        TFYISLPYEPFE--EG-DTWE---------- 615
L.plantarum_NP783897          TFYISLPYEPYE--EE-DLWDDDSQA----- 624
L.brevisgi_YP794237           TFYISLPYKPYD--EE-DLWDEDN------- 618
```

```
L.salivarius_ZP04009519         TFYISLPYEEYS--EE-DLWDEN-------- 627
L.ruminis_ZP03957718            TFYISLPYEEYE--GD-DLWDEE-------- 626
L.casei_YP807963                TFYISLPYEPIT--DGGDDWDEA-------- 636
L.rhamnosus_ZP04439975          TFYISLPYEPIT--DGGDDWDEA-------- 636
L.sakei_YP394689                TFYISLPYEPID--SDGGDWDEI-------- 632
L.acidophilus_ZP04020787        TFYISLPYEPMT--EEDD-WDEI-------- 618
L.ultunensis_ZP04012109         TFYISLPYEPMT--EEDD-WDEV-------- 621
L.crispatus_ZP03996903          TFYISLPYEPMT--EEDD-WDEV-------- 617
L.helveticus_YP001576656        TFYISLPYETMT--EEDD-WDEV-------- 618
L.jensenii_ZP06338918           TFYISLPFEPMS--EEDD-WDEV-------- 620
L.jensenii_ZP04646123           TFYISLPFEPMS--EEDD-WDEV-------- 621
L.delbrueckii_ZP07092088        TFYIALPYEPMD--EEDE-WDEV-------- 635
L.gasseri_YP813915              TFYISLPYEAIS--EEGENWDEV-------- 621
L.johnsonii_NP964082            TFYISLPYEAIS--EEGENWDEV-------- 616
L.iners_ZP05743701              TFYISIPYEKMS--EEGDMWDEV-------- 612
E.casseliflavus_ZP05644942      TFYIMLPYEPYE--ED--WWV---------- 613
E.gallinarum_ZP05649732         TFYIMLPYEPYE--ED--WWE---------- 613
E.faecium_ZP03982596            TFYISLPYEPYE--EE--WWE---------- 611
E.faecalis_NP814923             TFYISLPYEPYE--ED--WWE---------- 609
Lc.lactis_YP808469              TFSFTLPYSSETAEYWDDAADDFE------- 475
Lc.lactis_YP001031763           TFSFTLPYSSETSEYWDDAADDFE------- 475
Lc.lactis_NP266552              TFSFTLPYSLEAADDWDDGTDEFEFEDNE-- 480
                                ** : :*:
```
