# Supplementary material for: Mechanistic Insights Revealed by the Crystal Structure of a Histidine Kinase with Signal Transducer and Sensor Domains
Source: PLoS Biol. 2013 Feb 26;11(2):e1001493. doi: 10.1371/journal.pbio.1001493 (PMC3582566; doi:10.1371/journal.pbio.1001493)
Supplement: Table S2 — Statistics of data collection and structure refinement. †The data for the highest resolution shell are shown in parenthesis. *Rsym = ∑|I−|/∑I, where I is the observed intensity, is the statistically weighted average intensity of multiple observations of symmetry-related reflections. ††I/σ(I) – ratio of mean intensity to a mean standard deviation of intensity. ‡R = ∑||Fo|−|Fc||/∑|Fo|, where Fo and Fc are observed and calculated structure factor amplitudes, respectively. **Rfree is calculated using 5% of randomly selected reflections. ***Number of protein atoms of the ordered regions. §Rmsd – root mean square deviation. (DOC) [file pbio.1001493.s014.doc]

| Data | Native | Se-SAD |
| --- | --- | --- |
| Space Group | P3_1_21 | P3_1_21 |
| Unit Cell (Å,°) | 153.12, 153.12, 125.11, 90, 90, 120 | 152.96, 152.96, 124.88, 90, 90, 120 |
| Resolution (Å) | 50~3.3 (3.42~3.3) ^†^ | 50-3.55 (3.69-3.55) |
| *R_sym_ (%) | 7.3 (69.2) | 12.6 (72.6) |
| ^††^I/σ | 21.1 (2.8) | 67.1 (1.5) |
| Completeness (%) | 99.5 (100) | 99.9(94.1) |
| Measured reflections | 151,361 | 1,002,968 |
| Unique reflections | 25,746 | 20,687 |
| Redundancy | 5.9 (6.0) | 19.1 |
| Wilson B factor (Å^2^) | 102 | 126.5 |
| Refinement | | |
| **^‡^**R (%) | 22.02 |  |
| **R_free_ (%) | 26.90 |  |
| ***Number of atoms | | |
| Protein | 6264 |  |
| Ligand/ion | 0 |  |
| Water | 0 |  |
| Average B (Å^2^) | | |
| Main chain | 148.40 |  |
| Side chain | 154.25 |  |
| ^§^Rmsd | | |
| Bond lengths (Å) | 0.018 |  |
| Bond angles (°) | 1.413 |  |
| Ramachandran plot (%) | | |
| Most favourable | 80.1 |  |
| Additionally allowed | 16.7 |  |
| Generously allowed | 3.2 |  |
| Disallowed | 0 |  |
